# Morphine Tolerance Gated through EZH2‐Mediated Suppression of *Trpc5* in Spinal GABAergic Interneurons in Male Mice

**DOI:** 10.1002/advs.202507908

**Published:** 2025-10-20

**Authors:** Li Wan, Mengyao Zhang, Haiyue Guo, Yan Xu, Chenjie Xu, Fan Hu, Yinbing Pan, Xian Wang, Wentao Liu, Chun‐Yi Jiang

**Affiliations:** ^1^ Jiangsu Key Laboratory of Neurodegeneration Department of Pharmacology Nanjing Medical University Nanjing Jiangsu 211166 China; ^2^ State Key Laboratory of Pharmaceutical Biotechnology School of Life Sciences Nanjing University Nanjing Jiangsu 210023 China; ^3^ Department of Pain the first people ‘s hospital of Changzhou Soochow University Changzhou Jiangsu 213000 China; ^4^ Department of Anesthesiology and Pain Nanjing First Hospital Nanjing Medical University Nanjing Jiangsu 210006 China; ^5^ Department of Anesthesiology The First Affiliated Hospital of Nanjing Medical University Nanjing Jiangsu 210029 China; ^6^ Department of Anesthesiology, Women's Hospital of Nanjing Medical University Nanjing Women and Children's Healthcare Hospital Nanjing Jiangsu 210004 China

**Keywords:** EZH2, GABAergic interneurons, Morphine tolerance, TRPC5

## Abstract

A major unresolved issue in managing severe pain is tolerance caused by repeated treatment of opioid analgesics. Here, it is demonstrated that tolerance‐inducing treatment with morphine results in the persistent downregulation of transient receptor potential canonical 5 (TRPC5), impairing the Ca^2+^ homeostasis in GABAergic interneurons of the spinal dorsal horn (SDH) and consequently reducing GABA release. Spinal activation of TRPC5 by riluzole (RLZ) or lentiviral‐mediated TRPC5 overexpression in GABAergic interneurons produces a long‐lasting enhancement of morphine's analgesic effect. In contrast, pharmacological inhibition of TRPC5 and mice lacking TRPC5 accelerates the development of morphine tolerance. Mechanistically, it is found that transcriptional suppression of *Trpc5* results from enhancer of zeste homolog 2 (EZH2)‐mediated epigenetic modifications at the *Trpc5* gene promoter. Morphine decreases the enrichment of RNA polymerase II at the *Trpc5* promoter. Moreover, exposure to morphine increases EZH2 binding to the *Trpc5* promoter, leading to the enrichment of histone H3 lysine‐27 trimethylation (H3K27me3). Pharmacological blockade of EZH2 by EPZ6438 or genetic silencing in GABAergic interneurons reverses morphine tolerance. Thus, it is proposed that the clinical translation of these findings may help reduce the suffering of individuals with intractable pain.

## Graphical abstract

Chronic morphine suppresses *Trpc5* in spinal GABAergic interneurons via EZH2‐mediated histone modification, reducing Ca^2+^ influx and GABA release. TRPC5 activation enhances morphine analgesia, while EZH2 inhibition restores efficacy and reverses tolerance. These results reveal TRPC5 and EZH2 as promising therapeutic targets to overcome opioid tolerance and improve pain management.

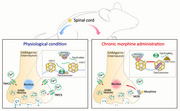

## 1 Introduction

Pain is recognized as a global health problem with a profound impact on quality life. Opioids have been essential for pain management, as endorsed by the World Health Organization (WHO). Since the 1990s, global opioid consumption has increased, with morphine being the second most consumed opioid, primarily for cancer pain relief and palliative care.^[^ ^1^, ^2^ ^]^ Morphine is widely utilized due to its affordability and strong analgesic effects, but its use is often compromised by the development of tolerance. Despite this, safe and effective therapeutic alternatives remain limited. The spinal dorsal horn (SDH) is the primary site of action for morphine's analgesic effect. Long‐term morphine exposure leads to adaptations in many signaling proteins within SDH, which have been proposed as mechanisms of analgesic tolerance.^[^ ^3^, ^4^ ^]^ Noxious information from primary afferents is processed by complex circuits in SDH, involving both excitatory and inhibitory interneurons, before being transmitted to projection neurons.^[^ ^5^ ^]^ Chronic morphine treatment induces an imbalance between excitation and inhibition in the regulation of nociceptive signaling within the SDH. Specifically, it causes the downregulation of KCC2 in spinal lamina I neurons, thereby disrupting chloride homeostasis and leading to a sustained facilitation of excitatory responses.^[^ ^6^, ^7^, ^8^ ^]^

It is well established that the periaqueductal gray (PAG) modulates nociception via a descending pathway that relays through the rostral ventromedial medulla (RVM) and terminates in the SDH.^[^ ^9^, ^10^, ^11^, ^12^, ^13^ ^]^ Long‐term morphine treatment has been shown to activate GABAergic neurons in the PAG while inhibiting glutamatergic output to RVM OFF cells. Inhibition of RVM OFF cells or activation of RVM ON cells enhances pain responses by reducing GABA release in the SDH.^[^ ^14^, ^15^, ^16^ ^]^ The inhibitory signals from GABAergic interneurons in the SDH are crucial for the analgesic effects of morphine. These interneurons regulate sensory inputs and modulate pain transmission through spinal GABA‐mediated presynaptic and postsynaptic inhibition.^[^ ^5^, ^17^ ^]^ Intrathecal injection of morphine is widely used in clinical settings and has been shown to be superior to various anesthetic techniques in providing pain relief and prolonging analgesic effects.^[^ ^18^, ^19^ ^]^ However, considerable clinical evidence suggests that intrathecal morphine administration still causes analgesic tolerance.^[^ ^20^, ^21^, ^22^ ^]^ Moreover, selective silencing of GABAergic interneurons in SDH abrogates the analgesic effect of morphine, whereas pharmacological activation of spinal GABA_A_ receptors significantly improves morphine tolerance.^[^ ^23^ ^]^ This implies that the local administration of morphine in the spinal cord may directly suppress GABAergic interneurons, an effect independent of the PAG‐RVM descending pathway. Mechanistically, unequivocal evidence for the direct action of morphine on the SDH GABAergic interneurons is still lacking.

Here, we show that morphine induces the downregulation of TRPC5, impairing Ca^2+^ homeostasis in spinal GABAergic interneurons in mice and thereby reducing GABA release. Chronic morphine exposure leads to the transcriptional suppression of *Trpc5*. As previously reported, the TRPC5 channel functions as a Ca^2+^ entry pathway and is involved in various physiological and pathological processes, such as blood pressure regulation and the development of focal segmental glomerulosclerosis (FSGS).^[^ ^24^, ^25^ ^]^ TRPC5 also acts as a cellular sensor that responds to nociceptive stimuli. Activation of TRPC5 in peripheral sensory neurons has been shown to contribute to mechanical hypersensitivity induced by complete Freund's adjuvant (CFA) intraplantar injection or paclitaxel chemotherapy.^[^ ^26^ ^]^ However, although TRPC5 is expressed in both the dorsal root ganglia (DRG) and lamina I‐III of the spinal dorsal horn (SDH),^[^ ^27^ ^]^ its role in spinal pain modulation remains unclear. We find that TRPC5 is specifically expressed in GABAergic interneurons in the SDH. Pharmacological activation of TRPC5 or overexpression in GABAergic interneurons enhances the analgesic effects of morphine. Conversely, blockade of TRPC5 by intrathecal injection of AC1903 or genetic deletion of *Trpc5* accelerates the development of morphine tolerance. Using two‐photon imaging of the SDH in mice, we demonstrate that TRPC5 in GABAergic interneurons regulates Ca^2+^ influx and mediates GABA release. Intrathecal morphine administration significantly decreased intracellular Ca^2+^ concertation and inhibited GABA release. Mechanistically, we show that the transcriptional suppression of *Trpc5* by morphine is mediated by EZH2. Pharmacological inhibition or genetic silencing of EZH2 in the spinal cord effectively reverses morphine tolerance. As the catalytic subunit of the polycomb repressive complex 2 (PRC2), EZH2 directs the trimethylation of histone H3 at lysine 27 (H3K27me3).^[^ ^28^ ^]^ Further investigations reveal that morphine increases the binding of EZH2 to the *Trpc5* promoter, thereby suppressing *Trpc5* transcription. Thus, our findings suggest new strategies for preventing morphine tolerance through the activation of TRPC5 or the inhibition of EZH2 at the spinal level.

## 2 Results

### 2.1 Transcriptional Suppression of *Trpc5* in Spinal GABAergic Interneurons Drives Morphine Tolerance

**Figure 1:**
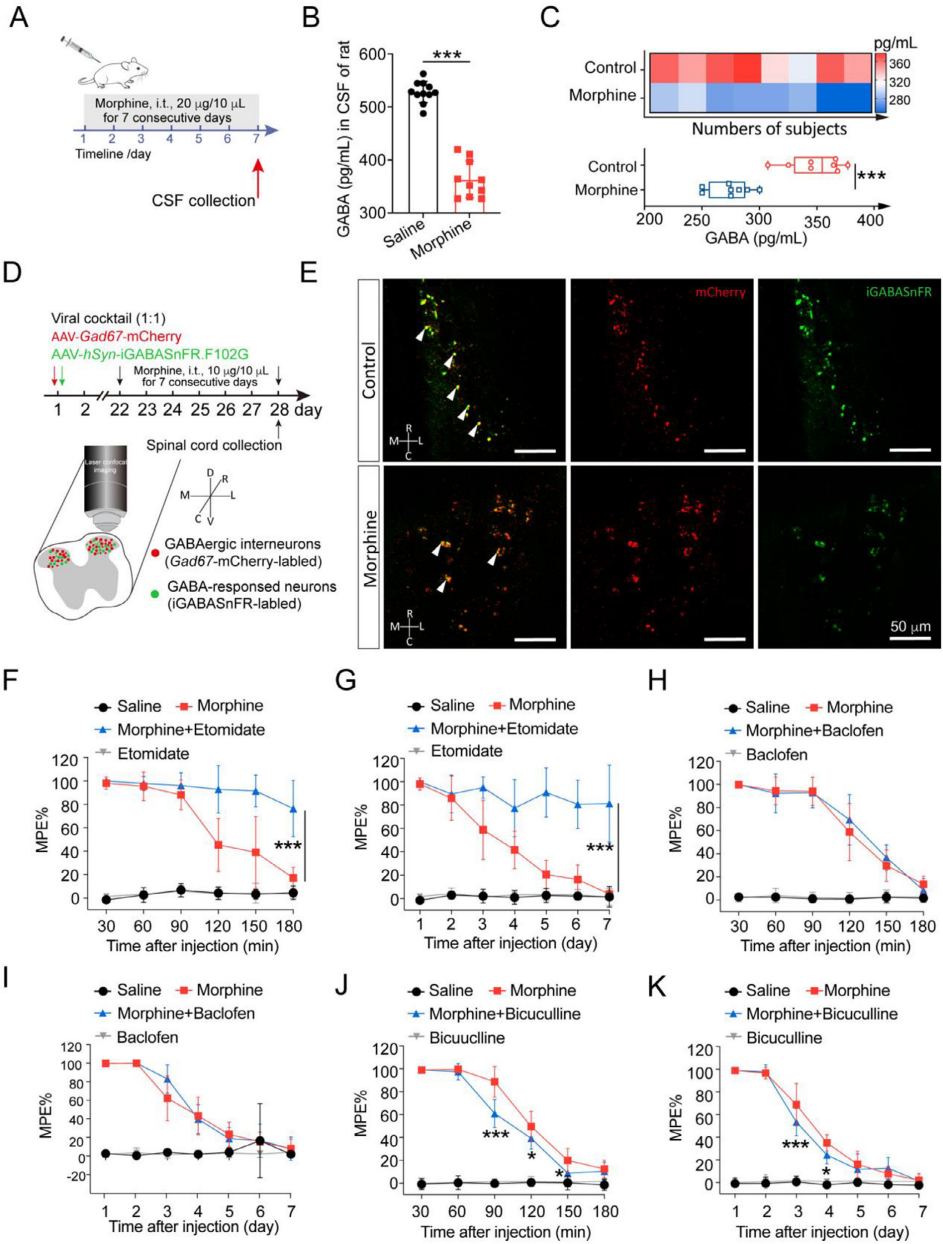
Decreased GABA release caused by intrathecal morphine injection. A) Scheme of the morphine administration regimen used for CSF collection in rats. B) GABA levels of CSF from rats after intrathecal morphine injection (20 µg/10 µL) for 7 consecutive days were reduced compared with saline‐treated group (Unpaired Student's *t* test, *t* (19) = 13.49, *P* < 0.0001, saline‐treated group: *n* = 11, morphine‐treated group: *n* = 10). C) ELISA detection revealed that the levels of GABA in the CSF from patients receiving morphine treatment were decreased compared with control subjects. (Unpaired Student's *t* test, *t* (14) = 7.180, *P* < 0.0001, *n* = 8 samples). D) Schematic illustration of the *ex vivo* experiments and neuronal labeling strategy. E) Representative images of the spinal cord L4‐L5 showed decreased GABA release (green) around GABAergic interneurons (red) after chronic morphine exposure compared to the saline‐treated group (*n* = 4). Quantitative analysis of GABA release following chronic intrathecal morphine administration was provided in Figure S1 (Supporting Information). Rostral (R), caudal (C), medial (M), lateral (L), dorsal (D), and ventral (V). F) GABA_A_ receptor agonist (Etomidate, 5 εg/10 εL, i.t.) enhanced acute morphine analgesic effect (two‐way ANOVA, drug effect: *F* (3, 168) = 654.5, *P* < 0.0001; time effect: *F* (5, 168) = 22.94, *P* < 0.0001; drug × time effect: *F* (15, 168) = 15.33, *P* < 0.0001, *n* = 8 mice). G) GABA_A_ receptor agonist (Etomidate, 5 µg/10 µL, i.t.) significantly improved morphine tolerance (two‐way ANOVA, drug effect: *F* (3, 196) = 544.8, *P* < 0.0001; time effect: *F* (6, 196) = 20.02, P < 0.0001, drug × time effect: *F* (18, 196) = 14.31, *P* < 0.0001, *n* = 8 mice). H) GABA_B_ receptor agonist (Baclofen, 0.25 µg/10 µL, i.t.) had no effect on acute morphine analgesia (two‐way ANOVA, drug effect: *F* (3, 168) = 613.3, *P* < 0.0001; time effect: *F* (5, 168) = 102.2, *P* < 0.0001; drug × time effect: *F* (15, 168) = 34.55, *P* < 0.0001, *n* = 8 mice). I) GABA_B_ receptor agonist (Baclofen, 0.25 µg/10 µL, i.t.) had no effect on chronic morphine tolerance (two‐way ANOVA, drug effect: *F* (3, 196) = 278.7, *P* < 0.0001; time effect: *F* (6, 196) = 76.44, *P* < 0.0001; drug × time effect: *F* (18, 196) = 30.25, *P* < 0.0001, *n* = 8 mice). J) GABA_A_ receptor antagonist (Bicuculline, 5 µg/10 µL, i.t.) weakened acute morphine analgesic effect (two‐way ANOVA, drug effect: *F* (3, 168) = 1037, *P* < 0.0001; time effect: *F* (5, 168) = 248.7, *P* < 0.0001; drug × time effect: *F* (15, 168) = 87.14, *P* < 0.0001, *n* = 8 mice). K) GABA_A_ receptor antagonist (Bicuculline, 5 µg/10 µL, i.t.) accelerated the development of chronic morphine tolerance (two‐way ANOVA, drug effect: *F* (3, 196) = 752.0, *P* < 0.0001; time effect: *F* (6, 196) = 274.0, *P* < 0.0001; drug × time effect: *F* (18, 196) = 91.95, *P* < 0.0001, *n* = 8 mice). Data are expressed as mean ± SD, *P < 0.05 and ***P < 0.001.

We found that tolerance‐inducing treatment with morphine by intrathecal injection decreased GABA levels in the CSF of rats (**Figure** **1**A,B). To further confirm the effects of morphine on GABA release in the spinal cord, the CSF collected from 16 patients treated with or without morphine was analyzed. All patients in the morphine group who underwent CSF collection had previously received morphine treatment. The demographic data and clinical characteristics are shown in Tables S1 and S2 (Supporting Information). Consistent with data above, GABA levels were reduced in the CSF of patients receiving morphine treatment compared with controls (Figure 1C). To visualize the changes in GABA release at spinal level, we performed *ex vivo* GABA imaging in the superficial dorsal horn in response to repeated intrathecal administration of morphine (Figure 1D). AAV‐*hSyn*‐iGABASnFR.102G and AAV‐*Gad67*‐mCherry were intrathecally injected into the spinal cord of mice. Our data showed that long‐term morphine treatment decreased GABA release (green) around GABAergic interneurons (red) in the L4‐L5 spinal cord compared with saline‐treated group (Figure 1E; Figure S1, Supporting Information). These findings suggest that morphine directly suppresses GABA release from spinal GABAergic interneurons, independent of PAG‐RVM control.

Next, we examined whether the analgesic effect of morphine could be enhanced by pharmacological activation of GABA receptors. GABA_A_ receptor agonist, etomidate, and GABA_B_ receptor agonist, baclofen, were employed. To exclude the anesthetic effects of etomidate on mice, we established a dose gradient of 2.5, 5, and 10 µg of etomidate and assessed its anesthetic impact 30 min post‐intrathecal injection using CatWalk gait analysis and the open field test. As shown in Figure S2A–C (Supporting Information), mice exhibited a reduced average speed and stride length following intrathecal injection of 10 µg etomidate. In contrast, the 2.5 and 5 µg groups displayed no significant differences in locomotor activity compared with the saline group, as assessed by CatWalk gait analysis. In the open field test, mice were placed at the center of a transparent glass box (50 × 50 × 50 cm), and their movements were recorded for 5 min using an automated video tracking system (Clever Sys Inc.). Both the total movement distance and average speed were significantly reduced following intrathecal injection of 10 µg etomidate (Figure S2D–F, Supporting Information), indicating impaired motor function and incomplete recovery from anesthesia. Behavioral analysis revealed no significant differences between the 5 µg etomidate‐treated group and the saline‐treated group, suggesting that etomidate did not exhibit analgesic effects 30 min after intrathecal administration. Based on these results, we selected 5 µg as the optimal dose to investigate the role of etomidate in modulating morphine tolerance. As shown in Figure 1F,G, GABA_A_ receptor agonist, etomidate, not only maintained analgesic efficacy of morphine throughout the 7‐day test period but also enhanced acute morphine analgesia. In contrast, GABA_B_ receptor activation had no effect on morphine tolerance (Figure 1H,I). Notably, GABA_A_ receptor antagonist significantly decreased both acute and chronic morphine analgesia (Figure 1J,K). Collectively, these results indicate that reduced GABA levels due to intrathecal morphine administration contribute to morphine tolerance.

**Figure 2:**
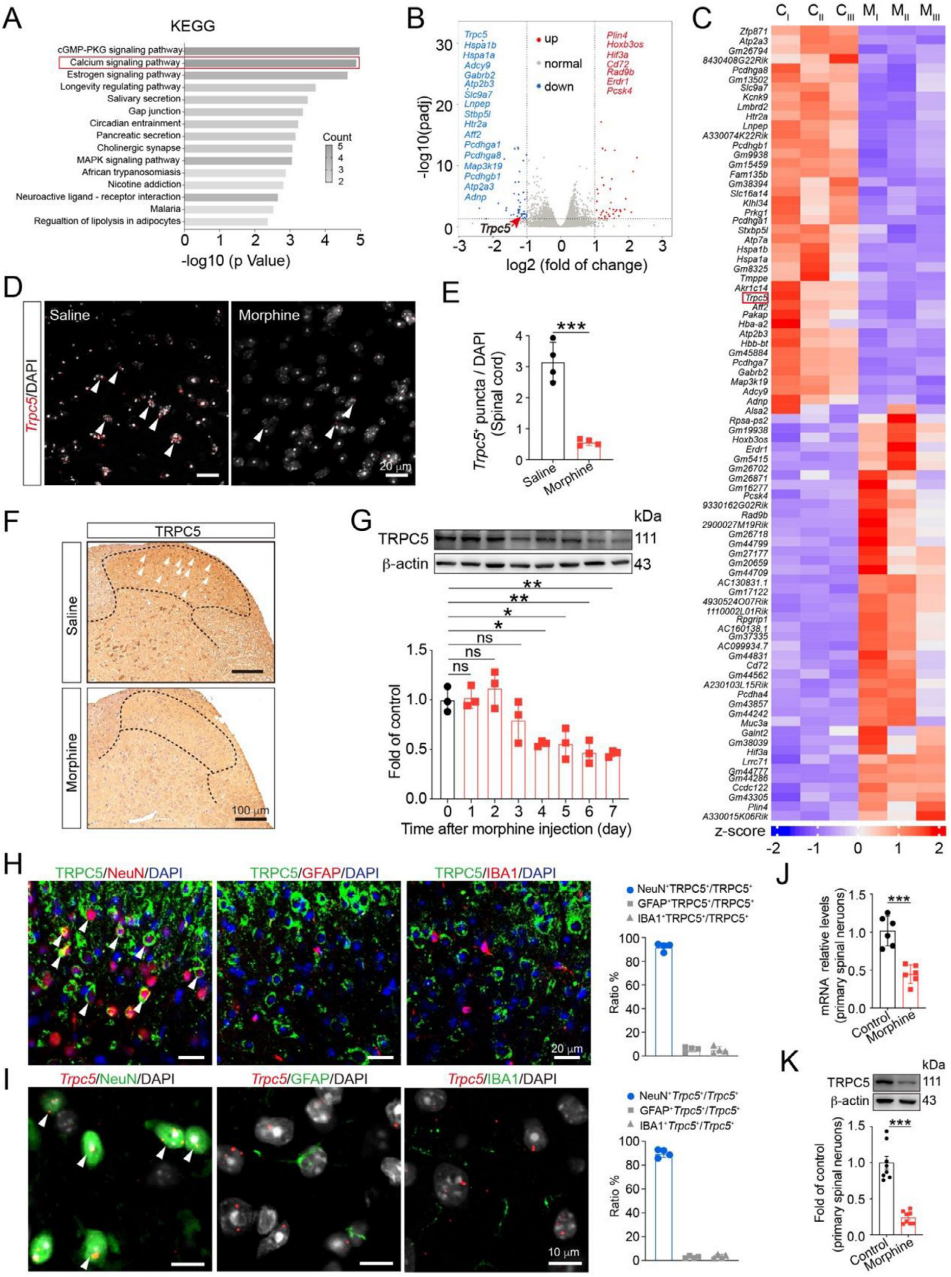
Morphine downregulates TRPC5 in the lamina I‐III of spinal dorsal horn. A) KEGG pathway analysis displayed that the most significantly enriched signaling pathways of differential expression genes in the spinal cords between morphine‐treated group and saline‐treated group. B) Volcano plot showed the results of differential expression genes analysis performed in morphine‐treated group relative to saline‐treated group. Genes related with morphine tolerance and pain are indicated. C) The heatmap showed mRNA expression in the spinal cords from mice after morphine treatment and saline treatment based on RNA sequence (*n* = 3 samples/group, each sample included 3 L4‐L5 spinal cords). Colors represent high (red) and low (blue) intensity. D) In situ hybridization RNAscope images of *Trpc5* mRNA in the spinal dorsal horn of saline‐treated group and morphine‐treated mice. White arrows indicated *Trpc5* mRNA puncta. E) Quantitative analysis showed decreased *Trpc5* ^+^ puncta per cell in the spinal dorsal horn after chronic morphine exposure compared to the saline group (Unpaired Student's *t* test, *t* (6) = 8.06, *P* = 0.0002, *n* = 4). F) Representative images of immunohistochemistry showed the downregulated protein level of TRPC5 in the spinal dorsal horn from morphine‐treated group compared with saline‐treated group. White arrows indicated TRPC5. *n* = 3. G) Immunoblotting data showed the levels of TRPC5 expression in L4‐L5 sections of the spinal cords after morphine exposure (i.t., 10 µg/10 µL) from day 0 to day 7 (one‐way ANOVA, *F* (7,16) = 8.250, *P* < 0.0001, *n* = 3). H) Representative confocal microscopy images of TRPC5 in the dorsal horn lamina I‐III of mice with neurons (NeuN), astrocytes (GFAP) and microglia (IBA‐1) (*n* = 4). I) In situ hybridization RNAscope images of *Trpc5* mRNA in neurons (NeuN), astrocytes (GFAP) and microglia (IBA‐1) (*n* = 4). J) qPCR revealed decreased mRNA levels of *Trpc5* in primary spinal neurons after morphine exposure (200 µM, 14 h) (Unpaired Student's *t* test, *t* (10) = 6.031, *P* = 0.0001, *n* = 6). K) Immunoblot analysis revealed downregulation of TRPC5 protein levels in primary spinal neurons after morphine exposure (200 µM, 14 h) (Unpaired Student's *t* test, *t* (10) = 8.000, *P* < 0.0001, *n* = 8). Data are expressed as mean ± SD, *P < 0.05, **P < 0.01 and ***P < 0.001.

To dissect the mechanisms underlying morphine‐induced inhibition of GABA release, mice were intrathecally administered with morphine for 7 consecutive days to induce analgesic tolerance. Subsequently, the spinal cords of mice were collected for RNA‐sequencing (RNA‐seq) to profile the transcriptomic differences between control and tolerant mice. Notably, Kyoto Encyclopedia Genes and Genomes (KEGG) analysis revealed the downregulation of many calcium‐related pathway genes in tolerant mice (**Figure** **2**A). Consistently, the volcano plot and heatmap showed a significant decrease in the transcription of several genes associated with calcium‐related pathways, including *Trpc5*, *Adnp*, *Atp2a3*, *Atp2b3*, and *Adcy9* (Figure 2B,C). To further validate the RNA‐seq data, quantitative real‐time PCR (qPCR) assays were performed (Figure S3A, Supporting Information).

It is well known that nociceptive transient receptor potential (TRP) channels, especially TRPV1 and TRPA1, play a critical role in pain modulation.^[^ ^29^, ^30^ ^]^ The TRPC subfamily, identified as Ca^2+^ permeable nonselective cation channels, includes TRPC5, which is primarily recognized as a cold sensing Ca^2+^ channel activated within the temperature range of 25–37 °C.^[^ ^27^, ^31^ ^]^ However, the role of TRPC5 in pain transmission within the central nervous system remains unclear. To investigate the effect of morphine on the transcription and expression of TRPC5, histochemical analyses were performed. Fluorescence in situ hybridization (FISH) data showed that morphine caused transcriptional suppression of *Trpc5* in SDH (Figure 2D,E) and also decreased TRPC5 protein levels in the SDH (Figure 2F,G). Additionally, we analyzed TRPC5 protein levels in the spinal cords of mice from both saline‐ and morphine‐treated groups during the development of morphine tolerance. Immunoblotting data revealed a gradual downregulation of TRPC5 from day 3 to day 7 in morphine‐treated mice (Figure 2G). Next, an immunofluorescence assay was performed to examine the distribution of TRPC5 in the SDH. The data showed that TRPC5 specifically colocalized with NeuN (a neuronal marker), but not GFAP (an astrocyte marker) and IBA‐1 (a microglial marker) (Figure 2H). Furthermore, *Trpc5* mRNA expression in the SDH was examined by RNAscope. Co‐staining of *Trpc5* with NeuN, GFAP and IBA‐1 revealed that 89.9% of TRPC5‐positive staining was localized to NeuN⁺ cells, while 2.9% and 3.7% were localized to IBA‐1⁺ and GFAP⁺ cells, respectively (Figure 2I). Next, we characterized the suppressive transcriptional regulation of *Trpc5* caused by morphine. Primary spinal neurons were cultured and then treated with 2, 20, and 200 µM morphine for 14 h, respectively (Figure S3B, Supporting Information). Morphine significantly suppressed both mRNA and protein levels of TRPC5 at 200 µM (Figure 2J,K). However, morphine (200 µM, 14 h) did not affect the transcription of *Trpc1* and *Trpc4* (Figure S3C, Supporting Information). Additionally, membrane proteins were isolated from primary spinal neurons after morphine stimulation (200 µM, 14 h) and immunoblotting results demonstrated a decrease of TRPC5 on cell membrane (Figure S3D, Supporting Information). To examine whether the suppressive effect of morphine on TRPC5 was opioid receptor‐dependent, neurons were pretreated with naloxone (NLX, 10 µM) for 12 h and then exposed to morphine (200 µM, 14 h). Immunoblotting and qPCR data revealed that NLX reversed the suppressive effect of morphine on TRPC5 (Figure S3E,F, Supporting Information). Next, neurons were treated with morphine (200 µM), DAMGO (µ opioid receptor agonist, 10 µM), nalfurafine (κ opioid receptor agonist, 10 µM) and ADL‐5859 (δ opioid receptor agonist, 10 µM) for 14 h, followed by immunoblotting analysis. Consistent with the effect of morphine, DAMGO significantly inhibited TRPC5 expression (Figure S3G, Supporting Information). These data indicate that morphine induces downregulation of TRPC5 in a µ opioid receptor‐dependent manner.

**Figure 3:**
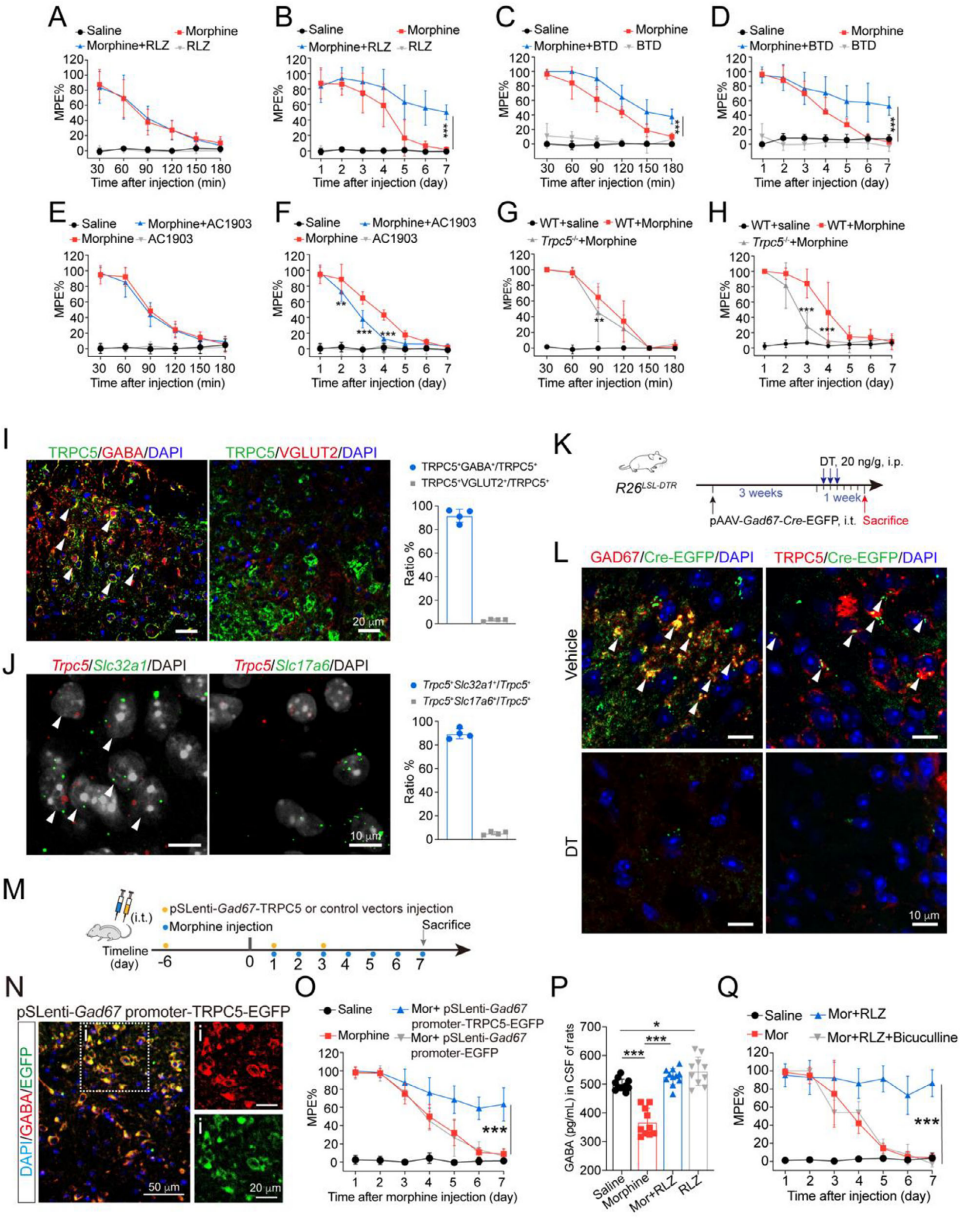
Activation of TRPC5 in GABAergic interneurons improves morphine tolerance. A) Intrathecal injection of RLZ (2 µg/10 µL) had no effect on acute morphine analgesia (two‐way ANOVA, drug effect: *F* (3, 264) = 259.9, *P* < 0.0001; time effect: *F* (5, 264) = 75.23, *P* < 0.0001; drug × time effect: *F* (15, 264) = 25.5, *P* < 0.0001, *n* = 12). B) Intrathecal injection of RLZ (2 µg/10 µL) significantly improved morphine tolerance (two‐way ANOVA, drug effect: *F* (3, 308) = 579.3, *P* < 0.0001; time effect: *F* (6, 308) = 48.87, *P* < 0.0001; drug × time effect: *F* (18, 308) = 20.25, *P* < 0.0001, *n* = 12). C) Intrathecal injection of BTD (2 µg/10 µL) significantly enhanced acute morphine analgesic effect (two‐way ANOVA, drug effect: *F* (3, 168) = 583.3, *P* < 0.0001; time effect: *F* (5, 168) = 79.89, *P* < 0.0001, drug × time effect: *F* (15, 168) = 23.77, *P* < 0.0001, *n* = 8). D) Intrathecal injection of BTD (2 µg/10 µL) significantly improved chronic morphine tolerance (two‐way ANOVA, drug effect: *F* (3, 227) = 409.5, *P* < 0.0001; time effect: *F* (6, 227) = 43.39, *P* < 0.0001, drug × time effect: *F* (18, 227) = 15.07, *P* < 0.0001, *n* = 9). E) Intrathecal injection of AC1903 (2 µg/10 µL) had no effect on acute morphine analgesia (two‐way ANOVA, drug effect: *F* (3, 264) = 610.2, *P* < 0.0001; time effect: *F* (5, 264) = 221.6, *P* < 0.0001, drug × time effect: *F* (15, 264) = 78.07, *P* < 0.0001, *n* = 12). F) Intrathecal injection of AC1903 (2 µg/10 µL) accelerated chronic morphine tolerance (two‐way ANOVA, drug effect: *F* (3, 308) = 482.5, *P* < 0.0001; time effect: *F* (6, 308) = 157.7, *P* < 0.0001, drug × time effect: *F* (18, 308) = 52.80, *P* < 0.0001, *n* = 12). G) WT and *Trpc5* ^−/−^ mice were subjected to morphine (i.t., 10 µg/10 µL) and tail‐flick test was performed 30 min after drugs injection. *Trpc5* deficiency weakened the analgesic effects of acute morphine (two‐way ANOVA, drug effect: *F* (2, 90) = 184.2, *P* < 0.0001; time effect: *F* (5, 90) = 98.94, *P* < 0.0001, drug × time effect: *F* (10, 90) = 26.11, *P* < 0.0001, n = 6, *Trpc5* ^−/−^ + morphine versus WT + morphine). H) WT and *Trpc5* ^−/−^ mice were subjected to morphine (i.t., 10 µg/10 µL) for 7 consecutive days and tail‐flick test was performed 30 min after drugs injection. *Trpc5* deficiency markedly accelerated the development of morphine tolerance (two‐way ANOVA, drug effect: *F* (2, 105) = 124.6, *P* < 0.0001; time effect: *F* (6, 105) = 60.96, *P* < 0.0001, drug × time effect: *F* (12, 105) = 19.48, *P* < 0.0001, *Trpc5* ^−/−^ + morphine versus WT + morphine). I) Double immunofluorescence showed distinctive TRPC5 (green) expression in GABAergic interneurons (GABA, red) and glutamergic interneurons (VGLUT2, red) in the spinal cord of naïve mice. J) In situ hybridization RNAscope images of *Trpc5* mRNA in inhibitory interneurons (*Slc32a1* ^+^) and excitatory interneurons (*Slc17a6* ^+^). White arrows indicate *Trpc5* double labeled with *Slc32a1* (*n* = 4). K) Scheme of the pAAV‐mediated Cre recombinase expression (EGFP^+^, green) in GABAergic interneurons and diphtheria toxin (DT) injection regimen to ablate spinal GABAergic interneurons in *R26^LSL‐DTR^* mice. L) Immunostaining of GAD67 or TRPC5 with EGFP in the spinal dorsal horn of vehicle or DTX‐treated *R26^LSL‐DTR^* mice. White arrows indicate GAD67 or TRPC5 double labeled with EGFP *(n* = 4). M) Scheme of chronic morphine exposure regimen used for tail‐flick test in mice. Mice were intrathecally injected with pSLenti‐TRPC5 or its control pSLenti‐EGFP before chronic morphine exposure (10 µg/10 µL, 7d). N) Representative photomicrographs with an inset showed pSLenti‐mediated TRPC5 expression (EGFP^+^, green) in GABAergic interneurons (GABA, red) in the spinal cord of mice. O) TRPC5 overexpression in GABAergic interneurons of spinal cord significantly improved chronic morphine tolerance (two‐way ANOVA, drug effect: *F* (3, 252) = 726.8, *P* < 0.0001; time effect: *F* (6, 252) = 34.27, *P* < 0.0001, drug × time effect: *F* (18, 252) = 34.27, *P* < 0.0001, *n* = 10). P) ELISA analysis of GABA in CSF from rats exposed to morphine (i.t., 20 µg /10 µL) with or without RLZ (i.t., 4 µg /10 µL) for 7 continuous days (one‐way ANOVA, *F* (3,38) = 44.16, *P* < 0.0001, control group: *n* = 11, morphine group: *n* = 10, morphine paired with RLZ: *n* = 11, RLZ group: *n* = 10). I) Data displayed that RLZ (i.t., 2 µg/10 µL) improved morphine tolerance and Bicuculline (i.t., 5 µg/10 µL) abolished the effect of RLZ on morphine tolerance (two‐way ANOVA, drug effect: *F* (3, 196) = 450.3, *P* < 0.0001; time effect: *F* (6, 196) = 100.2, *P* < 0.0001, drug × time effect: *F* (18, 196) = 28.73, *P* < 0.0001, *n* = 8). Data are expressed as mean ± SD, *P<0.05, **P < 0.01 and ***P < 0.001.

Next, we determined the role of TRPC5 in morphine tolerance using pharmacological and genetic approaches. Mice were intrathecally injected with morphine and a TRPC5 activator, riluzole (RLZ), for 7 consecutive days. Tail flick tests were performed to assess the effect of RLZ on the antinociceptive efficacy of morphine. Notably, intrathecal administration of RLZ alone did not produce significant analgesic effects compared with the saline‐treated group. On day 7, the MPE at 30 min post‐morphine administration decreased to 1.3% in chronic morphine‐treated mice, whereas mice co‐administered with RLZ and morphine exhibited an MPE of 43.2%. Our data indicate that RLZ could enhance chronic morphine analgesic effects without altering acute morphine antinociceptive effects (**Figure** **3**A,B). To assess the effect of RLZ treatment on the innervations of the primary afferents, immunofluorescence assays were performed. RLZ‐treated mice showed normal innervations of the primary afferents, labeled with calcitonin gene‐related peptide (CGRP), isolectin B4 (IB4) and neurofilament 200 (NF200) in the SDH (Figure S4A, Supporting Information). In the DRG, the expression of CGRP, IB4 and NF200 in RLZ‐treated mice was indistinguishable from that in saline‐treated mice (Figure S4B, Supporting Information). To further confirm the role of TRPC5 in morphine tolerance, another selective TRPC5 activator, BTD, was utilized. As shown in Figure 3C,D, BTD partially enhanced chronic morphine analgesic effect and prolonged the morphine analgesic period up to 180 min. In addition, we assessed the effect of AC1903, a selective TRPC5 inhibitor, on morphine tolerance. Data showed that intrathecal administration of AC1903 did not affect acute morphine analgesia (Figure 3E) but attenuate the analgesic effect of chronic morphine, with MPE decreasing from 43.0% to 12.7% on day 4 (Figure 3F). To further validate the role of TRPC5 in morphine tolerance, *Trpc5* ^−/−^ mice were generated (Figure S5A, Supporting Information). The deletion of 8992 base pairs between exon 4 and exon 6 of the *Trpc5* gene was confirmed by DNA sequencing (Figure S5B, Supporting Information) and PCR assay (Figure S5C,D, Supporting Information). *Trpc5* deficiency did not affect *Trpc1* and *Trpc4* mRNA levels (Figure S5E, Supporting Information). Protein levels of TRPC5 were also decreased in the spinal cord of *Trpc5* ^−/−^ mice compared with WT mice (Figure S5F, Supporting Information). *Trpc5* ^−/−^ mice exhibited normal innervations of primary afferents labeled with CGRP, IB4 and NF200, and the expression of these markers did not differ from WT mice (Figure S5G,H, Supporting Information). Next, *Trpc5* ^−/−^ and WT mice were intrathecally injected with morphine (10 µg/10 µL) for 7 consecutive days. Tail‐flick tests were conducted 30 min after morphine administration each day. Behavioral analysis revealed that *Trpc5* deficiency had no significant impact on acute morphine‐induced analgesia (Figure 3G) but accelerated the development of morphine tolerance, with the maximum MPE decreasing to 28.2% by day 3 (Figure 3H). These findings suggest that *Trpc5* deficiency in the spinal cord promotes the development of morphine tolerance. Finally, we investigated whether TRPC5 activation could enhance the analgesic effect of morphine in a neuropathic pain model. A CCI‐induced neuropathic pain mouse model was established. Fourteen days after peripheral nerve injury, mice received intrathecal injections of morphine (10 µg/10 µL), with a subset of mice also receiving RLZ (i.t., 2 µg/10 µL). Behavioral analysis showed that morphine alone produced analgesia for only two days, whereas co‐administration with RLZ markedly enhanced analgesia, increasing the mechanical pain threshold from 0.2 to 1.0 g (Figure S6, Supporting Information). These results suggest that TRPC5 activation may potentiate the therapeutic effect of morphine in CCI‐induced neuropathic pain.

Further immunofluorescence analysis indicated that TRPC5 co‐localized with the inhibitory neuronal marker GABA, but not with the excitatory neuronal marker vesicular glutamate transporter 2 (VGLUT2) (Figure 3I). To investigate the *Trpc5* mRNA expression profile across different neuronal populations, RNA‐FISH was performed using RNAscope technology on the spinal cord of mice. *Trpc5* mRNA expression was assessed in excitatory and inhibitory neurons by co‐staining with established markers: *Slc17a6* for excitatory neurons and *Slc32a1* for inhibitory neurons. The data showed that 89.5% of *Trpc5*‐positive staining was localized to *Slc32a1*⁺ cells, whereas only 5.3% was observed in *Slc17a6*⁺ cells, confirming the preferential distribution of TRPC5 in inhibitory neurons (Figure 3J). To further validate the cellular distribution of TRPC5, intrathecal injections of pAAV‐*Gad67*‐Cre‐EGFP vectors were administered into *R26^LSL‐DTR^* mice to selectively ablate GABAergic interneurons in the spinal cord. *R26^LSL‐DTR^* mice received the viral construct for three weeks, followed by daily intraperitoneal injections of diphtheria toxin (DT, 20 ng/g) or vehicle from day 1 to day 3 to eliminate spinal GABAergic interneurons (Figure 3K). Spinal cords were collected for immunofluorescence analysis one week after the initial DT injection. As shown in Figure 3L, along with the downregulation of Cre recombinase (EGFP^+^), both glutamate decarboxylase 67 (GAD67, a marker of inhibitory neurons) and TRPC5 were significantly reduced in the DT‐treated group compared to the vehicle‐treated controls. These findings suggest that TRPC5 is expressed in GABAergic inhibitory interneurons. Furthermore, primary spinal neurons were cultured for immunofluorescence assays to validate the TRPC5 expression in GABAergic interneurons. Data showed that TRPC5 was expressed in GAD67^+^ primary spinal neurons (Figure S7A,B, Supporting Information). To investigate whether overexpression of TRPC5 in GABAergic interneurons could enhance morphine analgesia, a pSLenti‐*Gad67*‐TRPC5 delivery vector was constructed, and its efficiency was evaluated by immunoblotting in primary spinal neurons. Data demonstrated that pSLenti‐*Gad67*‐TRPC5 significantly increased TRPC5 protein levels compared with the control vector (Figure S7C, Supporting Information). Subsequently, pSLenti‐*Gad67*‐TRPC5 was intrathecally injected into the spinal cord of mice 7 days prior to morphine administration (Figure 3M). Tail‐flick test results revealed that TRPC5 overexpression in GABAergic interneurons markedly enhanced the analgesic effect of morphine compared with the pSLenti‐*Gad67*‐EGFP‐treated group (Figure 3N,O). To further investigate the role of TRPC5 in spinal GABAergic interneurons in morphine tolerance, the pSLenti‐*Gad67*‐TRPC5‐WPRE viral construct was administered intrathecally into *Trpc5* ^−/−^ mice. Seven days after injection, the mice received daily intrathecal morphine administration for 7 consecutive days. Behavioral analysis showed that restoring TRPC5 expression in spinal GABAergic interneurons significantly alleviated morphine tolerance in *Trpc5* ^−/−^ mice (Figure S8, Supporting Information). Next, to examine the effect of TRPC5 activation on the morphine‐induced reduction in GABA release, rats were intrathecally injected with morphine (20 µg/10 µL), with or without RLZ (4 µg/10 µL), for 7 consecutive days. CSF was collected for GABA level measurement via ELISA. As shown in Figure 3P, RLZ effectively reversed the chronic morphine‐induced downregulation of GABA release. Notably, the GABA_A_ receptor antagonist (bicuculline, 5 µg/10 µL) prevented the RLZ‐mediated attenuation of morphine tolerance (Figure 3Q). These findings suggest that morphine‐induced TRPC5 deficiency disrupts GABA release.

### 2.2 Morphine Inhibits GABA Release via Reducing TRPC5‐Mediated Ca^2+^ Influx

**Figure 4:**
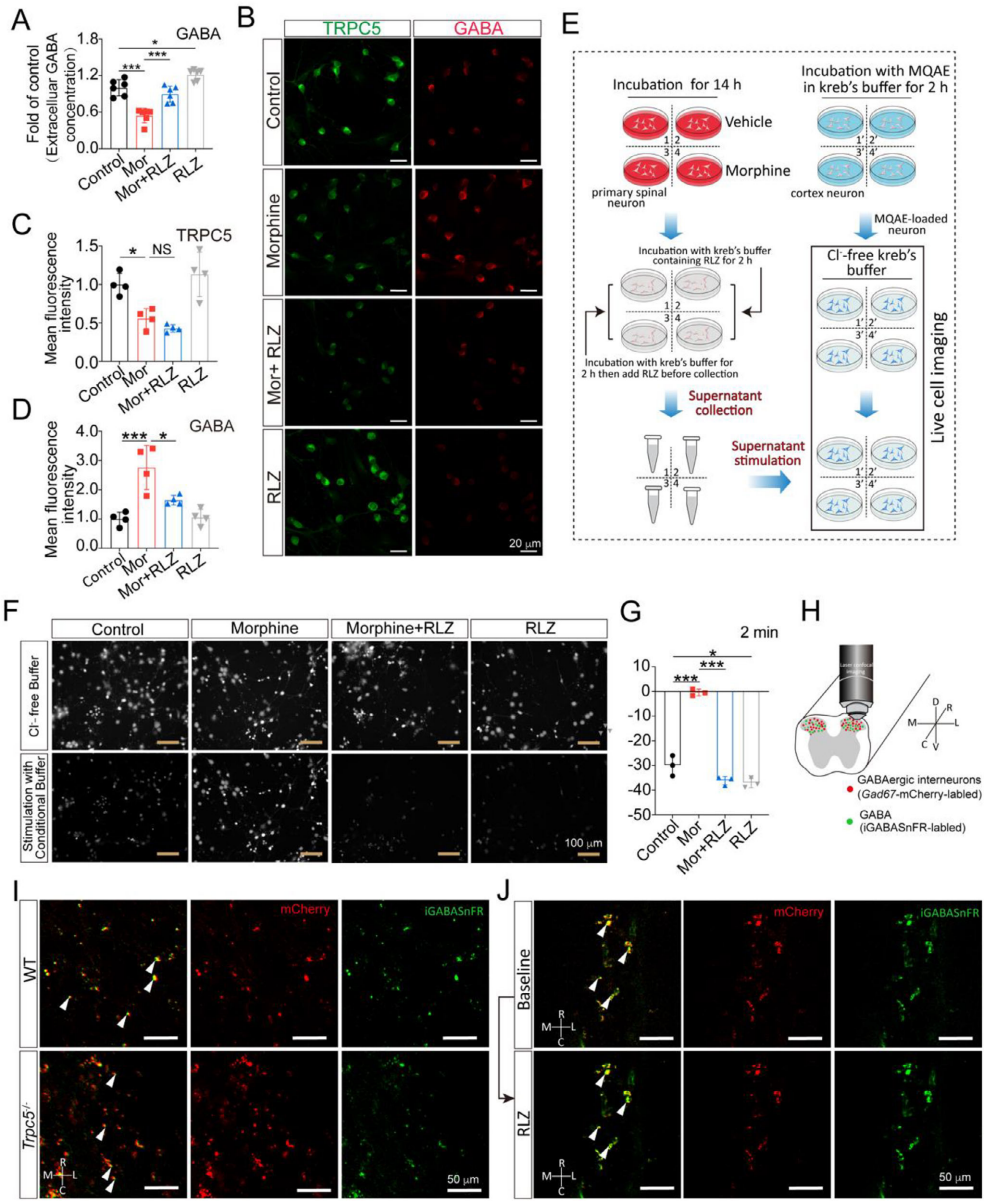
TRPC5 regulates GABA release. A–D) Primary spinal neurons were exposed to morphine (200 µM, 14 h) and then subjected to RLZ (50 µM) for 2 h. A) After 2 h, the supernatants were collected for GABA detection. ELISA analysis of GABA in collected supernatants showed that RLZ improved the decreased release of GABA caused by morphine and RLZ also promoted GABA release under basal conditions (one‐way ANOVA, *F* (3, 20) = 32.3, *P* < 0.0001, *n* = 6). B–D) Representative images of immunofluorescence (B) showed GABA and TRPC5 expression in primary spinal neurons. Fluorescence quantitative analysis (C) displayed that RLZ had no effect on morphine‐induced the downregulation of TRPC5 expression (one‐way ANOVA, *F* (3, 12) = 51.56, *P* < 0.0001, *n* = 4). Fluorescence quantitative analysis (D) displayed that RLZ improved morphine‐induced accumulation of GABA (one‐way ANOVA, *F* (3, 12) = 52.28, *P* < 0.0001, *n* = 4). E) Schematic diagram displayed the chloride imaging process in cortex primary neurons. F,G) Spinal primary neurons were administrated with morphine (200 µM, 14 h) and then treated with RLZ in kreb's buffer (50 µM, 2 h). The supernatants were collected to stimulate cortex neurons loaded with the Cl^−^‐sensitive dye MQAE (5 µM, 2 h). Supernatants from morphine‐treated group stimulation resulted in a lower rising of chloride influx and the inhibition of Cl^−^ influx was abolished by RLZ (one‐way ANOVA, *F* (3, 8) = 141.1, *P* < 0.0001, *n* = 3). H) Schematic illustration of the *ex vivo* experiments and neuronal labeling strategy. Rostral (R), caudal (C), medial (M), lateral (L), dorsal (D), and ventral (V). I) Fluorescence images of the spinal cord L4‐L5 showed decreased GABA release (green) around GABAergic interneurons (red) in *Trpc5* ^−/−^ mice compared with wild‐type mice (*n* = 4). Quantitative analysis of GABA release was provided in Figure S11A (Supporting Information). J) Two‐photon imaging of the superficial dorsal horn displaying a robust increase in GABA release one minute after incubation with RLZ (50 µM) in ACSF compared to baseline conditions (*n* = 4). Quantitative analysis of GABA release was provided in Figure S11B (Supporting Information). Data are expressed as mean ± SD, *P < 0.05 and ***P < 0.001.

Our study strongly implies the role of TRPC5 in the regulation of GABA release. To investigate the mechanisms underlying TRPC5‐mediated GABA release, primary spinal neurons were cultured and then stimulated with 200 µM morphine for 14 h. Supernatants were collected for ELISA detection to measure the extracellular levels of GABA. Data showed that the decreased GABA release by morphine was reversed by RLZ treatment in primary spinal neurons (**Figure** **4**A). Furthermore, confocal imaging revealed that morphine significantly downregulated TRPC5 expression (green) and increased intracellular GABA accumulation (red) (Figure 4B–D), indicating decreased GABA release. The reduction in GABA release by morphine was reversed by RLZ treatment and RLZ did not affect TRPC5 expression (Figure 4B–D). In addition, morphine and RLZ had no effect on the expression of GABA synthetase ‐ GAD67 (Figure S9A,B, Supporting Information), indicating that morphine and RLZ affect GABA release but not synthesis. To further assess the role of TRPC5 in GABA release in morphine tolerance, chloride imaging was performed. Conditional supernatants from primary spinal neurons were collected and applied to stimulate primary cortex neurons (Figure 4E). MQAE was utilized to detect intracellular Cl^−^ accumulation in cortex neurons. The rate of Cl^−^ accumulation was significantly lower in neurons treated with conditioned supernatants from morphine‐treated spinal neurons. It indicates that morphine induced reduction of GABA release leading to lower Cl^−^ influx. This inhibition of Cl^−^ influx caused by morphine could be abolished by RLZ (Figure 4F,G; Figure S10, Supporting Information).

To further evaluate the effect of TRPC5 on GABA release under more controlled conditions than in vivo conditions, *ex vivo* GABA imaging was performed in the superficial dorsal horn of WT and *Trpc5* ^−/−^ mice. We first confirmed that *Trpc5* deficiency had no effect on GAD67 expression in the spinal cords compared with wild‐type mice (Figure S5F, Supporting Information). Mice were then intrathecally injected with pAAV‐*hSyn*‐iGABASnFR.102G to monitor GABA and pAAV‐*Gad67*‐mCherry to label GABAergic interneurons (Figure 4H). Using two‐photon imaging of the SDH GABAergic interneurons, we found that *Trpc5* ^−/−^ mice displayed decreased GABA release in spinal cord L4‐L5 region (Figure 4I; Figure S11A, Supporting Information). Additionally, to directly assess GABA release in response to TRPC5 activation, we performed *ex vivo* GABA imaging in the superficial dorsal horn of WT mice. Specifically, mice were intrathecally injected with pAAV‐*Gad67*‐mCherry‐WPRE and pAAV‐*hSyn*‐iGABASnFR.102G. After 21 days, we conducted two‐photon imaging of the superficial dorsal horn. Within 1 min of RLZ treatment, a robust increase in GABA release was observed compared with baseline conditions (Figure 4J; Figure S11B, Supporting Information).

**Figure 5:**
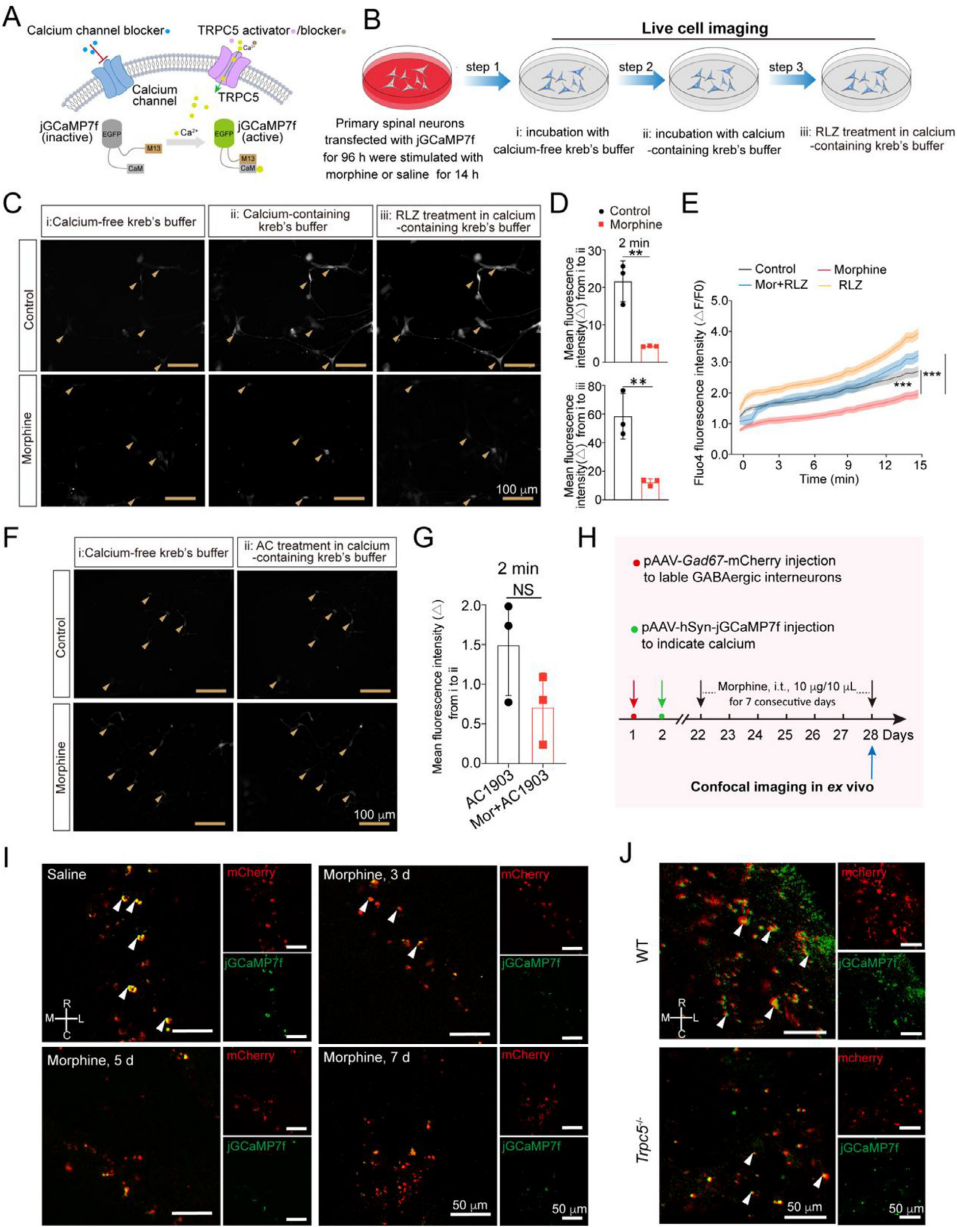
TRPC5 regulates Ca^2+^ homeostasis in GABAergic interneurons. A) Schematic diagram illustrating the principle of in vitro calcium imaging using jGCaMP7f as the calcium indicator following TRPC5 inhibition or activation. B) Schematic diagram displayed the process of calcium imaging in primary spinal neurons. C) Primary spinal neurons were transfected with calcium indicator pSLenti‐*Gad67*‐jGCaMP7f‐Puro‐WPRE and then exposed to morphine (200 µM, 14 h). Calcium imaging of primary spinal neurons displayed Ca^2+^ influx in morphine‐treated neurons and control neurons. Representative images showed (i) neurons were incubated with calcium‐free kreb's buffer; (ii) 2 min after incubation with calcium‐containing kreb's buffer; (iii) 2 min after RLZ treatment in calcium‐containing kreb's buffer. D) Fluorescence quantitative analysis displayed that decreased Ca^2+^ influx emerged in morphine‐treated neurons (from i to ii) compared with control group after incubation with calcium‐containing kreb's buffer (Unpaired Student's *t* test, *t* (4) = 5.489, *P* = 0.0054, *n* = 3). After treatment with RLZ in calcium‐containing kreb's buffer, lower rising of Ca^2+^ influx was observed (from i to iii) in morphine‐treated neurons compared with control group (Unpaired Student's *t* test, *t* (4) = 4.968, *P* = 0.0077, *n* = 3). E) SH‐SY5Y cells were exposed to morphine (200 µM, 14 h) and then incubated with Fluo‐4 indicator dyes. In the absence of calcium, the resting fluorescence (F_0_) was recorded in kreb's buffer. In the calcium‐containing kreb's buffer, fluorescence (F _test_) was recorded with or without RLZ treatment in the context of nifedipine and CaV 2.2 blocker 1. Intracellular calcium fluctuations were recorded within 15 min at an interval of 30 s (15 min: one‐way ANOVA, *F* (3, 28) = 27.28, *P* < 0.0001). F,G) Primary spinal primary neurons were transfected with calcium indicator pSLenti‐*Gad67‐*jGCaMP7f‐Puro‐WPRE and then subjected to morphine (200 µM, 14 h). F) Calcium imaging showed Ca^2+^ influx in morphine‐treated neurons and control neurons. Representative images indicated (i) neurons were incubated with the calcium‐free kreb's buffer; (ii) 2 min after AC1903 (TRPC5 antagonist, 50 µm) treatment in calcium‐containing kreb's buffer. G) Fluorescence quantitative analysis showed that there is no difference in Ca^2+^ influx between morphine‐treated group and control group after AC1903 administration in calcium‐containing kreb's buffer (Unpaired Student's *t* test, *t* (4) = 1.763, *P* = 0.1528, *n* = 3). H) Schematic illustration of *ex vivo* experiments showed neuronal labeling strategy and the construction of morphine tolerance model. I) Fluorescence images of the spinal dorsal horn showed decreased calcium (green) concentration in GABAergic interneurons (red) 3, 5, and 7 days after chronic morphine administration compared to saline‐treated group (*n* = 4). Quantitative analysis of calcium levels was provided in Figure S15A (Supporting Information). Rostral (R), caudal (C), medial (M) and lateral (L) are indicated on the white cross. J) Fluorescence images of the spinal cord L4‐L5 showed decreased calcium (green) concentration in GABAergic interneurons (red) in the spinal dorsal horn of *Trpc5* ^−/−^ mice compared to WT mice (*n* = 4). Quantitative analysis of calcium levels was provided in Figure S15B (Supporting Information). Data are expressed as mean ± SD, ***P < 0.001.

Next, to determine whether GABA release relies on TRPC5‐mediated Ca^2+^ influx, the green fluorescent protein‐based genetically encoded calcium indicator jGCaMP7f was utilized for in vitro calcium imaging before or after TRPC5 inhibition or activation, respectively (**Figure** **5**A). Primary spinal neurons were cultured and transfected with pSLenti‐*Gad67*‐jGCaMP7f‐Puro‐WPRE. The neurons were then pretreated with morphine (200 µM) for 14 h. After removing morphine, the medium was replaced with kreb's buffer containing L‐type calcium channel inhibitor (Nifedipine, 1 µM) and N‐type calcium channel inhibitor (Cav 2.2 blocker 1, 10 µM) (Figure 5B). Data showed that morphine significantly reduced Ca^2+^ influx in morphine‐pretreated neurons compared with the control group. RLZ treatment improved Ca^2+^ influx compared to morphine‐treated group (Figure 5C,D; Figure S12, Supporting Information). To evaluate the effect of morphine on calcium fluctuations in SH‐SY5Y cells, we first examined its impact on TRPC5 expression and GABA release. Immunoblotting analysis revealed that TRPC5 protein levels were downregulated only in SH‐SY5Y cells treated with 200 µM morphine (Figure S13A, Supporting Information). In addition, ELISA results showed that morphine suppressed GABA release from SH‐SY5Y cells (Figure S13B, Supporting Information). Consistent findings were also observed in primary spinal neurons exposed to morphine.

Intracellular calcium ([Ca^2+^] _i_) fluctuations were then assessed with the calcium indicator dye Fluo‐4 AM for 15 min at 30 s intervals in presence of Ca^2+^, nifedipine and CaV 2.2 blocker 1. We observed that the value of (*F _test_* ‐ *F_0_*)/(*F_0_* – *F _base_*), reflecting the [Ca^2+^] _i_, was lower in morphine‐treated neurons compared with the control group. TRPC5 activator rescued morphine‐induced downregulation of [Ca^2+^] _i_ (Figure 5E). However, neither saline group nor morphine group displayed an increased Ca^2+^ influx after AC1903 treatment in kreb's buffer containing L‐type calcium channel inhibitor and N‐type calcium channel inhibitor (Figure 5F,G; Figure S14A–H, Supporting Information). Additionally, extracellular Ca^2+^ deletion with EGTA (2 mm, 2 h) and intracellular Ca^2+^ deletion with BAPTA‐AM (10 µM, 2 h) abolished RLZ‐induced GABA release in primary spinal neurons (Figure S14I, Supporting Information). These findings indicate that TRPC5‐mediated Ca^2+^ influx is crucial for GABA release, and that TRPC5 activation can counteract morphine‐induced suppression of Ca^2+^ influx and subsequent GABA release.

To detect the dynamic Ca^2+^ signals following chronic morphine exposure, *ex vivo* calcium imaging was conducted in the superficial dorsal horn of mice on days 3, 5, and 7 after intrathecal morphine injection. To enable this, mice were intrathecally injected with pAAV‐*Gad67*‐mCherry‐WPRE and pAAV‐*hSyn*‐jGCaMP7f‐WPRE vectors 21 days prior to the initiation of tolerance‐inducing morphine treatment (Figure 5H). Data revealed a significant reduction in calcium levels within GABAergic inhibitory interneurons on days 3, 5, and 7 following intrathecal morphine administration (Figure 5I; Figure S15A, Supporting Information). Next, we performed *ex vivo* calcium imaging in the superficial dorsal horn of *Trpc5* ^−/−^ and wild type mice. Representative fluorescence images showed that *Trpc5* deficiency resulted in reduced calcium concertation in GABAergic interneurons (Figure 5J; Figure S15B, Supporting Information).

### 2.3 Morphine‐Induced Trimethylation of H3K27 at *Trpc5* Promoter Contributes to *Trpc5* Suppression

**Figure 6:**
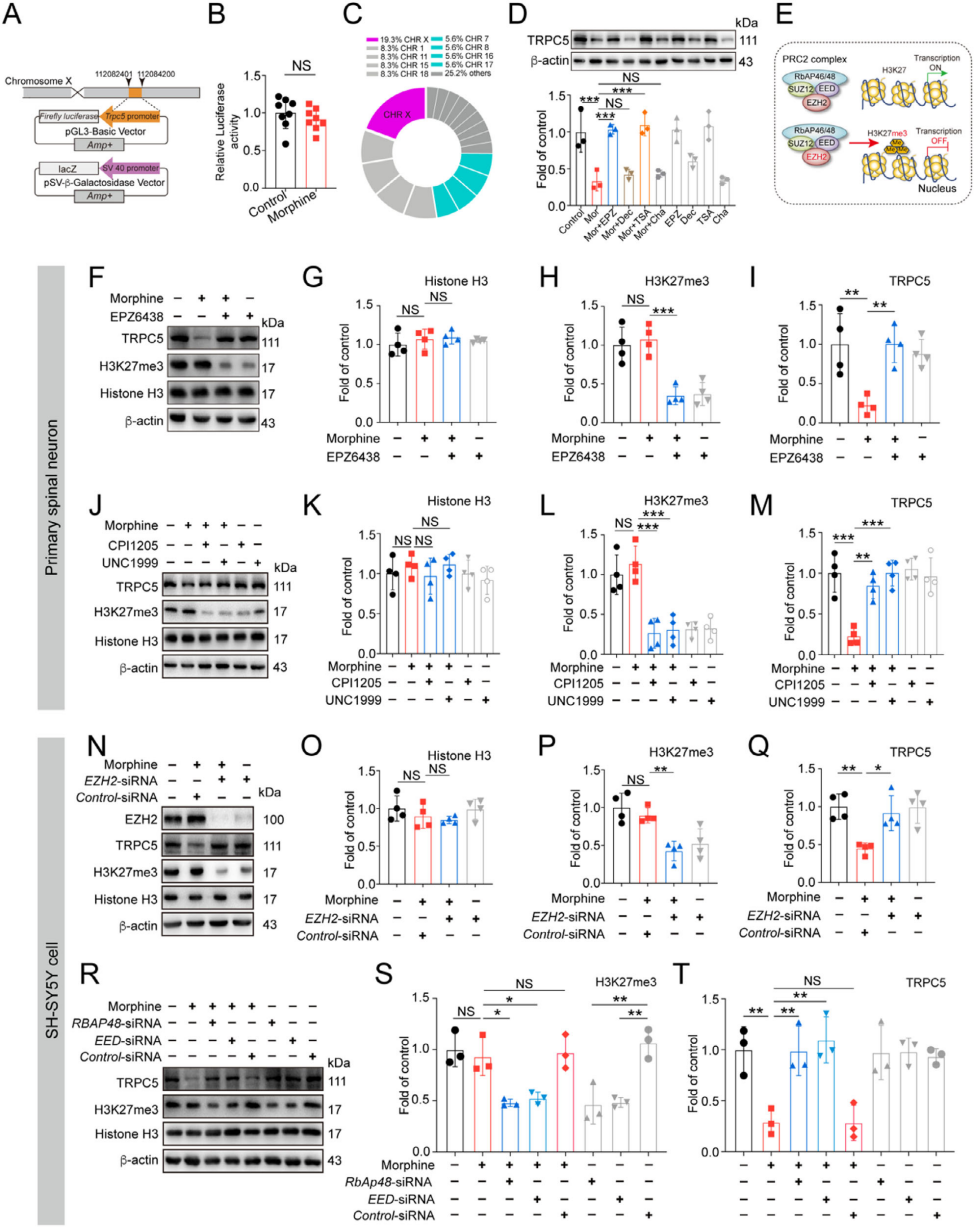
EZH2 mediates the transcriptional suppression of *Trpc5* induced by morphine. A) The schematic representation of plasmid construction for *luciferase* reporter assay. SH‐SY5Y cells were transfected with *pGL3‐basic‐TRPC5*‐*luc* vector or *pSV‐β‐galactosidase* vector and then exposed to morphine (200 µM, 14 h). B) The activity of *TRPC5* promoter was evaluated by Fluc expression, while the β‐gal signal was an internal control (Unpaired Student's *t* test, *t* (10) = 0.1437, *P* = 0.8886, *n* = 6). C) Donut chart outlines the percentage of down‐regulated differentially expressed genes in different chromosomes. D) Primary spinal neurons were pretreated with HDAC inhibitor (Trichostatin A, TSA, 100 nM), H3K9 methyltransferase inhibitor (Chaetocin, Cha, 100 nM), DNMT inhibitor (Decitabine, Dec, 5 µM) and the H3K27me3 inhibitor (EPZ6438, EPZ, 1 µM) respectively for 72 h and then subjected to morphine (200 µM, 14 h). The samples were collected for immunoblotting. EPZ6438 and Trichostatin A abolished the suppressive effect of morphine on TRPC5 expression (one‐way ANOVA, *F* (9, 20) = 16.94, *P* < 0.0001, *n* = 3). E) Schematic diagram of PRC2 complex‐mediated transcriptional regulation. F–I) Primary spinal neurons were pretreated with EPZ6438 (1 µM, 72 h) and then subjected to morphine (200 µM, 14 h). The samples were collected for immunoblotting analysis. EPZ6438 decreased the H3K27me3 levels and reversed morphine‐induced the downregulation of TRPC5 protein levels (Histone H3: one‐way ANOVA, *F* (3, 12) = 0.5530, *P* = 0.6558, *n* = 4; H3K27me3: one‐way ANOVA, *F* (3, 12) = 19.82, *P* < 0.0001, *n* = 4; TRPC5: one‐way ANOVA, *F* (3, 12) = 8.715, *P* < 0.0024, *n* = 4). J–M) Primary spinal neurons were pretreated with CPI1205 (1 µM, 72 h) and UNC1999 (1 µM, 72 h) respectively and subsequently exposed to morphine (200 µM, 14 h). The samples were collected for the protein detection. Both CPI1205 and UNC1999 inhibited the levels of H3K27me3 and abolished the effect of morphine on the TRPC5 (Histone H3: one‐way ANOVA, *F* (5, 18) _=_ 18.65, *P* < 0.0001, *n* = 4; H3K27me3: one‐way ANOVA, *F* (5, 18) = 0.7377, *P* = 0.6050, *n* = 4; TRPC5: one‐way ANOVA, *F* (5, 18) = 12.76, *P* < 0.0001, *n* = 4). N–Q) SH‐SY5Y cells transfected with *EZH2* siRNA or *control* siRNA were subjected to morphine (200 µM, 14 h). Knockdown efficacy was examined by immunoblotting. The H3K27me3 levels were decreased after EZH2 inhibition (Histone H3: one‐way ANOVA, *F* (3, 12) = 1.188, *P* = 0.3556, *n* = 4; H3K27me3: one‐way ANOVA, *F* (3, 12) = 12.23, *P* = 0.0006, *n* = 4). Knockdown of *EZH2* abolished the inhibitory effect of morphine on TRPC5 protein levels (TRPC5: one‐way ANOVA, *F* (3, 12) =8.423, *P* = 0.0028, *n* = 4). R,T) SH‐SY5Y cells were transfected with *RbAp48* siRNA, *EED* siRNA and *control* siRNA for 72 h and then subjected to morphine (200 µM, 14 h). The samples were collected for immunoblotting. Knockdown of *RbAp48* and *EED* restored the expression of TRPC5 to baseline via inhibiting H3K27me3 (H3K27me3: one‐way ANOVA, *F* (7, 16) = 11.56, *P* < 0.0001, *n* = 4, TRPC5: one‐way ANOVA, *F* (7, 16) = 8.731, *P* = 0.0002, *n* = 3). Data are expressed as mean ± SD, *P < 0.05, **P < 0.01, and ***P < 0.001.

Despite establishing that morphine inhibits *Trpc5* transcription in spinal GABAergic interneurons, the mechanisms underlying *Trpc5* suppression caused by morphine remained largely unknown. Based on transcription factor binding predictions provided by QIAGEN and cited in the public database GeneCards, potential transcription factors that may bind to the human *TRPC5* promoter include CBF (2), CBF‐A, CBF‐B, CP1A, HNF‐3b, NF‐Y, NF‐YA, NF‐YB, RREB‐1, and Sp1. Accordingly, we performed a luciferase assay to investigate whether alterations in these transcription factors drove *Trpc5* transcriptional suppression. The full length of human *TRPC5* promoter was cloned into the pGL3‐basic vector to generate a human *TRPC5*‐luc reporter construct (**Figure** **6**A). SH‐SY5Y cells were transfected with this reporter plasmid along with a *pSV*‐β‐galactosidase vector and then treated with morphine for 14 h. The reporter luciferase assay showed that morphine did not affect *TRPC5* promoter activity (Figure 6B), suggesting that morphine‐induced *TRPC5* transcriptional suppression is not due to alterations in these transcription factors. Next, we aim to elucidate the molecular mechanisms underlying the transcriptional repression of *Trpc5*. It has been reported that the transcriptional activity of a gene can be regulated by its promoter, and that promoter methylation, together with interactions with various histone modifications, can profoundly influence gene expression.^[^ ^32^, ^33^ ^]^ The molecular mechanisms associated with the regulation of promoter function have been extensively documented in the field of epigenetics. The functional connection between DNA methylation and transcriptional repression is well‐established.^[^ ^34^, ^35^ ^]^ However, the relationship between histone modifications and transcriptional regulation is more complex. Generally, increased histone acetylation promotes the remodeling of chromatin from a compact structure to a more relaxed conformation, facilitating transcriptional activation.^[^ ^36^ ^]^ In contrast, histone methylation can mediate transcriptional repression, as demonstrated by the dimethylation of histone H3 lysine 9 (H3K9me2), trimethylation of histone H3 lysine 9 (H3K9me3), and trimethylation of histone H3 lysine 27 (H3K27me3), all of which contribute to transcriptional repression.^[^ ^33^ ^]^ Our RNA‐seq analysis revealed that differentially expressed genes in morphine‐tolerant mice were primarily located on chromosome X, including *Trpc5* (Figure 6C). Building on this background, we hypothesize that epigenetic regulation might contribute to the morphine‐induced transcriptional repression of *Trpc5* and have designed a series of experiments to test this hypothesis.

To investigate whether DNA methylation and histone modifications were involved in morphine‐induced suppression of *Trpc5*, we employed several inhibitors: the enhancer of zeste homolog 2 (EZH2) inhibitor EPZ6438, the DNA methyltransferase (DNMT) inhibitor Decitabine, the class I/II histone deacetylase (HDAC) inhibitor Trichostatin A (TSA), and the histone H3K9 methyltransferase inhibitor chaetocin. Primary spinal neurons were treated with TSA (100 nM), Chaetocin (Cha, 100 nM), Decitabine (Dec, 5 µM) and H3K27 trimethyltransferase (EZH2) inhibitor (EPZ6438, EPZ, 1 µM) to assess their roles in *Trpc5* transcriptional regulation under morphine treatment. Quantitative PCR and western blot data showed that EPZ6438 and TSA reversed morphine‐induced downregulation of TRPC5 (Figure 6D; Figure S16, Supporting Information). EZH2 is the enzymatically active core subunit of polycomb repressive complex 2 (PRC2), which also contains embryonic ectoderm development (EED), suppressor of zeste 12 (SUZ12), and retinoblastoma (Rb)‐associated proteins 46/48 (RbAp46/48). EZH2 is primarily known for its essential role in regulating epigenetic signatures, particularly through the trimethylation of histone 3 at lysine 27 (H3K27me3), which contributes to transcriptional silencing (Figure 6E). As shown in Figure 6F–I, EPZ6438 treatment decreased H3K27me3 levels and increased TRPC5 expression compared with the morphine‐treated group in primary spinal neurons. Consistently, two other EZH2 inhibitors, CPI1205 and UNC1999, also effectively countered morphine‐induced TRPC5 inhibition (Figure 6J–M). To further explore the role of EZH2 in morphine‐induced TRPC5 suppression, SH‐SY5Y cells were transfected with *EZH2* siRNA followed by morphine treatment. We found that *EZH2* siRNA significantly reduced EZH2 and H3K27me3 levels and reversed morphine‐induced TRPC5 suppression (Figure 6N–Q). To investigate whether morphine‐induced TRPC5 downregulation was PRC2‐dependent, SH‐SY5Y cells were transfected with *EED* siRNA or *RbAp48* siRNA and then exposed to morphine. Transfection efficiency was confirmed by immunoblotting (Figure S17A,B, Supporting Information). As shown in Figure 6R–T, silencing of *EED* or *RbAp48* effectively reversed morphine‐induced TRPC5 inhibition.

**Figure 7:**
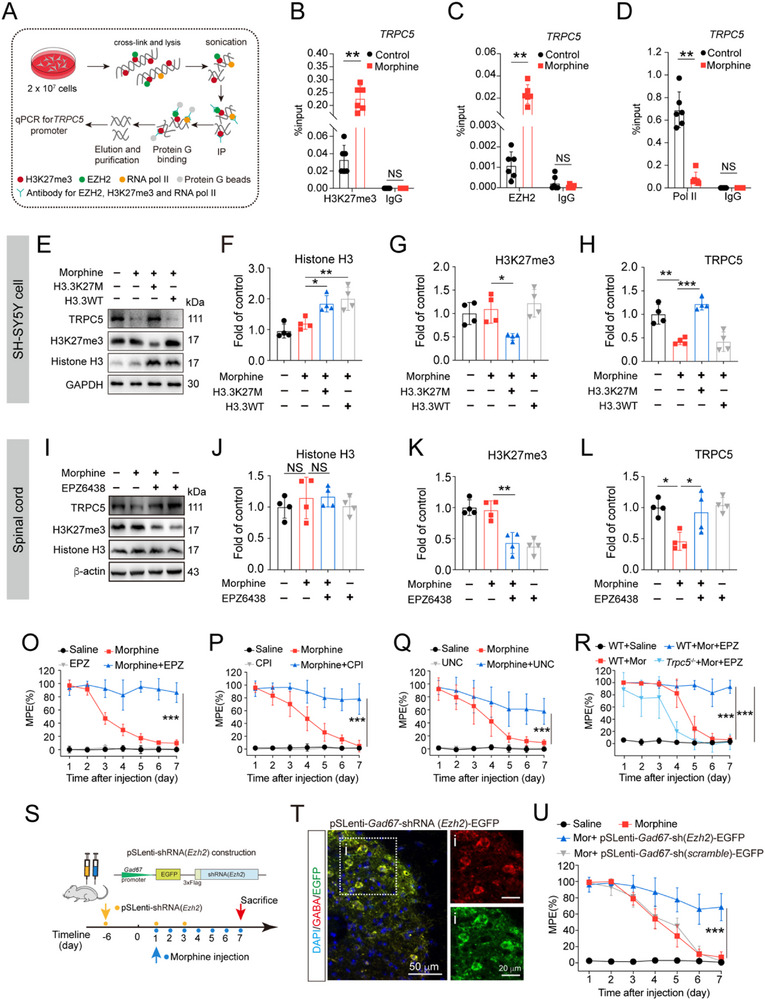
Morphine increases H3K27me3 at the TRPC5 promoter and blockage of EZH2 in GABAergic interneurons reverses morphine tolerance. A–D) SH‐SY5Y cells were subjected to morphine (200 µM, 14 h) and the samples were collected for qChIP. Primer information was provided in Table S6 (Supporting Information). Binding of H3K27me3 B) and EZH2 C) to *TRPC5* promoter were increased in response to morphine. The total of Pol II binding to *TRPC5* promoter was decreased after morphine exposure D) (EZH2: Unpaired Student's *t* test, *t* (10) = 6.521, *P* < 0.0001, *n* = 6; H3K27me3: Unpaired Student's *t* test, *t* (10) = 9.434, *P* < 0.0001, *n* = 6; Pol II: Unpaired Student's *t* test, *t* (10) = 8.896, *P* < 0.0001, *n* = 6). E–H) SH‐SY5Y cells were transfected with H3.3‐K27M mutation or H3.3WT vectors for 72 h and then subsequently exposed to morphine (200 µM, 14 h). The samples were collected for the protein detection. H3.3‐K27M mutation or H3.3WT vectors overexpression enhanced the levels of histone H3. H3.3‐K27M mutation overexpression reversed morphine‐induced the downregulation of TRPC5 via decreasing the levels H3K27me3. (Histone H3: one‐way ANOVA, *F* (3, 12) = 13.35, *P* = 0.0004, *n* = 4; H3K27me3: one‐way ANOVA, *F* (3, 12) = 6.643, *P* = 0.0068, *n* = 4; TRPC5: one‐way ANOVA, *F* (3, 12) = 24.66, *P* < 0.0001, *n* = 4). I–L) EZP6438 abolished chronic morphine‐induced the downregulation of TRPC5 in spinal cords L4‐L5 via suppression of H3K27me3. EZP6438 abolished chronic morphine‐induced the downregulation of TRPC5 in spinal cords L4‐L5 via suppression of H3K27me3 (Histone H3: one‐way ANOVA, *F* (3, 12) = 0.6320, *P* = 0.6084, *n* = 4; H3K27me3: one‐way ANOVA, *F* (3, 12) = 22.08, *P* < 0.0001, *n* = 4; TRPC5: one‐way ANOVA, *F* (3, 12) = 7.173, *P* = 0.0051, *n* = 4). O) Intrathecal injection with EPZ6438 (2 µg/10 µL) prevented the development of morphine tolerance (two‐way ANOVA, drug effect: *F* (3, 196) = 1460, *P* < 0.0001; time effect: *F* (6, 196) = 44.19, *P* < 0.0001; drug × time effect: *F* (18, 196) = 37.85, *P* < 0.0001, *n* = 8). P,Q) Intrathecal injection with CPI1205 (CPI, 2 µg/10 µL) P) and UNC1999 (UNC, 2 µg/10 µL) Q) respectively also prevented morphine tolerance (UNC1999: drug effect: *F* (3, 196) = 576.4, *P* < 0.0001; time effect: *F* (6, 196) = 35.45, *P* < 0.0001; drug × time effect: *F* (18, 196) = 16.35, *P* < 0.0001, *n* = 8; CPI1205: drug effect: *F* (3, 308) = 1226, *P* < 0.0001; time effect: *F* (6, 308) = 47.90, *P* < 0.0001; drug × time effect: *F* (18, 308) = 29.68, *P* < 0.0001, *n* = 12). R) *Trpc5* deficiency abolished the effect of EPZ6438 on morphine tolerance (two‐way ANOVA, drug effect: *F* (3, 140) = 307.4, *P* < 0.0001; time effect: *F* (6, 140) = 57.22, *P* < 0.0001; drug × time effect: *F* (18, 140) = 16.59, *P* < 0.0001, *n* = 6 mice). S) Schematic diagram illustrated the regimen of knockdown of *EZH*2 in GABAergic interneurons during morphine tolerance in mice. Mice were intrathecally injected with pSLenti‐shRNA (*Ezh2*) or its control vector before chronic morphine exposure (10 µg/10 µL, 7 d). T) Representative photomicrographs with an inset showed pSLenti‐mediated *EZH2* knockdown (EGFP^+^, green) in GABAergic interneurons (GABA, red) in the spinal cord of mice (*n* = 3). U) Knockdown of *EZH2* in GABAergic interneurons of spinal cord significantly improved chronic morphine tolerance (two‐way ANOVA, drug effect: *F* (3, 252) = 726.8, *P* < 0.0001; time effect: *F* (6, 252) = 34.27, *P* < 0.0001, drug × time effect: *F* (18, 252) = 34.27, *P* < 0.0001, *n* = 10). Data are expressed as mean ± SD, *P < 0.05, **P < 0.01, and ***P < 0.001.

It is notable that morphine did not affect H3K27me3 protein levels either in vitro (Figure 6F,H,J,L) or in vivo (Figure S18, Supporting Information). To explore the mechanisms underlying the effect of EZH2 on *Trpc5* transcriptional suppression caused by morphine, quantitative chromatin immunoprecipitation (qChIP) was performed using antibodies against H3K27me3, EZH2 and Pol II (**Figure** **7**A). Our results showed that morphine exposure (200 µM, 14 h) increased the binding of H3K27me3 to the *TRPC5* promoter in SH‐SY5Y cells compared with controls (Figure 7B). Following this, we assessed the binding of the H3K27 methylation‐modifying enzyme EZH2 to the *TRPC5* promoter. Consistently, morphine also enhanced EZH2 binding to the *TRPC5* promoter (Figure 7C). Additionally, we observed that morphine treatment reduced the binding of total Pol II to the *TRPC5* promoter (Figure 7D), which may be associated with suppression of *TRPC5* transcription induced by morphine.

To directly test the role of H3K27me3 in morphine‐induced *Trpc5* suppression, we generated a histone H3 variant encoded by *H3F3A* gene mutation, which results in the substitution of lysine 27 with methionine (H3K27M). Overexpression of H3K27M in SH‐SY5Y cells increased total H3 levels and decreased H3K27me3 expression (Figure 7E–G). The effect of H3K27M on morphine‐induced TRPC5 suppression was found to be highly consistent with the effects of EZH2 inhibitors (Figure 7H). To investigate the impact of EZH2 deficiency on TRPC5 expression in the spinal cord, mice were intrathecally co‐administrated with morphine (10 µg/10 µL) and EZH2 inhibitor EPZ6438 (2 µg/10 µL) for 7 consecutive days. After 7 days of morphine administration, we observed a significant decrease in TRPC5 protein levels in spinal L4‐L5 sections. EPZ6438 effectively reversed morphine‐induced TRPC5 inhibition by downregulating H3K27me3 (Figure 7I–L). Additionally, EPZ6438 nearly restored morphine analgesia, with the MPE increased from 9.9% to 86.4% on day 7 (Figure 7O). Two other EZH2 inhibitors similarly restored morphine analgesia (Figure 7P,Q). To further determine whether the effect of the EZH2 inhibitor on morphine tolerance depended on TRPC5, we used *Trpc5* ^−/−^ mice in the tail‐flick test. The results showed that *Trpc5* deficiency abolished the improvement of morphine tolerance by EPZ6438 (Figure 7R). TRPC5 antagonist AC1903 similarly negated the effect of EPZ6438 on morphine tolerance (Figure S19A). Furthermore, we constructed a lentivirus vector to knock down *Ezh2* specifically in spinal GABAergic interneurons (Figure 7S; Figure S19B, Supporting Information). Western blot data showed that EZH2 inhibition abolished morphine‐induced the downregulation of TRPC5 (Figure S19C, Supporting Information). Behavioral analysis demonstrated that *Ezh2* deficiency in spinal GABAergic interneurons significantly enhanced the analgesic effect of morphine compared with scrambled controls (Figure 7T,U). Collectively, these data demonstrates that EZH2‐mediated *Trpc5* transcriptional suppression plays a critical role in morphine tolerance.

**Figure 8:**
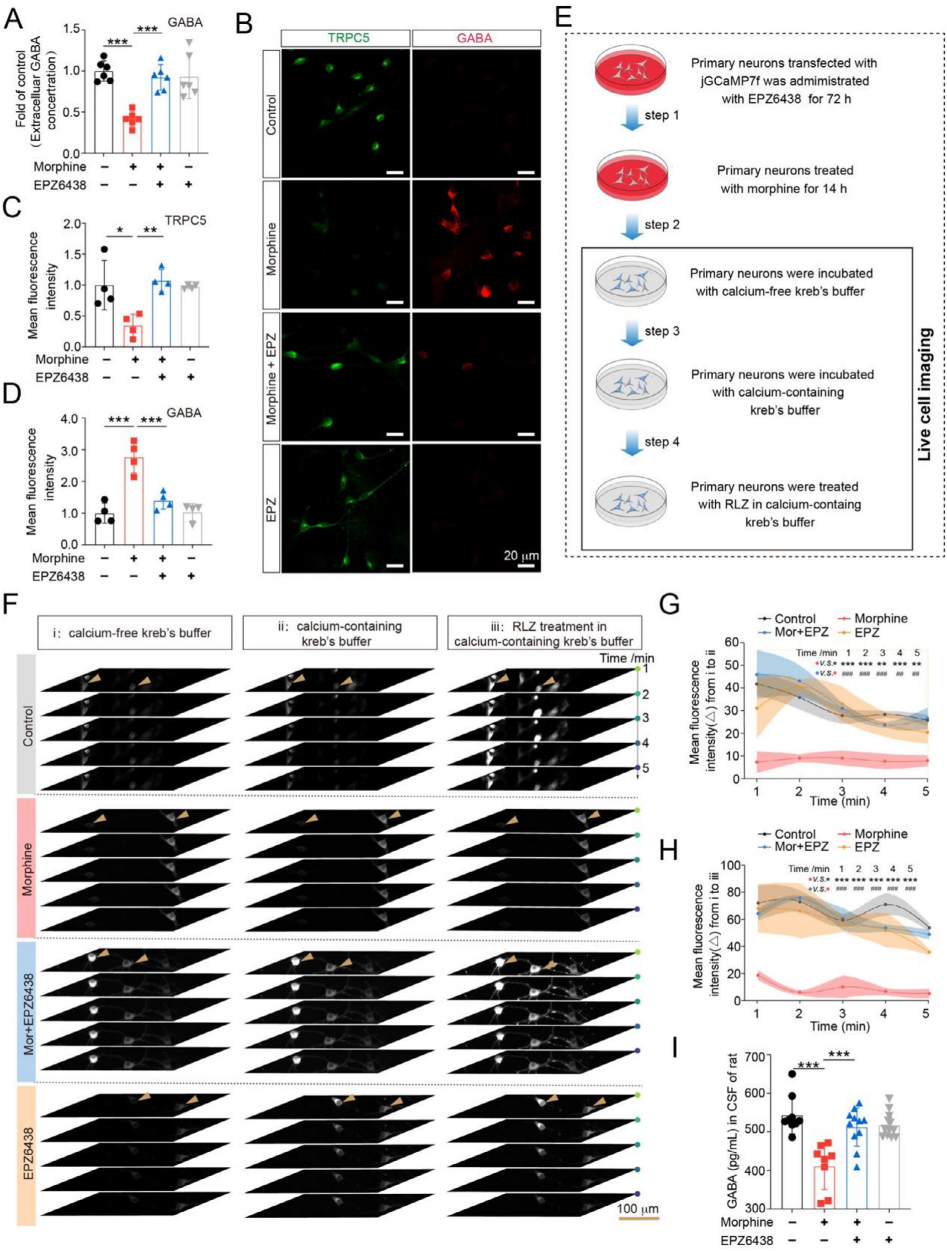
Inhibiting EZH2 improves morphine‐induced disruption of Ca^2+^ homeostasis and restores GABA release. A–D) Primary spinal neurons were pretreated with EPZ6438 (1 µM, 72 h) and subsequently subjected to morphine (200 µM, 14 h). The supernatants were collected for GABA detection. A) Representative images of immunofluorescence showed the intracellular levels of GABA and TRPC5 expression after the treatment of morphine and EPZ6438 (*n* = 4). B) Fluorescence quantitative analysis showed that EPZ6438 reversed morphine‐induced the downregulation of TRPC5 expression (one‐way ANOVA, *F* (3, 12) = 51.56, *P* < 0.0001). C) Fluorescence quantitative analysis displayed that EPZ6438 abolished morphine‐induced decrease of GABA release (one‐way ANOVA, *F* (3, 12) = 52.28, *P* < 0.0001). D) ELISA analysis of GABA in collected supernatants showed that EPZ6438 reversed the decreased release of GABA caused by morphine (one‐way ANOVA, *F* (3, 20) = 32.3, *P* < 0.0001). E) Schematic diagram displayed the process of calcium imaging in primary spinal neurons. F) Primary spinal neurons were transfected with calcium indicator pSLenti‐*Gad67*‐jGCaMP7f‐Puro‐WPRE and exposed to EPZ6438 (1 µM, 72 h). Calcium imaging of primary spinal neurons displayed Ca^2+^ influx from 1 to 5 min. Representative images showed (i) neurons were incubated with calcium‐free kreb's buffer; (ii) calcium‐containing kreb's buffer; (iii) RLZ in calcium‐containing kreb's buffer. G) Fluorescence quantitative analysis showed that EZP6438 reversed Ca^2+^ influx decrease caused by morphine after the incubation with calcium‐containing kreb's buffer (two‐way ANOVA, drug effect: *F* (3,40) = 69.45, *P* < 0.0001; time effect: *F* (4, 40) = 12.30, *P* < 0.0001; drug × time effect: *F* (12, 40) = 2.497, *P* = 0.0150, *n* = 3; **P < 0.01, ***P < 0.001, morphine group *vs*. control group; ^##^ *P <* 0.01, ^###^ *P* < 0.001, Mor + EPZ group *vs*. EPZ group). H) After the treatment of RLZ, EPZ6438 improved Ca^2+^ influx deficiency caused by morphine (two‐way ANOVA, drug effect: *F* (3, 40) = 114.2, *P* < 0.0001; time effect: *F* (4, 40) = 8.840, *P* < 0.0001; drug × time effect: *F* (12, 40) = 1.328, *P* = 0.2417, *n* = 3; ***P < 0.001, morphine group *v.s*. control group; ^###^ *P* < 0.001, Mor + EPZ group *vs*. EPZ group). I) ELISA analysis of GABA in CSF from rats exposed to morphine (i.t., 20 εg/10 εL) and treated with EPZ6438 (i.t., 4 µg/10 µL) for 7 continuous days (one‐way ANOVA, *F* (3, 36) = 12.66, *P* < 0.0001, control group: *n* = 9, morphine group: *n* = 8, morphine paired with EPZ group: *n* = 11, EPZ group: *n* = 12). Data are expressed as mean ± SD, *P < 0.05, **P < 0.01 and ***P < 0.001.

Of note, EPZ6438 did not alter GAD67 expression levels in primary spinal neurons (Figure S9C, Supporting Information), indicating that EZH2 inhibition had no effect on GABA synthesis. To further investigate whether EZH2 inhibition could mitigate morphine‐induced suppression of GABA release, primary spinal neurons were pretreated with EPZ6438 (1 µM, 72 h), followed by exposure to morphine (200 µM) in the presence of EPZ6438 for 14 h. ELISA detection was performed to measure the extracellular GABA levels. As shown in **Figure** **8**A, EPZ6438 reversed the morphine‐induced decrease in GABA release. Additionally, confocal imaging showed that morphine induced a significant downregulation of TRPC5 (green) and an increase in intracellular GABA accumulation (red), whereas EPZ6438 effectively restored TRPC5 expression and reduced GABA accumulation (Figure 8B–D). To assess the effect of EPZ6438 on morphine‐induced decreases in Ca^2+^ influx, we utilized the calcium indicator jGCaMP7f for calcium imaging. Primary spinal neurons were transfected with pSLenti‐*Gad67*‐jGCaMP7f‐Puro‐WPRE 24 h before EPZ6438 treatment (1 µM, 72 h). After EPZ6438 treatment, neurons were exposed to morphine for 14 h. The medium was then replaced with kreb's buffer containing L‐type calcium channel inhibitor (Nifedipine, 1 µM) and N‐type calcium channel inhibitor (Cav 2.2 blocker 1, 10 µM) (Figure 8E). Data showed that EPZ6438 abolished the morphine‐induced reduction in Ca^2+^ influx, with a significantly higher increase in Ca^2+^ influx observed in the EPZ6438 treatment group compared with the morphine‐treated group (Figure 8F–H; Figure S20, Supporting Information). Furthermore, rats were intrathecally co‐administered with morphine (20 µg/10 µL) and EPZ6438 (4 µg/10 µL) for 7 consecutive days and CSF were collected at the end of the treatment period. ELISA results showed that EPZ6438 effectively restored GABA release reduced by morphine (Figure 8I).

## 3 Discussion

**Figure 9:**
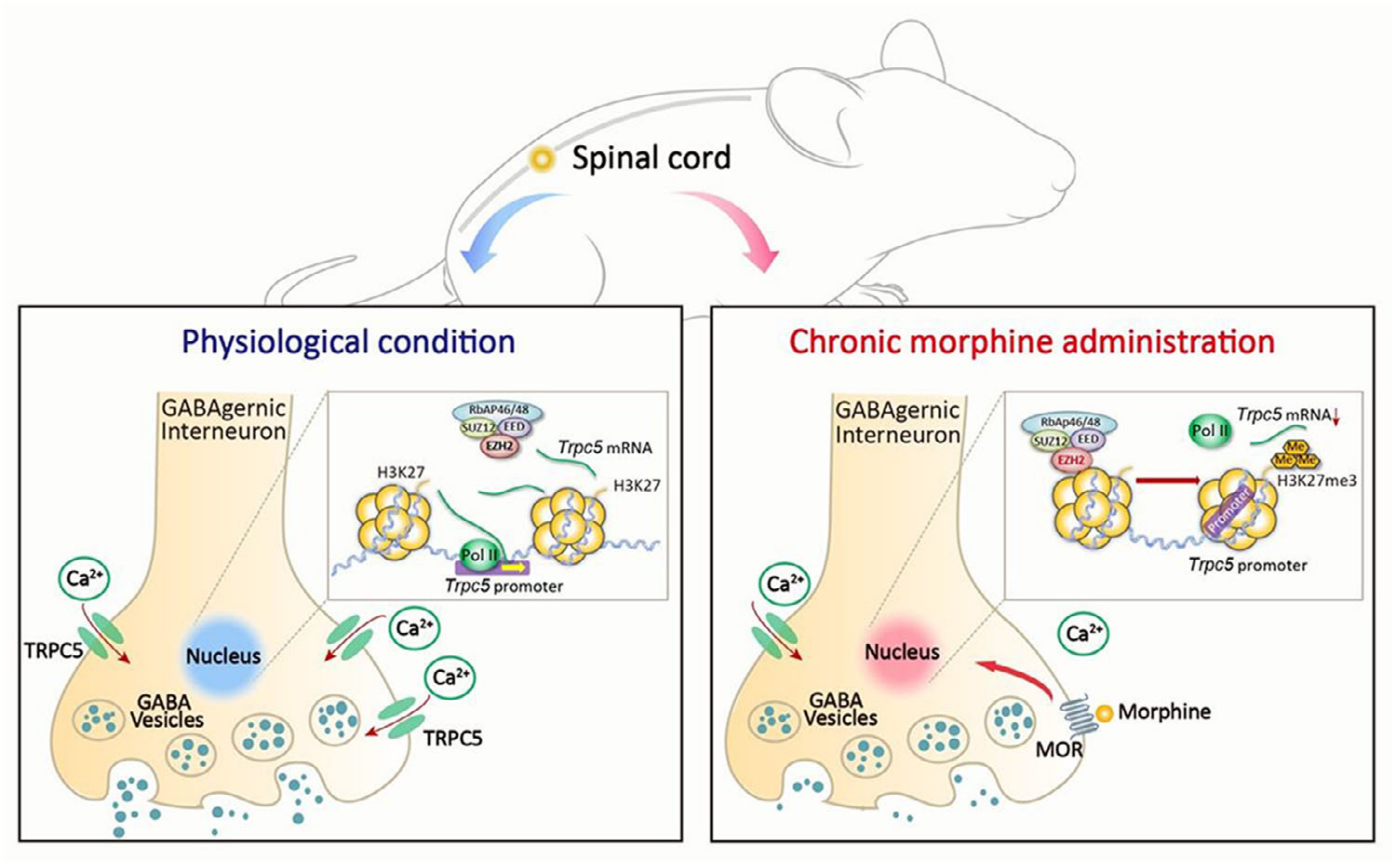
Schematic model of the mechanisms underlying transcriptional suppression of *Trpc5* in GABAergic interneurons by chronic intrathecal morphine injection.

Our findings suggest that chronic morphine induces the transcriptional suppression of *Trpc5* in GABAergic interneurons, which in turn reduces GABA release by inhibiting Ca^2+^ influx in spinal lamina I‐III. Activation of TRPC5 in spinal GABAergic interneurons significantly enhances the analgesic effect of morphine. Using comprehensive epigenetic analyses, we found that morphine exposure induces histone modification at the *Trpc5* promoter mediated by EZH2. Specifically, morphine increases the binding of EZH2 and H3K27me3 to the *Trpc5* promoter, leading to a reduction in Pol II binding at the same site. Furthermore, intrathecal administration of EZH2 inhibitors effectively reverses morphine tolerance. These findings highlight the significant translational potential of spinal TRPC5 and EZH2 as therapeutic targets for alleviating morphine tolerance (**Figure** **9**).

Transient receptor potential (TRP) channels are critical cellular sensors in sensory physiology and classified into six subfamilies: TRPC for “canonical”, TRPV “vanilloid”, TRPM “melastatin”, TRPP for “polycystin”, TRPML for “mucolipin”, and TRPA for “ankyrin”.^[^ ^37^ ^]^ Early drug discovery efforts targeting TRP channels focused primarily on pain.^[^ ^38^, ^39^ ^]^ TRPV1, a heat‐sensitive channel, is upregulated by chronic morphine exposure, which contributes to tolerance‐associated thermal hyperalgesia.^[^ ^40^ ^]^ TRPA1, expressed in central nociceptive terminals, mediates cold pain, and its inhibition prevents morphine‐induced antinociceptive tolerance.^[^ ^41^ ^]^ TRPC5, a member of the “canonical” TRP (TRPC) subfamily, is a non‐selective cation channel that mediates Ca^2+^ influx.^[^ ^42^ ^]^ Structurally and functionally, TRPC channels are organized into heteromultimeric complexes, including the TRPC1/4/5 and TRPC3/6/7 subgroups. TRPC1, TRPC4, and TRPC5 are co‐expressed in the brain. Recent studies have identified TRPC5, which is also expressed in kidney, as a promising therapeutic target for protecting against focal segmental glomerulosclerosis (FSGS) through selective inhibition.^[^ ^24^ ^]^ Sadler et al. demonstrated that pharmacological inhibition via peripheral AC1903 administration and genetic deletion of *Trpc5* could prevent persistent mechanical hypersensitivity in inflammatory pain models induced by CFA injection.^[^ ^26^ ^]^ This pain condition was associated with elevated lysophosphatidylcholine (LPC) in the DRG, which acts as an endogenous agonist for TRPC5. Notably, Chu et al. reported that *Trpc1/4/5* triple knockout mice exhibited reduced morphine tolerance compared with WT mice in a thermal nociception assay.^[^ ^43^ ^]^ In this study, the authors evaluated the expression profiles of TRPC1, TRPC4, and TRPC5 at both the transcriptional and protein levels in the spinal cord of mice following subcutaneous morphine administration. Their data revealed that only TRPC4 exhibited significant changes in both mRNA and protein expression, suggesting that TRPC4, rather than TRPC1 or TRPC5, may contribute to the development of morphine tolerance. However, this study did not investigate the cellular distribution profile of TRPC4. Additionally, the authors employed the AAV2/9‐CaMKII‐GCaMP6s viral construct, administered intrathecally to WT and *Trpc1/4/5* triple knockout mice, to examine calcium dynamics in excitatory neurons following DAMGO stimulation. This approach implies that the authors hypothesized TRPC4 to be functionally active within excitatory neurons. The calcium imaging data showed that bath application of DAMGO (500 nM, 10 min) induced a Ca^2+^ elevation, which was attenuated in *Trpc1/4/5* triple KO mice. It is unlikely that substantial changes in TRPC4 expression would occur within the brief timeframe of 10 min. These findings suggest that the critical role of TRPC4 in excitatory neurons may not rely on changes in its expression level during the development of subcutaneous morphine‐induced tolerance. Consistent with the findings of Chu et al., our results showed that subcutaneous morphine administration did not alter TRPC5 expression in the spinal cord (data not shown). Taken together, our study and the published findings by Chu et al., suggest that subcutaneous morphine injection increases TRPC4 protein expression in spinal excitatory neurons and enhances their excitability, thereby contributing to the development of morphine tolerance. In contrast, our findings demonstrate that chronic intrathecal morphine administration suppresses *Trpc5* transcription in spinal GABAergic interneurons, thereby reducing GABA release through the inhibition of Ca^2+^ influx. Activation of TRPC5 in spinal GABAergic interneurons significantly enhances the analgesic effect of morphine.

Nociceptive inputs from Aδ and C primary afferent fibers can be modulated at the spinal cord level through the activation of GABAergic inhibitory interneurons, in accordance with the gate‐control theory of pain.^[^ ^44^ ^]^ As a critical inhibitory neurotransmitter in the mammalian CNS, GABA release is regulated in a calcium‐dependent manner.^[^ ^45^ ^]^ We found that spinal activation of TRPC5 by riluzole (RLZ) significantly promoted GABA release. *Ex vivo* two‐photon calcium imaging revealed that TRPC5 deficiency markedly reduced calcium concentration in GABAergic interneurons, resulting in decreased GABA release. These data strongly suggest that TRPC5 plays a key role in maintaining GABA release in the spinal cord. Long‐term intrathecal morphine injection notably suppressed TRPC5 expression in the spinal dorsal horn of mice. We demonstrated that both pharmacological activation and genetic overexpression of TRPC5 in the spinal cord significantly attenuated morphine tolerance. Riluzole (RLZ) was originally approved by the FDA for the treatment of amyotrophic lateral sclerosis (ALS) and is known to reduce presynaptic glutamate release, partly through inactivation of voltage‐gated sodium (Na_v_) channels and noncompetitive inhibition of NMDA receptors. Nine Na_v_ isoforms (Na_v_1.1‐Na_v_1.9) are differentially expressed in excitable cells.^[^ ^46^ ^]^ While these subtypes share a conserved structural framework, they differ in amino acid sequence, biophysical properties, tissue‐ and development‐specific expression, and physiological function.^[^ ^47^ ^]^ Notably, Na_v_1.7, Na_v_ 1.8, and Na_v_ 1.9 are abundantly expressed in the peripheral nervous system (PNS), particularly in dorsal root ganglion and trigeminal ganglion sensory neurons that convey pain signals to the central nervous system (CNS).^[^ ^48^ ^]^ In the study by Huang et al., Na_v_ 1.7 was found to adopt an inactivated state characterized by a closed intracellular gate in the presence of RLZ.^[^ ^49^ ^]^ In our study, to minimize potential effects on Na_v_ channels in the PNS, we administered RLZ intrathecally, thereby delivering the drug directly to the spinal cord. Behavioral analysis showed that RLZ alone did not elicit significant analgesic effects, whereas both RLZ and the selective TRPC5 agonist BTD significantly attenuated morphine tolerance. Importantly, no studies to date have reported effects of BTD on Na_v_ channel activity. Collectively, these findings suggest that RLZ mitigates morphine tolerance primarily through TRPC5‐mediated regulation of GABA release, rather than inhibition of Na_v_ channels or suppression of glutamate release.

Notably, spinal activation of TRPC5 by RLZ restored cerebrospinal fluid (CSF) GABA levels to baseline in morphine‐tolerant rats but only partially prevented the development of tolerance, whereas intrathecal administration of the GABA_A_ receptor agonist etomidate proved more effective. GABA exerts its effects through binding to GABA receptors, primarily GABA_A_ and GABA_B_ receptors. Pejo et al. demonstrated that both etomidate and GABA share comparable potencies at GABA_A_ receptors, with EC_50_ values of ≈1.8 µM and > 90% receptor activation at 100 µM.^[^ ^50^, ^51^ ^]^ Under physiological conditions, CSF GABA concentrations typically range from ≈90 nM to 3 µM, values that fall well below or approach the EC_50_.^[^ ^52^, ^53^ ^]^ In our study, intrathecal administration of etomidate was estimated to achieve CSF concentrations of ≈300 µM‐ far exceeding the threshold required for maximal receptor activation. Overall, TRPC5 agonists, including RLZ and BTD, partially attenuate morphine tolerance, whereas etomidate exerts a stronger effect, likely through robust and near‐maximal activation of GABA_A_ receptor. Additionally, under certain pathological conditions, GABA may exert excitatory effects, largely due to alterations in the chloride electrochemical gradient. Ferrini et al. reported that morphine‐induced hyperalgesia downregulates KCC2, causing a collapse of the chloride gradient of spinal lamina I projection neurons and impairing the inhibitory regulation of nociceptive signaling mediated by GABAergic interneurons.^[^ ^8^ ^]^ Restoring the anion equilibrium potential reversed morphine‐induced hyperalgesia without affecting tolerance. This suggests that distinct mechanisms underlie the disinhibition observed in morphine tolerance and morphine‐induced hyperalgesia. Consistent with this, TRPC5 activation restored GABA levels and alleviated morphine tolerance, but had no effect on hyperalgesia (Figure S21, Supporting Information). Therefore, based on our current findings together with those of Ferrini et al., we propose that morphine‐induced suppression of TRPC5 leads to a reduction in GABA release. This diminished GABAergic inhibition results in inadequate suppression of excitatory neurotransmitter release from primary afferent terminals, ultimately contributing to the development of antinociceptive tolerance.

Since restoring spinal TRPC5 function represents an effective strategy for alleviating morphine tolerance, it is critical to elucidate the underlying mechanisms of morphine‐induced transcriptional suppression of *Trpc5* in the spinal cord. Using a *Trpc5* promoter‐luciferase reporter assay, we first excluded the possibility that morphine inhibits transcription factors regulating *Trpc5*. We subsequently hypothesized that morphine may directly affect the *Trpc5* promoter, thereby suppressing its transcriptional activity. It has been reported that promoter methylation, as well as its interaction with various histone modifications, can profoundly influence gene expression. Therefore, we employed several inhibitors to investigate whether epigenetic regulation is involved in morphine‐induced suppression of *Trpc5*: the H3K27me3 inhibitor EPZ6438, the DNA methyltransferase (DNMT) inhibitor Decitabine, the class I/II histone deacetylase (HDAC) inhibitor Trichostatin A, and the histone H3K9 methyltransferase inhibitor Chaetocin. To our surprise, intrarectal administration of the FDA‐approved EZH2 inhibitor, Tazemetostat (EPZ6438) effectively reversed morphine tolerance. Consistently, two additional EZH2 inhibitors, CPI1205 and UNC1999, also improved morphine tolerance. However, UNC1999 only partially alleviated morphine tolerance, whereas EPZ6438 and CPI1205 nearly completely prevented its development. EZH2 and its homolog EZH1 serve as the catalytic subunits of Polycomb Repressive Complex 2 (PRC2), mediating trimethylation of histone H3 at lysine 27 (H3K27me3). Despite their high sequence homology (76% overall; 96% within the Su(var)3‐9, Enhancer‐of‐zeste, Trithorax (SET) domain),^[^ ^54^ ^]^ EZH1 exhibits substantially lower catalytic activity compared with EZH2.^[^ ^28^, ^55^, ^56^ ^]^ Although EPZ6438, CPI1205, and UNC1999 are all potent EZH2 inhibitors, studies have shown that they also inhibit EZH1, with notable differences in selectivity between EZH2 and EZH1. For example, EPZ6438 inhibits EZH2 with an IC_50_ of 11 nM, while its IC_50_ for EZH1 is 392 nM (≈35‐fold selectivity).^[^ ^57^ ^]^ CPI1205 shows ≈24‐fold selectivity for EZH2 (IC_50_ = 2.2 nM) over EZH1 (IC_50_ = 52 nM).^[^ ^58^ ^]^ In contrast, UNC1999 displays only ≈4.5‐fold selectivity, inhibiting EZH2 (IC_50_ < 10 nM) and EZH1 (IC_50_ = 45 nM) with relatively similar potency.^[^ ^59^ ^]^ Taken together, these findings suggest that the relatively lower selectivity of UNC1999 for EZH2 likely underlies its weaker efficacy in preventing morphine tolerance compared with the more selective inhibitors EPZ6438 and CPI1205.

In recent years, increasing interest in elucidating the role of EZH2 in various biological processes has been further driven by the development of small molecules that effectively inhibit its enzymatic activity. Evidence indicates that EZH2 is implicated in the development and progression of multiple cancers, including breast cancer, bladder cancer, endometrial cancer, and prostate cancer, with elevated EZH2 levels correlating with increased tumor aggressiveness.^[^ ^60^, ^61^, ^62^ ^]^ Accordingly, EZH2 inhibitors hold considerable promise as potential cancer therapeutics. In clinical practice, morphine is the first‐line treatment for moderate to severe cancer pain, with oral or subcutaneous administration being the preferred routes.^[^ ^63^ ^]^ However, long‐term systemic exposure to morphine often leads to diminished analgesic efficacy, necessitating intrathecal administration for pain management. Despite this, morphine tolerance remains a significant challenge. Therefore, EZH2 inhibitors may offer a valuable therapeutic approach for both addressing morphine tolerance and combating various cancers. In conclusion, our findings emphasize the critical role of TRPC5 in regulating spinal GABA release and highlight the potential of EZH2 inhibition as a therapeutic strategy to prevent morphine tolerance by restoring TRPC5 expression.

## 4 Experimental Section

### Patients and Clinical Data

Cerebrospinal Fluid (CSF) samples were collected from 16 participants at the First Affiliated Hospital of Nanjing Medical University. 16 individuals were included control group and morphine treatment group. Further information is included in Tables S1 and S2 (Supporting Information). All voluntary CSF donors provided informed consent under the Declaration of Helsinki guidelines and agreed to sample collection, storage, and analysis. The study was approved by the ethics committee of the First Affiliated Hospital of Nanjing Medical University (Nanjing, China; 2023‐SR‐041). Written informed consent was obtained from all participants.

### Animals

All procedures were performed in strict accordance with the regulations of the ethics committee of the International Association for the Study of Pain and the Guide for the Care and Use of Laboratory Animals (The Ministry of Science and Technology of China, 2006). All animal experiments were approved by the Nanjing Medical University Animal Care and Use Committee (NO. IACUC‐2201044 and NO. IACUC‐2112029) and were designed to minimize suffering and the number of animals used. Adult CD‐1, C57BL/6J mice (20–25 g) and Sprague‐Dawley rats (180–200 g) were provided by the Animal Core Facility of Nanjing Medical University, Nanjing, China. Only male animals were utilized in the experiments. *Trpc5* ^−/−^ mice were purchased from Cyagen, Suzhou, China. *R26^LSL‐DTR^* mice (C57BL/6) were purchased from Shanghai Model Organisms. All other experimental procedures, aside from CSF collection and neuronal cultures, were conducted using mice. C57BL/6J mice were used to investigate the role of *Trpc5* gene deletion in morphine tolerance, calcium regulation, and GABA release, while all other animal experiments were conducted using CD‐1 mice. Animals were housed five to six per cage under pathogen‐free conditions with soft bedding under controlled temperature (22 °C ± 2 °C) and photoperiods (12:12 h light‐dark cycle). The animals were habituated to these conditions for at least 2 days before starting experiments. Animals were randomly divided into groups. The sample size was designed on prior experience and to be limited to the minimal as scientifically justified.

### Genotyping

Tail biopsies were collected for genotyping using the One Step Mouse Genotyping Kit (Vazyme, PD101‐01) according to the manufacturer's instructions. A 349 bp fragment of *Trpc5* locus was amplified using the forward primer 5′‐ TGACCTCATGTAGGATAAGGGAATG ‐3′ and reverse primer 5′‐ CTCATGAATTTCCTTCCTTGGCCT‐3′. Additionally, a 622 bp fragment of *Trpc5* locus was amplified using the forward primer 5′‐ CAGATCTACCCAAACGGTCTAGTA‐3′ and reverse primer 5′‐CTCATGAATTTCCTTCCTTGGCCT‐3′. Amplified DNA fragments were visualized by gel electrophoresis. A single band at 349 bp indicates homozygosity for the mutant allele, while a single band at 622 bp indicates homozygosity for the wild‐type allele.

### Morphine Tolerance Model and Behavioral Testing

Intrathecal (i.t.) injection of morphine (10 µg/10 µL, H21022436, Shenyang First Pharmaceutical Co., Ltd.) or saline was administered from day 1 to day 7 to establish the mice model of morphine tolerance. The morphine doses were based on the previous studies.^[^ ^64^ ^]^ Behavioral testing was performed in a blinded manner 30 min after morphine administration during the light phase. The tail‐flick test (with the water bath temperature set to 52 °C ± 0.5 °C) was used to evaluate the analgesic effect of morphine. Mice were gently restrained, and 2 cm of the tail tip was submerged in the water bath. The latency (s) to reflexively withdraw the tail from the water was recorded as a positive nociceptive reflex response. A maximal cut‐off time of 10 s was applied for CD‐1 mice and 20 s for C57BL/6J mice to prevent tissue damage. Data were expressed as the percentage of maximal possible effect (%MPE), calculated using the following formula: 100% × [(Drug response time ‐ Basal response time) / (10/20 s ‐ Basal response time)] = %MPE. Rats were intrathecally injected with morphine (20 µg/10 µL, H21022436, Shenyang First Pharmaceutical Co., Ltd.) to establish the tolerance model.

### Morphine‐Induced Hyperalgesia Model and Behavioral Testing

Mice were subcutaneously administered with morphine (10 mg kg^−1^) twice daily for 7 consecutive days to establish a model of morphine‐induced hyperalgesia. Behavioral assessments were performed each morning before the first morphine injection. RLZ (2 µg/10 µL) or BTD (2 µg/10 µL) were administered intrathecally 30 min prior to the assay. The tail‐flick test (with the water bath temperature set to 48 °C ± 0.5 °C) was used to evaluate the analgesic effect of morphine. Mice were gently restrained, and 2 cm of the tail tip was submerged in the water bath. The latency (s) to reflexively withdraw the tail from the water was recorded as a positive nociceptive reflex response. A maximal cut‐off time of 20 s was applied for CD‐1 mice to prevent tissue damage.

### Open Field Tests

Mice were placed in the center of a transparent glass box (50 × 50 × 50 cm), and their movements were recorded for 5 min using an automated video tracking system (Clever Sys Inc.). Locomotor activity was scored based on the distance traveled and speed during the 5 min.

### Gait Analysis

Gait analysis was performed using the CatWalk XT gait analysis system (Noldus Information Technology Co., Ltd., Netherlands). Mice were trained to traverse a transparent glass walkway, during which their footprints were recorded by a high‐definition camera. To minimize external interference, the testing environment was kept dark to prevent ambient light from affecting the footprint capture. Quantitative gait parameters, including stride length (cm) and average speed (cm/s), were automatically calculated using CatWalk XT 10.7 software.

### Intrathecal Injection Procedure

To perform intrathecal (i.t.) injections, the mice or rats were placed in a prone position, and the midpoint between the tips of the iliac crest was located. A Hamilton syringe with a 30‐gauge needle was inserted into the subarachnoid space of the spinal cord between the L4 and L5 spinous processes. Proper intrathecal injection was systemically confirmed by observation of a tail flick in mice. Intrathecal injection did not affect baseline responses compared with latencies recorded before injection.

### Drug Treatment

To perform the tail‐flick test, mice were intrathecally injected with GABA_A_ receptor agonist (Etomidate, 5 µg/10 µL), GABA_B_ receptor agonist (Baclofen, 0.25 µg/10 µL), GABA_A_ receptor antagonist (Bicuculline, 5 µg/10 µL), TRPC5 selective activator (Riluzole, RLZ, 2 µg/10 µL; BTD, 2 µg/10 µL), TRPC5 selective inhibitor (AC1903, 2 µg/10 µL), EZH2 inhibitors (EPZ6438, 2 µg/10 µL; CPI1205, 2 µg/10 µL; UNC1999, 2 µg/10 µL) for 7 days with or without morphine (10 µg/10 µL, i.t.). For the GABA detection, rats were exposed to RLZ (4 µg/10 µL, i.t.) and EPZ6438 (4 µg/10 µL, i.t.), respectively with or without morphine (20 µg/10 µL, i.t.).

For immunoblotting or qPCR analysis, primary spinal neurons were pretreated with EPZ6438 (1 µM), CPI1205 (1 µM), UNC1999 (1 µM), DNA methyltransferase (DNMT) inhibitor‐Decitabine (5 µM), class I/II histone deacetylase (HDAC) inhibitor‐Trichostatin A (100 nM) and histone H3K9 methyltransferase inhibitor‐Chaetocin (100 nM) respectively for 72 h and then exposed to morphine (200 µM, 14 h) along with EPZ6438, CPI1205, UNC1999, Decitabine, Trichostatin A and Chaetocin. HEK293T cells were exposed to GABA (1 µM, 30 min), baclofen (1 µM, 30 min), and GABA_B_ receptor antagonist (CGP52432, 10 µM, 2 h). SH‐SY5Y cells were pretreated with EPZ6438 (1 µM) for 72 h and then exposed to morphine (200 µM, 14 h) along with EPZ6438.

Etomidate, Baclofen, Bicuculline, Riluzole, Decitabine, Trichostatin A, Chaetocin, GABA, and CGP52432 were purchased from MedChemExpress (HY‐B0100A, HY‐B0007, HY‐N0219, HY‐B0211A, HY‐A0004, HY‐15144, HY‐N2019, HY‐100067, and HY‐103531, Shanghai, China). BTD was purchased from Tocirs Bioscience (B86684‐04‐1). EPZ6438, CPI1205, and UNC1999 were purchased from Selleck (S7128, S8353, and S7165, Shanghai, China).

### RNA‐seq Analysis

Spinal cords from saline‐treated and morphine‐treated mice were collected by Trizol regent and subjected to RNA‐seq and data analysis by geneseeq (Nanjing, China). RNA‐seq data are available in Gene Expression Omnibus repository (GSE246582).

### RNA Isolation and qRT‐PCR

Total RNA was isolated from primary spinal neurons and spinal cords of mice using TRIzol regent (Invitrogen, 15 596 018, Carlsbad, California, USA). Isolated RNA was reverse‐transcribed into cDNA using HiScript II Q RT SuperMix for qPCR (Vazyme, R222‐01, Nanjing, China) following standard protocols. Quantitative real‐time PCR (qRT‐PCR) was performed with synthetic primers and ChamQ SYBR qPCR Master Mix (Vazyme, Q711‐02, Nanjing, China) with Quantstudio 5 Real‐Time PCR Detection System (Thermo Fisher Scientific). Expressed values relative to control were calculated using the ΔΔCt method. β‐actin was used as a housekeeping gene for normalization. The sequences of primers used for qPCR analysis were listed in Tables S3 and S4 (Supporting Information).

### Transient Transfection—*siRNAs* Knockdown

Small‐interfering RNA (siRNA) targeting human *EED* was purchased from Santa Cruz Biotechnology (sc‐63287, Danvers, Massachusetts, USA). siRNA targeting human *EZH2* (F: GAGGGAAAGUGUAUGAUAATT‐3′, R: 5′‐UUAUCAUACACUUUCCCUCTT‐3′), human *RbAp48* (5′‐ACUACUGCCGUAUGCCCUG‐3′ and 5′‐ CAGGGCATACGGCAGTAGT‐3′) and universal negative control siRNA were synthesized by GenePharma (Shanghai, China). SH‐SY5Y cells were plated at 70% confluence on the day of transfection. The transfection regent lipofectamine 2000 (Invitrogen, 11668‐019, Carlsbad, California, USA) was mixed with 100 pmol of siRNA and incubated for 5 h at 37 °C. Seventy‐two hours after transfection, the cells were treated with morphine for 14 h. The cell samples were collected for immunoblotting assay.

### Transient Transfection—Overexpression of K27M in H3.3 Mutation

The K27M mutant plasmid was constructed by a lysine 27‐to‐methionine mutation (K27M) in *H3F3A* from JustScience (Shanghai, China). The *H3F3A* WT sequence was ATGGCTCGTACAAAGCAGACTGCCCGCAAATCCACCGGTGGTAAAGCACCCAGGAAACAACTGGCTACAAAAGCCGCTCGCAAGAGTGCGCCCTCTACTGGAGGGGTGAAGAAACCTCATCGTTACAGGCCTGGTACTGTGGCCCTCCGTGAAATCAGACGCTATCAGAAGTCCACTGAACTTCTGATCCGCAAGCTCCCCTTTCAGCGTCTGGTGCGAGAAATTGCTCAGGACTTCAAAACAGATCTGCGCTTCCAGAGTGCAGCTATTGGTGCTTTGCAGGAGGCAAGTGAGGCCTATCTGGTTGGCCTTTTTGAAGATACCAATCTGTGTGCTATCCATGCCAAACGTGTAACAATTATGCCAAAAGATATCCAGCTAGCACGCCGCATACGCGGAGAACGTGCTGATTACAAGGACGACGATGACAAGTAA. The K27M mutant sequence was ATGGCTCGTACAAAGCAGACTGCCCGCAAATCCACCGGTGGTAAAGCACCCAGGAAACAACTGGCTACAAAAGCCGCTCGCATGAGTGCGCCCTCTACTGGAGGGGTGAAGAAACCTCATCGTTACAGGCCTGGTACTGTGGCCCTCCGTGAAATCAGACGCTATCAGAAGTCCACTGAACTTCTGATCCGCAAGCTCCCCTTTCAGCGTCTGGTGCGAGAAATTGCTCAGGACTTCAAAACAGATCTGCGCTTCCAGAGTGCAGCTATTGGTGCTTTGCAGGAGGCAAGTGAGGCCTATCTGGTTGGCCTTTTTGAAGATACCAATCTGTGTGCTATCCATGCCAAACGTGTAACAATTATGCCAAAAGATATCCAGCTAGCACGCCGCATACGCGGAGAACGTGCTGATTACAAGGACGACGATGACAAGTAA. SH‐SY5Y cells were transfected with H3F3A WT and K27M mutation plasmids for 72 h and then administrated with morphine for 14 h.

### Reporter Plasmid Construction and Activity Assays

The full length of human *TRPC5* promoter (Table S5, Supporting Information) were cloned into *pGL3*‐basic vector by JustScience (Shanghai, China) as the reporter. The construct was verified by DNA sequencing. The luciferase activity was assessed using a luciferase assay kit (Promega, E2920, Madison, Wisconsin, USA). Briefly, SH‐SY5Y cells were transfected with reporter plasmid and *p*SV‐β‐galactosidase control vector as well (Promega, E1081, Madison, Wisconsin, USA). 72 h after transfection, cells were treated with or without morphine for 14 h. The *TRPC5* reporter activity was normalized to that of *p*SV‐ β‐galactosidase.

### Viruses

pSLenti‐*Gad67*‐EGFP‐P2A‐TRPC5‐3xFLAG‐WPRE, pSLenti‐*Gad67*‐jGCaMP7f‐Puro‐WPRE, pSLenti‐*Gad67*‐EGFP‐3xFLAG‐miR30shRNA (*Ezh2*)‐WPRE, pAAV‐*Gad67*‐mCherry‐3xFLAG‐WPRE, pAAV‐*hSyn*‐jGCaMP7f‐WPRE, pAAV‐hSyn‐iGABASnFR.F102G‐WPRE, pAAV‐*Gad67*‐EGFP‐P2A‐Cre‐WPRE viruses and control vectors were purchased from OBiO Technology (Shanghai, China).

### Immunoblot Analysis

Samples (cells or spinal cord tissue) were collected and washed with ice‐cold PBS before being lysed in lysis buffer (Beyotime Biotechnology, P0013B, Shanghai, China). Sample lystes were separated by SDS‐PAGE and electrophoretically transferred onto polyvinylidene fluoride membranes (Millipore, 000 0229836, Temecula, California, USA). The membranes were blocked with 10% milk or with 5% BSA and 5% milk in TBST (Tris‐HCl, NaCl, Tween 20) for 2 h at room temperature and probed with primary antibody at 4 °C overnight. The primary antibodies were specific for β‐actin (ABclonal, AC026, 1:10 000, Wuhan, China), Na^+^/K^+^ ATPase (Abmart, MU135312, 1:3000, Shanghai, China), GAPDH (Santa Cruz Biotechnology, sc‐32233, 1:2000, Danvers, Massachusetts, USA), TRPC5 (ABclonal, A10089, 1:500, Wuhan, China), Histone H3 (ABclonal, A2348, 1:1000, Wuhan, China), TriMethyl‐Histone H3‐K27 (H3K27me3) (ABclonal, A2363, 1:1000, Wuhan, China), EZH2 (ABclonal, A16846, 1:1000, Wuhan, China), GAD67 (Synaptic Systems, 198 211, 1:1000, Göttingen, Germany), RBAP48 (ABclonal, A3645, 1:1000, Wuhan, China) and EED (ABclonal, A12773, 1:1000, Wuhan, China). Finally, horseradish peroxidase (HRP)‐coupled secondary antibodies (Sigma, AP307P and AP308P, 1:5000, Darmstadt, Germany) were utilized to detect the corresponding primary antibody. The bands were developed by enhanced chemiluminescence reagents (New Cell & Molecular Biotech Co., Ltd, China). Data was acquired with a molecular imager chemidocxrs system (ChemiDoc XRS+, Bio–Rad) and analyzed with Image J (1.51 version, Rawak Software).

### Isolation and Culture of Cortical Neurons from Mice

Cortical neurons were obtained from embryonic day 13–15 (E13‐15, where E0 is the day of vaginal plug) of gestation, immediately after the mice were sacrificed with an over‐dose of barbital. The mice embryos were stored in ice‐cold HBSS (Keygen Biotech, KGM14175‐2, Nanjing, China) until dissection. After removal of meninges, the embryos were dissected out and then the cortex was transferred into a new culture tissue with ice‐cold HBSS supplemented with 1 mm HEPES (Sangon Biotech, E607018‐0100, Shanghai, China), 80 U mL^−1^ penicillin and 0.08 mg mL^−1^ streptomycin (Keygen Biotech, KGY0023, Nanjing, China). Following washing, the cortex was cut into 0.5 mm sections and incubated in HBSS containing 0.25% trypsin (Gibco, 15090‐046, Grand Island, Nebraska, USA) and 150 unit DNase (Sigma, D5025‐15KU, St. Louis, Missouri, USA) at 37 °C for 12 min. Then, cortex was washed in warm HBSS supplemented with 1 mm HEPES, 80 U mL^−1^ penicillin and 0.08 mg mL^−1^ streptomycin three times.

Cortex neurons were seeded into confocal dishes precoated with poly D‐lysine (PDL, Gibco, A38904‐01, Frederick, Maryland, USA). The cells were plated in Neurobasal plating medium (Gibco, 21103‐049, Paisley, UK) supplemented with 2% B‐27 plus supplement (Gibco, A35828‐01, Grand Island, Nebraska, USA), 10% horse serum heat inactivated (HiHS, Gibco, 26050‐070, Penrose, Auckland, New Zealand), 2 mM L‐glutamine (Sigma, G7513, UK), 1 mM HEPES, 80 U mL^−1^ penicillin and 0.08 mg mL^−1^ streptomycin. After 18 h, the Neurobasal plating medium was replaced with Neurobasal growth medium comprising 2% B‐27 plus supplement, 2 mM L‐glutamine, 80 U mL^−1^ penicillin and 0.08 mg mL^−1^ streptomycin. Cells were incubated at 37 °C in a 95% air/5% CO_2_ humidified atmosphere. All procedures were described previously.^[^ ^65^ ^]^

### Isolation and Culture of Neurons from Embryonic Spinal Cord

Pregnant animals were sacrificed at E15 (E0 = first day following the mating day) with an over‐dose of barbital. The rat embryos were stored in ice‐cold L15‐medium (Keygen Biotech, KGL1802‐500, Nanjing, China) until dissection. After removal of meninges, the embryos were dissected out and then spinal cords were transferred into a new culture tissue with ice‐cold L15‐medium supplemented with 1 mm HEPES, 80 U mL^−1^ penicillin and 0.08 mg mL^−1^ streptomycin. Following washing, spinal cords were cut into 0.5 mm sections and incubated in HBSS containing 0.25% trypsin and 150 U mL^−1^ DNase at 37 °C for 12 min. Then, the spinal cords were washed in warm HBSS supplemented with 1 mM HEPES and 80 U mL^−1^ penicillin, and 0.08 mg mL^−1^ streptomycin three times.

Neurons were seeded into 6, 12, or 24‐well plates precoated with PDL. The cells were plated in Neurobasal plating medium supplemented with 2% B‐27 plus supplement, 10% HiHS, 2 mM L‐glutamine, 1 mM HEPES, 80 U mL^−1^ penicillin and 0.08 mg mL^−1^ streptomycin. After 18 h, the Neurobasal plating medium was replaced with Neurobasal growth medium comprising 2% B‐27 plus supplement, 2 mM L‐glutamine, 80 U mL^−1^ penicillin and 0.08 mg mL^−1^ streptomycin. Cells were incubated at 37 °C in a 95% air/5% CO_2_ humidified atmosphere. All procedures were described previously.^[^ ^66^ ^]^

### Cell Line Culture

Undifferentiated human neuroblastoma cell line SH‐SY5Y (CRL‐2266) and HEK‐293T cell line (CRL‐3216) were purchased from American type culture collection (ATCC).

SH‐SY5Y cells were cultured in a 5% CO_2_ atmosphere at 37 °C in Dulbecco's modified Eagle Media: a ratio 1:1 ratio of MEM (Gibco, 11 095 080, Grand Island, Nebraska, USA), and F12 (Gibco, 11765‐054, Paisley, UK) supplemented with 10% (v/v) FBS (Biochannel, BC‐SE‐FBS01, Nanjing, China), 80 U mL^−1^ penicillin and 0.08 mg mL^−1^ streptomycin. Cells were plated in 6, 24, or 96‐well plates overnight. For differentiation, SH‐SY5Y cells were treated with 60 µM retinoic acid (MCE, HY‐14649, Shanghai, China) for 48 h. Cell extracts and supernatants were collected and analyzed. HEK‐293T cells were cultured in a 5% CO_2_ atmosphere at 37 °C in Dulbecco's modified Eagle Media: DMEM (Keygen Biotech, KGL1211‐500, Nanjing, China) supplemented with 10% (v/v) FBS (Biochannel, BC‐SE‐FBS01, Nanjing, China), 80 U mL^−1^ penicillin and 0.08 mg mL^−1^ streptomycin. For further transfection, cells were plated in 6‐well plates overnight. Cell extracts were collected and analyzed.

### Intracellular Calcium Levels Measurement

SH‐SY5Y cells were plated in 96‐well plates for intracellular calcium levels ([Ca^2+^] _i_) measurement. Cells were exposed to retinoic acid and then treated with morphine. The growth medium was removed and then cells and 8 wells without cells as blank control were incubated with dye loading buffer (80 mL well^−1^) containing 5 µm Fluo‐4 (Life Technologies, F14201), 0.02% Pluronic F‐127 (Beyotime, ST5001‐1 g, Shanghai, China) and 0.25 mM sulfinpyrazone (MCE, HY‐B1271, Shanghai, China) in calcium‐free kreb's buffer for 60 min. Cells wells without cells were washed by calcium‐free kreb's buffer comprising sulfinpyrazone three times and incubated with sulfinpyrazone in calcium‐free kreb's buffer for 30 min. After 30 min of incubation, 96‐well plates were transferred to a fluorescence laser plate reader (FLIPR Tetra; Molecular Devices, Sunnyvale, CA, USA) incubation chamber and the emitted fluorescence were recorded at 521 nm after excitation at 488 nm (F _base_ and F_0_). For dynamic measurement, cells were treated with or without RLZ in kreb's buffer containing 10 µM Nifedipine and 1 µM Cav 2.2 blocker. Fluorescence (F _test_) were recorded for 15 min at an interval of 30 s. The Fluo‐4 fluorescence intensity from each group was expressed as (F _test_ ‐ F_0_)/ (F _base_ ‐ F_0_).

### Histological Analysis

Mice were anesthetized and perfused transcardially with normal saline followed by 4% paraformaldehyde (PFA) in 0.1 m phosphate buffer, pH 7.4. Then, lumbar 5 DRG and lumbar 4‐5 spinal cord were dissected out and postfixed in 4% paraformaldehyde for 24 h.

For immunofluorescence, DRG or spinal cord tissues were transversely cut into 15‐µm‐thick sections and then the sections were blocked with 10% normal donkey serum (Jackson ImmunoResearch, 017‐000‐121, West Grove, Pennsylvania, USA) and 0.3% triton X 100 in PBS for 2 h at room temperature. Anti‐neurofilament H (ABclonal, A8442, 1:50, Wuhan, China), anti‐CGRP (Cell Signaling Technology, 14959s, 1:300, Danvers, Massachusetts, USA), anti‐IB4 (Sigma, L2140, 1:1000, St. Louis, Missouri, USA), anti‐TRPC5 (Invitrogen, PA5‐77310,1:100, Rockford, Illinois, USA), anti‐GAD67 (Synaptic Systems, 198 211, 1:300), anti‐GABA (Sigma, A0310, 1:300, St. Louis, Missouri, USA), anti‐VGLUT2 (Invitrogen, MA5‐27613, 1:50, Rockford, Illinois, USA), anti‐Iba1 (Abcam, ab5076, 1:300, Cambridge, Massachusetts, USA), anti‐NeuN (Millipore, MAB377X, 1:2000, Temecula, California, USA), anti‐GFAP (Santa Cruz, sc‐6170, 1:300, Dallas, Texas, USA) were used as primary antibodies to incubate with sections overnight at 4 °C. After three rinses of PBS, sections were incubated with Alexa Fluor 594 AffiniPure Donkey Anti‐Mouse IgG (H+L) (Jackson ImmunoResearch, 715‐585‐150, 1:300, West Grove, Pennsylvania, USA), donkey anti‐rabbit Alexa Fluor 488 IgG (H+L) (Invitrogen, A21206, 1:1000, Eugene, Oregon, USA) and Cy3 AffiniPure Donkey Anti‐Goat IgG (H+L) (Jackson ImmunoResearch, 705‐165‐147, 1:300, West Grove, Pennsylvania, USA) for 2 h at room temperature, and the nuclei were stained with DAPI (Invitrogen, P36931, Eugene, Oregon, USA). The images were captured by Zeiss 880 confocal microscopy. Data analysis was performed blindly.

For immunohistochemistry, the paraffin‐embedded mice spinal cords were cut into 5‐µm‐thick sections, and the sections were deparaffinized, rehydrated, antibody retrieved, blocked, and then stained with anti‐TRPC5 (Invitrogen, PA5‐77310, 1:100, Rockford, Illinois, USA) overnight at 4 °C. After three rinses of 1× PBS, sections were incubated with HRP‐conjugated goat anti‐rabbit IgG (Abcam, ab207995). Staining was visualized by TissueFAXS Plus. Data analysis was performed blindly.

### Immunocytochemistry

Primary spinal neurons were cultured and plated on PDL coated coverslips. Cells were fixed with 4% PFA for 15 min and blocked with 10% normal donkey serum and 0.3% triton X 100 in PBS for 2 h at room temperature. Anti‐TRPC5 (Proteintech, 25890‐1‐AP, 1:100), anti‐GABA (Sigma‐Aldrich, A0310, 1:300) and anti‐GAD67 (Synaptic Systems, 198 211, 1:1000, Göttingen, Germany) were used as primary antibodies to incubate with cells at 4 °C overnight. After washing 3 times with PBS, coverslips were treated with donkey anti‐rabbit Alexa Fluor 488 IgG (H+L) (Invitrogen, A‐21206, 1:1000, Eugene, Oregon, USA) and Alexa Fluor 594 AffiniPure Donkey Anti‐Mouse IgG (H+L) (Jackson ImmunoResearch, 715‐585‐150, 1:300, West Grove, Pennsylvania, USA) for 2 h at room temperature, and the nuclei were stained with DAPI. The images were captured by Zeiss 880 confocal microscopy. Data analysis was performed blindly.

### Collection of the Cerebrospinal Fluid (CSF) of Rats

All procedures were in strict accordance with the published method.^[^ ^67^ ^]^ Before CSF collection, rats were deeply anesthetized with 1% pentobarbital sodiumand then the fur on the neck region was removed. The anesthetized rats were placed in a stereotaxic frame and were secured with ear bars. The position of rat head was maintained downward at ≈45°. A depressible surface with the appearance of a rhomb between the occipital protuberance and the spine of the atlas became visible. Needle connected to a draw syringe was inserted horizontally and centrally into the cisterna magna for CSF collection without making any incision at this region. A gentle aspiration would make the CSF flow through the needle. The colorless CSF sample was slowly drawn into the syringe and color of the CSF was closely observed to avoid any possible blood contamination. Upon the appearance of blood contamination, the PE tubing was pinched off above the contamination site and the tubing was cut at this point. The non‐contaminated samples were drawn into the syringe.

### ELISA—Human CSF

Samples were collected from 16 patients at the First Affiliated Hospital of Nanjing Medical University for GABA detection.

### ELISA—Rats CSF

Rats were intrathecally injected with RLZ and EPZ6438, respectively paired with morphine for 7 continuous days, and CSF were collected for GABA detection.

### ELISA—Cell Supernatants

Primary spinal neurons and SH‐SY5Y cells were stimulated as indicated. Supernatants were collected for GABA detection.

GABA detection was in accordance with manufacturer's instructions with Human GABA ELISA Kit (Abcam, ab287792) and Rat GABA ELISA Kit (Abcam, ab287814).

### Isolation of Membrane Protein

Membrane Protein Extraction kit (Thermo Scientific, 89 842) was used to isolate membrane protein according to the manufacturer's instructions. Briefly, primary spinal neurons were collected and washed with washing buffer. After centrifugation, permeabilization buffer was added to the cell pellet to resuspend cells and incubated at 4 °C for 10 min. Solubilization buffer was added to the pellet and incubated at 4 °C for 30 min. After centrifugation, supernatants containing solubilized membrane and membrane‐associated proteins were transferred to a fresh tube.

### Quantitative Chromatin Immunoprecipitation (qChIP)

SH‐SY5Y cells were cultured and plated in 10 cm culture dish. Separate groups of cells were used for qChIP experiment. 2 × 10^7^ SH‐SY5Y cells were collected for each immunoprecipitation. Cells were cross‐linked with 1% formaldehyde for 10 min at room temperature and quenched with glycine solution for 5 min at room temperature. And then cells were washed two times with 20 mL ice‐cold PBS and scraped into ice‐cold PBS containing protease inhibitor cocktail (PIC). Next, centrifuging cells at 1000 x g for 5 min at 4 °C and removing PBS to collect cells. One milliliter of sonication cell lysis buffer containing PIC was added to per chromatin preparation to suspend cells. Cell suspensions were incubated in sonication cell lysis buffer containing PIC on ice for 10 min. Cells were centrifuged at 5000 x g for 5 min at 4 °C and resuspended in 1 mL sonication cell lysis buffer containing PIC. Cell suspension was incubated on ice for 5 min and then centrifuged at 5000 x g for 5 min at 4 °C. Next, samples were resuspended in 1 mL of ice‐cold ChIP sonication nuclear lysis buffer containing PIC and incubated on ice for 10 min. Samples were sonicated to ∼500 bp. And then the chromatin samples were centrifuged at 21 000 x g for 10 min at 4 °C and supernatants were transferred to a new tube for ChIP assay. Antibody to EZH2 (cell signaling technology, 5246, 1:100), antibody to H3K27me3 (Cell Signaling Technology, 9733, 1:50) and antibody to RNA polymerase II (Millipore, 05‐623, 1:100, Temecula, California, USA) were added to each chromatin sample over at 4 °C with rotation. Normal mouse IgG (Santa Cruz Biotechnology, sc‐2025, 1:50, Danvers, Massachusetts, USA) and normal rabbit IgG (Santa Cruz Biotechnology, sc‐2027, 1:50, Danvers, Massachusetts, USA) were performed as controls. Thirty microliters of protein G magnetic beads was added to each IP reaction for 2 h at 4 °C with rotation. Then elute chromatin from beads for 30 min at 65 °C and transfer the supernatants to new tubes for qPCR. Primers for qChIP were shown in Table S6 (Supporting Information). All reactions were analyzed using 2% x 2^(C[T] 2%Input Sample – C[T] IP Sample)^ with 2% input as a normalization control.

### In Situ Hybridization

Mice were anesthetized and perfused transcardially with normal saline followed by 4% PFA in PBS. After the perfusion, spinal cords were removed and post‐fixed in 4% PFA in PBS overnight at 4 °C. The tissues were then cryopreserved in 10% sucrose in PBS for 8 h, followed by 20% sucrose in PBS for 1 day, and 30% sucrose in PBS for 1 day. The embedded blocks were sectioned at a thickness of 15 µm. In situ hybridization was performed using the RNAscope system (Advanced Cell Diagnostics) following the manufacturer's protocol. Pretreatment consisted of dehydration, followed by incubation with hydrogen peroxide at room temperature and protease III or plus at 40 °C. The Multiplex Fluorescent Kit v2 protocol was followed using commercial probes for *Trpc5*, *Slc32a1*, and *Slc17a6*. In the combined application of the RNAscope Multiplex Fluorescent v2 Assay and immunofluorescence, antibodies against IBA‐1 (CST, #17198T; 1:50), NeuN (CST, #24 307; 1:50), and GFAP (Santa Cruz, sc‐6170; 1:50) were utilized. In situ *hybridization* images were captured by Zeiss 880 confocal microscopy.

### Chloride Imaging in Mice Primary Neurons

Rat primary spinal neurons were subjected to 200 µM morphine for 14 h in growth medium and then the medium was replaced with kreb's buffer containing 50 µM RLZ for 2 h. The supernatants were collected to stimulate primary cortical neurons. Primary cortical neurons were incubated with the 5 mM Cl^−^ indicator 1‐(Ethoxycarbonylmethyl)‐6‐methoxyquinolinium bromide (MQAE) (Biofount, 162558‐52‐3) in Cl^−^‐free kreb's buffer for 2 h. Then, extracellular MQAE was washed three times with Cl^−^‐free kreb's buffer. MQAE images of each group were captured in the absence of Cl^−^ as the baseline (F_0_). Then the Cl^−^‐free kreb's buffer was switched to different supernatants from primary spinal neurons. During the period of 5 min, MQAE images were acquired every 15 s (F). The mean MQAE fluorescence from each group was expressed as ΔF’ (F‐ F_0_). The images were captured by Zeiss Axio Observer 2. The mean fluorescence intensity was measured using Image J. Data analysis was performed blindly.

### Calcium Imaging in Rat Primary Spinal Neurons

Primary spinal neurons were transfected with calcium indicator pSLenti‐*Gad67*‐jGCaMP7f‐Puro‐WPRE for 96 h and stimulated as indicated for live cell imaging. Images were captured in the context of calcium‐free kreb's buffer containing L‐type calcium channel inhibitor (Selleck, S1808, Nifedipine, 1 µM) and N‐type calcium channel inhibitor (MCE, HY119373, Cav 2.2 blocker 1, 10 µM) as the baseline (F_0_). The calcium‐free kreb's buffer was switched to calcium‐containing kreb's buffer with Nifedipine and Cav 2.2 blocker 1. The process of Ca^2+^ influx was recorded for 5 min. Images were obtained every 15 s (F). The average fluorescence intensity was expressed as ΔF (F‐F_0_). After a period of 5 min, the solution was replaced with calcium‐containing kreb's buffer with RLZ (50 µM), Nifedipine and Cav 2.2 blocker 1. The process of Ca^2+^ influx was recorded for 5 min. Images were obtained every 15 s (F_1_). The images were captured by Zeiss Axio Observer 2. The average fluorescence intensity was expressed as ΔF’ (F_1_‐F). The fluorescence intensity was quantified by Image J. Data analysis was performed blindly.

### Ex Vivo Experiments for Multiphoton Imaging

Ca^2+^ imaging of GABAergic interneurons in the superficial dorsal horn was performed in mice. Mice were intrathecally injected with pAAV‐*Gad67*‐mCherry‐3xFLAG‐WPRE (OBiO Technology, Shanghai, China) to label GABAergic interneurons and pAAV‐hSyn‐jGCaMP7f‐WPRE (OBiO Technology, Shanghai, China) to indicate calcium. After 21 d, to assess the effect of morphine on calcium influx in GABAergic interneurons, mice were subjected to morphine for 7 days and then were deeply anesthetized with 1% pentobarbital sodium (100 mg kg^−1^, i.p.) before spinal cord preparation. Mice were transcardially perfused with 95%O_2_/5%CO_2_ saturated, chilled, sucrose‐based artificial cerebrospinal fluid (ACSF). The sucrose‐based ACSF contained (in mM): 234 sucrose, 2.5 KCl, 0.5 CaCl_2_, 10 MgSO_4_, 1.25 NaH_2_PO_4_, 26 NaHCO_3_, and 11 Glucose. Then, the spinal cord was removed from the bone and washed with standard normal ACSF solution containing (in mM): 117 NaCl, 3.6 KCl, 2.5 CaCl_2_, 1.2 MgCl_2_, 1.2 NaH_2_PO_4_, 25 NaHCO_3_, and 11 glucose. To assess the effect of TRPC5 on calcium influx in GABAergic interneurons, the preparation of spinal cord was the same as described above. Imaging was performed on Olympus FVMPE‐RS using water immersion lens. Data analysis was performed blindly.

GABA imaging was performed in the superficial dorsal horn of mice. Mice were intrathecally injected with pAAV‐*Gad67*‐mCherry‐3xFLAG‐WPRE (OBiO Technology, Shanghai, China) to label GABAergic interneurons and pAAV‐hSyn‐iGABASnFR.F102G‐WPRE (OBiO Technology, Shanghai, China) to indicate released GABA. After 21 d, to assess the effect of morphine or TRPC5 on GABA release in superficial dorsal horn, the drug treatment and the preparation of spinal cord were the same as described above. Imaging was performed on Olympus FVMPE‐RS using water immersion lens. Data analysis was performed blindly.

### Statistics Analysis

All data were represented as the mean ± SD. The analysis was performed with GraphPad Prism (version 9) and Origin 2021. Two groups were compared by a two‐tailed Student's *t* test. Comparisons among groups were performed by one‐way ANOVA followed by Tukey's post hoc test. Comparisons among groups with multiple time points were performed by two‐way ANOVA followed by Tukey's post hoc test. Statistical significance was defined at *P* values < 0.05.

## Conflict of Interest

The authors declare no conflict of interest.

## Author Contributions

C.J., W.L., and X.W. designed and supervised the research. L.W., M.Z., H.G., and F.H. performed the laboratory experiments. L.W., M.Z., and H.G. analyzed the data. Y.X. and Y.P. collected samples in clinic. C.X. and F.H. contributed to experimental design. L.W. and C.J. wrote the manuscript.

## Supporting information



Supporting Information

## Data Availability

The data that support the findings of this study are available from the corresponding author upon reasonable request.
