# Supplementary material for: Morphine Tolerance Gated through EZH2‐Mediated Suppression of Trpc5 in Spinal GABAergic Interneurons in Male Mice
Source: Adv Sci (Weinh). 2025 Oct 20;13(1):e07908. doi: 10.1002/advs.202507908 (PMC12766986; doi:10.1002/advs.202507908)
Supplement: Supplementary file 1 — Supporting Information [file ADVS-13-e07908-s001.docx]

**Supporting Information for**

**Morphine tolerance gated through EZH2-mediated suppression of *Trpc5* in spinal GABAergic interneurons in male mice**

Li Wan ^1, 2^, Mengyao Zhang^1^, Haiyue Guo^1^, Yan Xu^3^, Chenjie Xu^4^, Fan Hu^1^, Yinbing Pan^5^, Xian Wang^6, 🖂^, Wentao Liu^1, 🖂^ and Chun-Yi Jiang^1†, 🖂^

**Author affiliations:**

1 Jiangsu Key Laboratory of Neurodegeneration, Department of Pharmacology, Nanjing Medical University, Nanjing, Jiangsu, China.

2 State Key Laboratory of Pharmaceutical Biotechnology, School of Life Sciences, Nanjing University, Nanjing, Jiangsu, China.

3 Department of Pain, the first people ‘s hospital of Changzhou, Soochow University, Changzhou, Jiangsu, China.

4 Department of Anesthesiology and Pain, Nanjing First Hospital, Nanjing Medical University, Nanjing, Jiangsu, China.

5 Department of Anesthesiology, The First Affiliated Hospital of Nanjing Medical University, Nanjing, Jiangsu, China.

6 Department of Anesthesiology, Women’s Hospital of Nanjing Medical University, Nanjing Women and Children’s Healthcare Hospital, Nanjing, Jiangsu, China

^🖂^Correspondence to:

Chun-Yi Jiang

**E-mail:** [jcy@njmu.edu.cn](mailto:jcy@njmu.edu.cn).

Wentao Liu

**E-mail:** [painresearch@njmu.edu.cn](mailto:painresearch@njmu.edu.cn).

Xian Wang

**E-mail:** wangxian2002@126.com

^†^ Lead contact


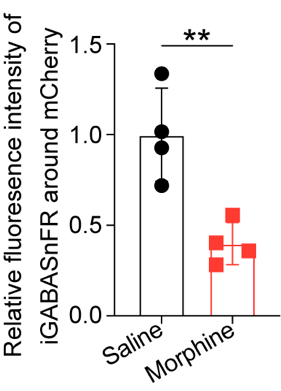


**Figure. S1. Quantitative analysis of GABA release following chronic intrathecal morphine administration.** Data showed decreased GABA release around GABAergic interneurons (labeled with mCherry) after chronic morphine exposure (10 μg/10 μL, for 7 days) compared to the saline-treated group (Unpaired Student’s *t* test, *t* (6) = 4.264, *P* = 0.0053, *n* = 4). Data are expressed as mean ± SD, ***P* < 0.01.


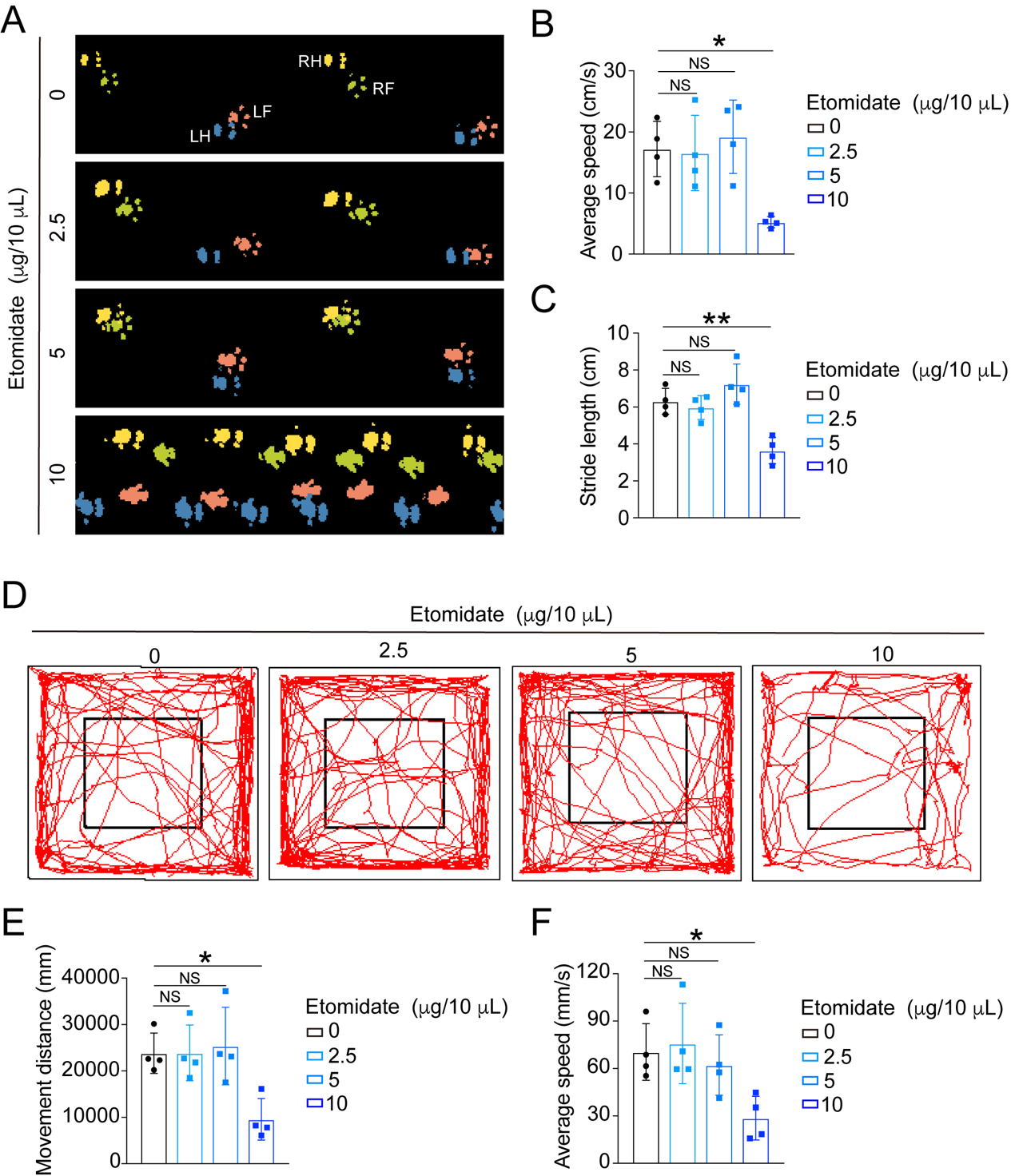


**Figure. S2. Intrathecal injection of etomidate impaired motor activity at the dose of 10 μg/10 μL, but not 2.5 μg/10 μL and 5 μg/10 μL in mice.** **(A)** The representative images of footprint in gait analysis (*n* = 4). **(B and C)** Intrathecal injection of etomidate (10 μg/ 10μL) decreased average speed (cm/s) and stride length (cm) of mice compared to the control group in the gait analysis (B: one-way ANOVA, *F* (3, 12) = 6.682, *P* = 0.0067, *n* = 4; C: one-way ANOVA, *F* (3, 12) = 14.37, *P* = 0.0003, *n* = 4). **(D)** The representative images of travel path in open filed tests (*n* = 4); **(E and F)** Intrathecal injection of etomidate (10 μg/ 10μL) decreased movement distance (mm) and average speed (mm/s) of mice in open filed tests (*n* = 4; E: one-way ANOVA, *F* (3, 12) = 6.079, *P* = 0.0093 *n* = 4; F: one-way ANOVA, *F* (3, 12) = 4.719, *P* = 0.0213, *n* = 4). Data are expressed as mean ± SD, **P* < 0.05.


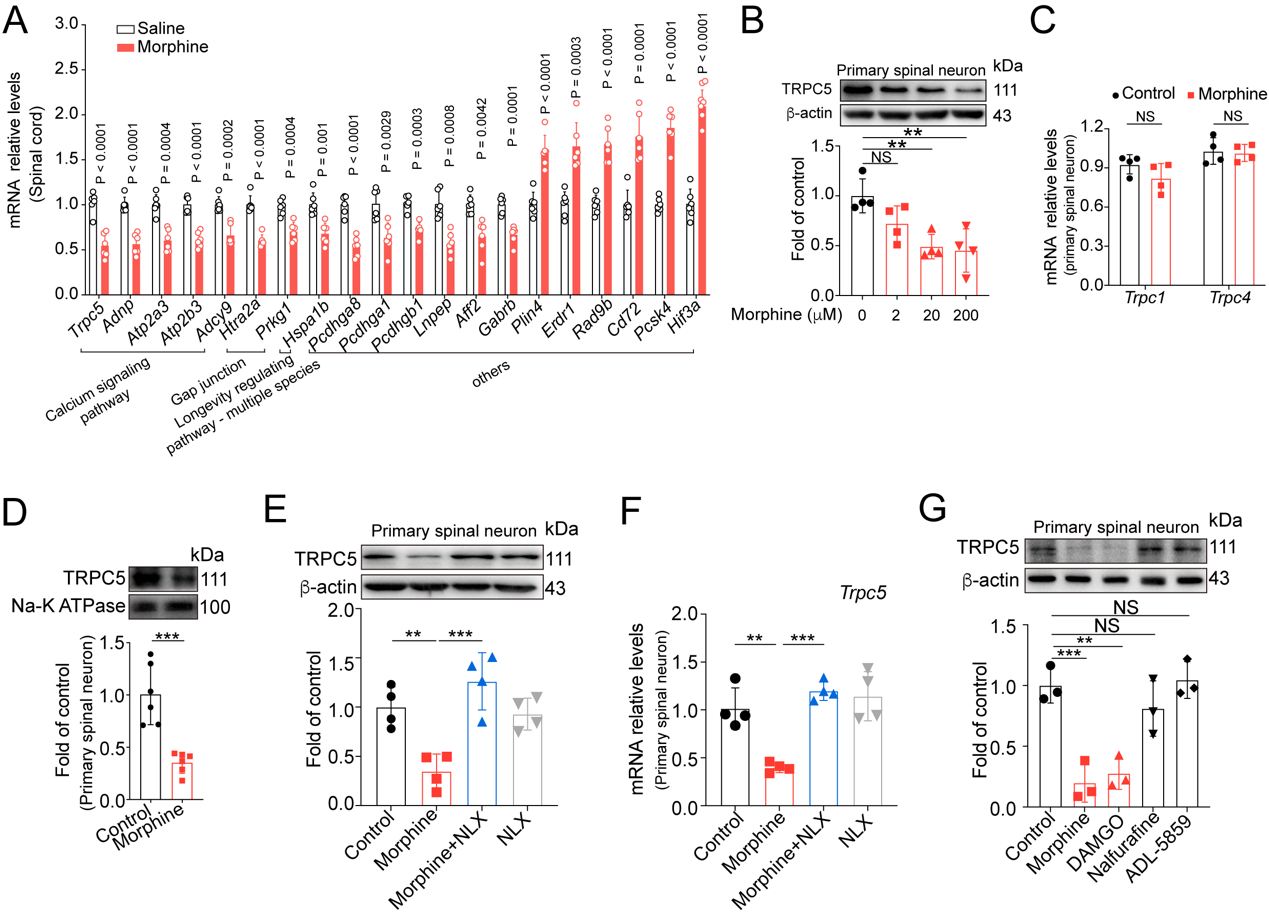


**Figure. S3. Morphine induces downregulation of TRPC5 in a mu opioid receptor-dependent manner.** **(A)** Mice were intrathecally injected with morphine (10 μg/10 μL) for sustained 7 days to establish morphine tolerance model and L4-L5 sections of the spinal cords were collected for qPCR. Data showed the mRNA levels of *Trpc5*, *Adnp*, *Atp2a3*, *Adcy9*, *Htra2a*, *Prkg1*, *Hspa1b*, *Pcdhga8*, *Pcdhgb1*, *Pcdhga1*, *Lnpep*, *Aff2*, *Gabrb*, *Plin4*, *Erdr1*, *Rad9b*, *Cd72*, *Pcsk4* and *Hif3a* (*n* = 6). **(B)** Primary spinal neurons were exposed to 2, 20, 200 μM morphine respectively for 14 h. Immunoblotting data displayed that both 20 μM and 200 μM morphine resulted in the downregulation of TRPC5 expression, but 2 μM morphine had no effect on TRPC5 expression (one-way ANOVA, *F* (3,12) = 8.250, *P* = 0.003, *n* = 4). **(C)** Morphine had no effect on *Trpc1 and Trpc4* mRNA levels (*Trpc1*: Unpaired Student’s *t* test, *t* (6) = 1.582, *P* = 0.1647, *n* = 4; *Trpc4*: Unpaired Student’s *t* test, *t* (6) = 0.2467, *P* = 0.8133, *n* = 4). **(D)** Immunoblot analysis revealed downregulation of TRPC5 protein levels on cell membranes isolated from primary spinal neurons after morphine exposure (200 μM, 14 h) (Unpaired Student’s *t* test, *t* (10) = 7.374, *P* = 0.0001, *n* = 6). **(E and F)** Neurons were pretreated with naloxone (NLX, 10 μM) for 12 h and then exposed to morphine (200 μM, 14 h) along with NLX. The samples were collected for immunoblotting and qPCR assays respectively. (E) NLX abolished the suppressive effect of morphine on TRPC5 (one-way ANOVA, *F* (3,12) = 12.91, *P* = 0.0005, *n* = 4). (F) NLX abrogated the effect of morphine on *Trpc5* transcriptional suppression (one-way ANOVA, *F* (3,12) = 17.35, *P* = 0.0001, *n* = 4). **(G)** Neurons were subjected to morphine (200 μM, 14 h), DAMGO (10 μM, 14 h), nalfurafine (10 μM, 14 h) and ADL-5859 (200 μM, 14 h) respectively. Data showed that morphine and DAMGO suppressed TRPC5 expression, however, nalfurafine and ADL-5859 had no effect on TRPC5 expression (one-way ANOVA, *F* (4,10) = 17.52, *P* = 0.0002, *n* = 3). Data are expressed as mean ± SD, ***P* < 0.01 and ****P* < 0.001.


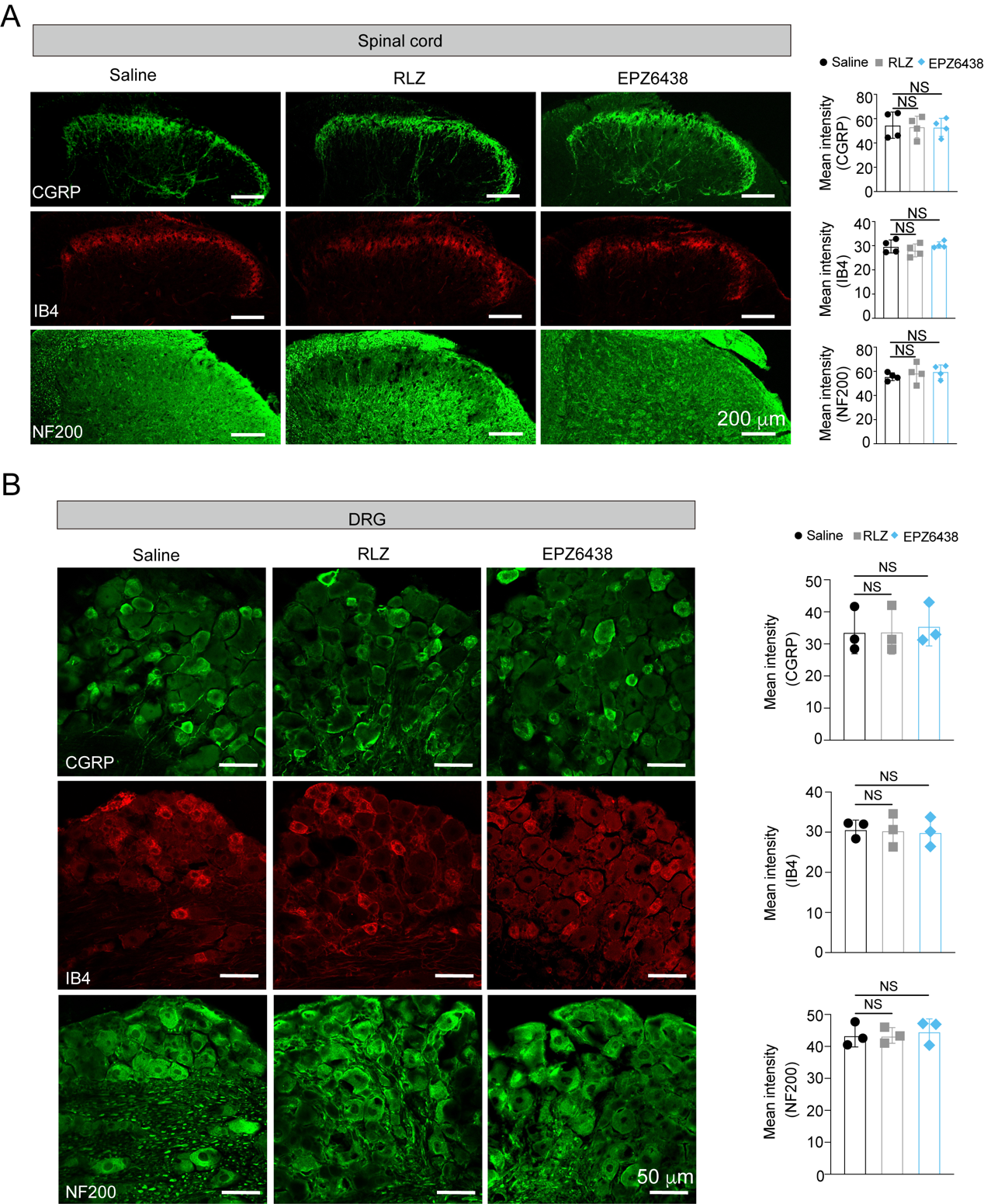


**Figure. S4. Mice exhibit normal nerve innervations, neurochemical expression in the spinal cord and DRG after treatment with RLZ and EPZ6438.** **(A)** Immunostaining showed CGRP^+^, IB4^+^ and NF200^+^ central terminals in the spinal cord from saline-treated, RLZ-treated and EPZ6438-treated mice respectively (CGRP: one-way ANOVA, *F* (2, 9) = 0.04242, *P* = 0.9587, *n* = 4; IB4: one-way ANOVA, *F* (2, 9) = 0.9776, *P* = 0.4218, *n* = 4; NF200: one-way ANOVA, *F* (2, 9) = 0.4798, *P* = 0.6339, *n* = 4). **(B)** The images of immunofluorescence showed the expression of CGRP, IB4 and NF200 in DRG from saline-treated, RLZ-treated and EPZ6438-treated mice respectively (CGRP: one-way ANOVA, *F* (2, 6) = 0.07322, *P* = 0.9302, *n* = 3; IB4: one-way ANOVA, *F* (2, 6) = 0.03542, *P* = 0.9654, *n* = 3; NF200: one- way ANOVA, *F* (2, 6) = 0.4798, *P* = 0.6339, *n* = 3). Data are expressed as mean ± SD.

**
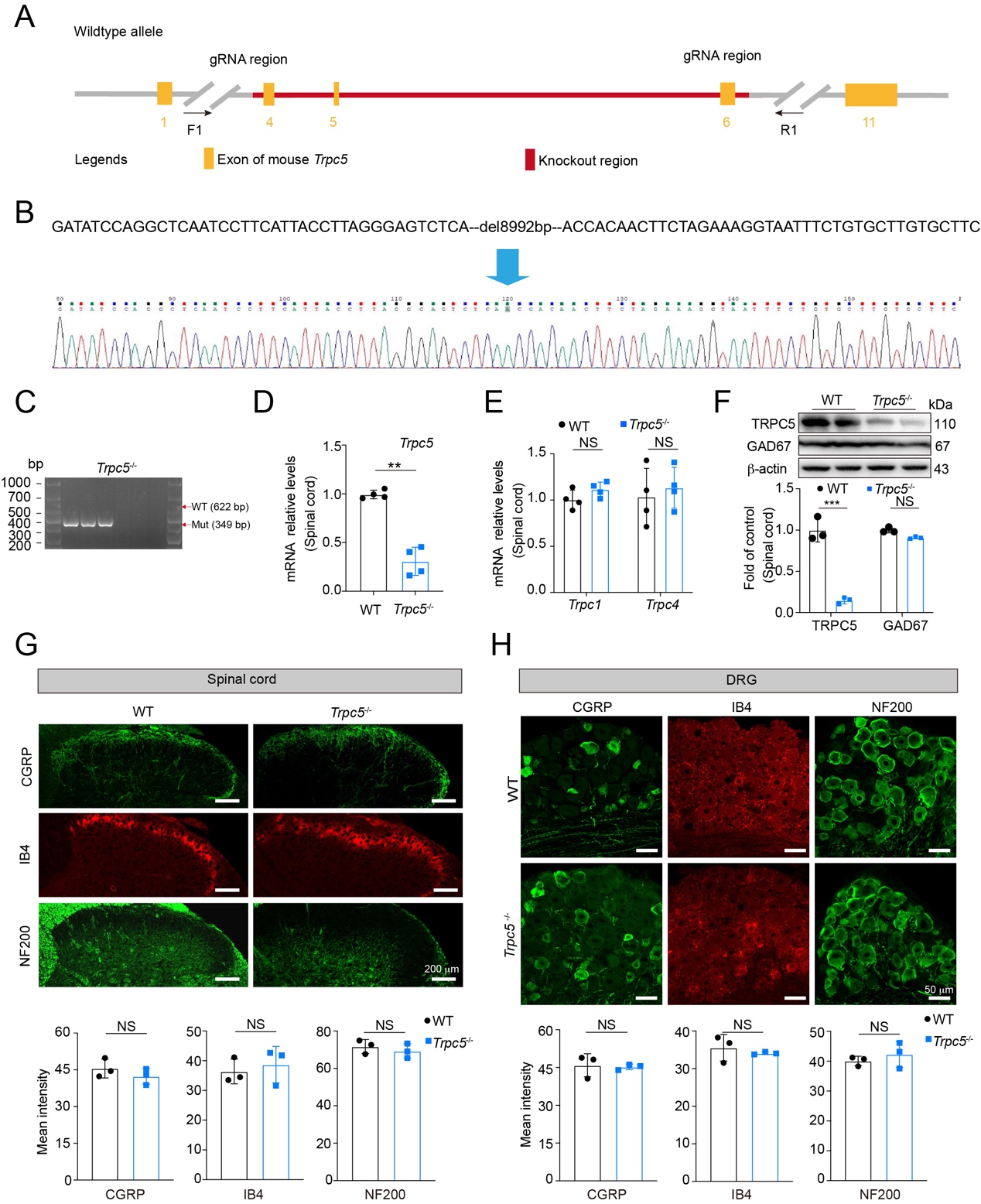
**

**Figure. S5. *Trpc5*^-/-^ mice was generated mediated by CRISR/Cas9 and exhibit normal nerve innervations，neurochemical expression in the spinal cord and DRG. (A)** A schematic showed Cas9-mediated genome editing strategy. Two sgRNAs are designed to target the mice *Trpc5* locus*.* gRNA-A1 (matching reverse strand of gene): TCACCAGGTCCATTCCGGATGGG, gRNA-A2 (matching forward strand of gene): TAGCCTACTAGACCAACTTAAGG. This paired sgRNAs are designed to delete the DNA sequence in *Trpc5* gene from exon 4 to exon 6 (red labeled). **(B)** DNA- sequencing showed the deletion of 8992 bases in chimera mice. **(C)** PCR-based genotyping of *Trpc5*^-/-^ mice. **(D)** qPCR data displayed that the levels of *Trpc5* mRNA was downregulated in *Trpc5*^-/-^ mice compared with WT mice (Unpaired Student’s *t* test, *t* (6) = 9.102, *P* < 0.0001, *n* = 4). **(E)** qPCR data showed that *Trpc5* deficiency did not affect *Trpc1 and Trpc4* mRNA levels (*Trpc1*: Unpaired Student’s *t* test, *t* (6) = 1.659, *P* = 0.1428, *n* = 4; *Trpc4*: Unpaired Student’s *t* test, *t* (6) = 0.5257, *P* = 0.6180, *n* = 3). **(F)** Immunoblotting data showed that TRPC5 expression was decreased and GAD67 expression did not change in *Trpc5*^-/-^ mice compared with WT mice (TRPC5: Unpaired Student’s t test, *t* (4) = 16.45, *P* < 0.0001, *n* = 3; GAD67: Unpaired Student’s *t* test, *t* (4) = 4.409, *P* = 0.0116, *n* = 3). **(G)** The immunostaining of CGRP, IB4 and NF200 in the spinal cord of *Trpc5*^-/-^ mice and WT mice (CGRP: Unpaired Student’s *t* test, *t* (4) = 1.095, *P* = 0.3352, *n* = 3; IB4: Unpaired Student’s *t* test, *t* (4) = 0.5381, *P* = 0.6190, *n* = 3; NF200: Unpaired Student’s *t* test, *t* (4) = 0.7779, *P* = 0.4801, *n* = 3). **(H)** The immunostaining of CGRP, IB4 and NF200 in the DRG of *Trpc5*^-/-^ mice and WT mice (CGRP: Unpaired Student’s *t* test, *t* (4) = 0.2343, *P* = 0.8263, *n* = 3; IB4: Unpaired Student’s *t* test, *t* (4) = 0.7089, *P* = 0.5175, *n* = 3; NF200: Unpaired Student’s *t* test, *t* (4) = 0.8231, *P* = 0.4567, *n* = 3). Data are expressed as mean ± SD.


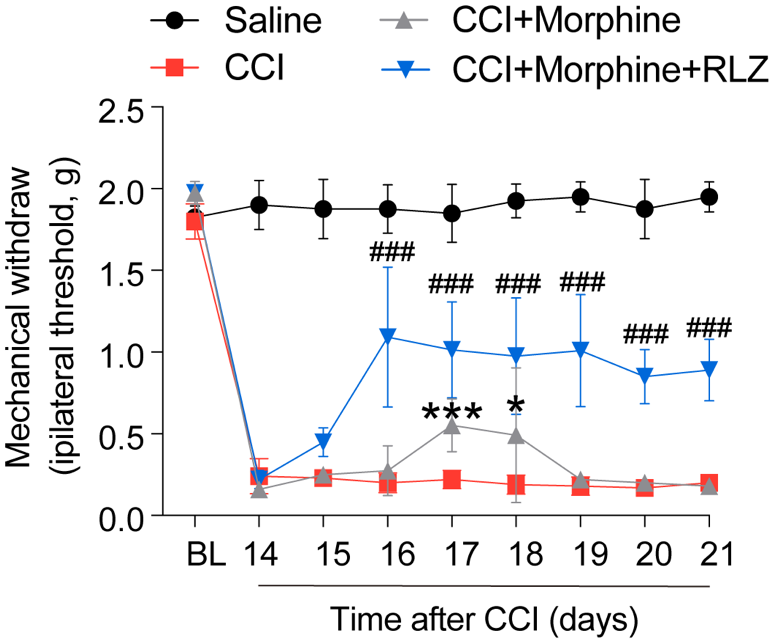


**Figure. S6. TRPC5 activation enhances the therapeutic effect of morphine on CCI-induced neuropathic pain.** Fourteen days after CCI surgery, the mice were intrathecally injected with morphine (10 μg /10 μL) once daily for 7 consecutive days, with or without RLZ (2 μg /10 μL), behavioral testing was performed 30 min after injection. Data indicated that, following the final injection, the mechanical threshold in the RLZ plus morphine treatment group increased from 0.2 g to 1.0 g, significantly higher than that observed in the morphine-only group (two-way ANOVA, drug effect: *F* (3, 253) = 1188, *P* < 0.0001; time effect: *F* (8, 253) = 155.9, *P* < 0.0001; drug × time effect: *F* (24, 253) = 26.87, *P* < 0.0001, *n* = 8 mice). Data are expressed as mean ± SD, CCI versus CCI + morphine, **P* < 0.05 and ****P* < 0.001; CCI + morphine versus CCI + morphine + RLZ, ^###^*P* < 0.001.

**
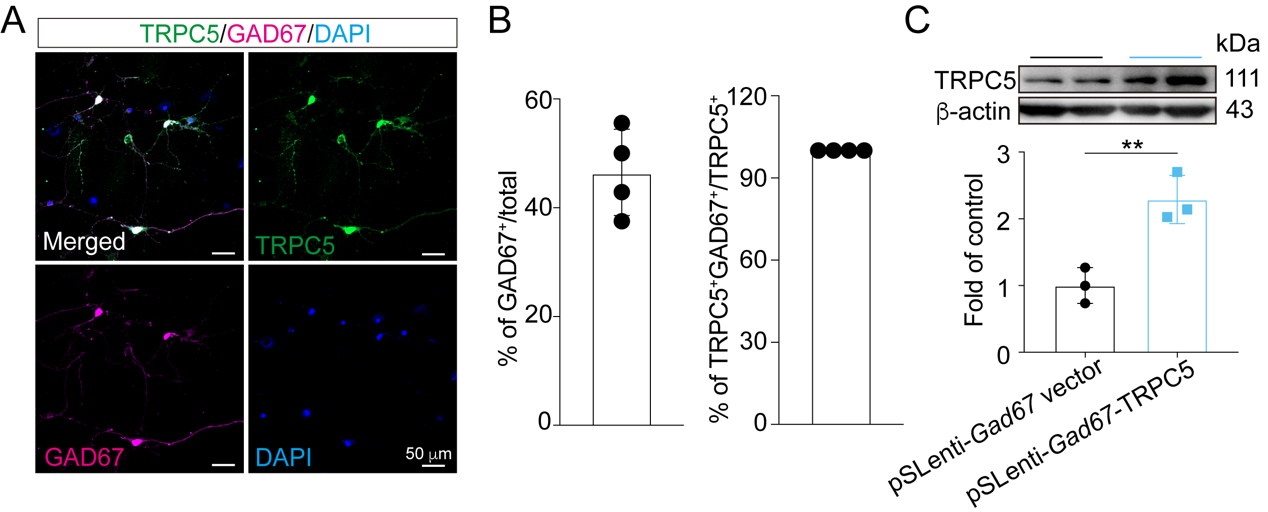
**

**Figure. S7. TRPC5 is expressed in GAD67^+^ neurons.** **(A)** Primary spinal neurons were isolated form rats for immunofluorescence staining. Representative immunofluorescent images showed that TRPC5 were co-stained with GAD67. **(B)** The proportion of GAD67^+^ neurons and the ratio of TRPC5^+^ GAD67^+^ within TRPC5^+^ neurons. **(C)** Primary spinal neurons were transfected with pSLenti-TRPC5 or its control vector for 96 h. The efficiency of transfection was examined by immunoblotting (Unpaired Student’s *t* test, *t* (4) = 4.971, *P* = 0.0076, *n* = 3). Data are expressed as mean ± SD, ***P* < 0.01.


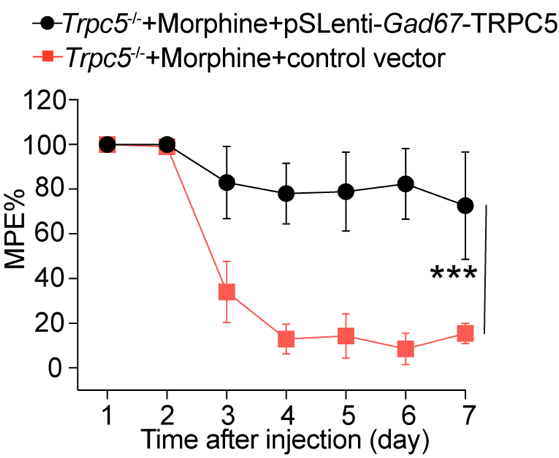


**Figure. S8. TRPC5 overexpression in spinal GABAergic interneurons improves morphine analgesic effects in *Trpc5*-deficient mice.** *Trpc5***^-/-^** mice received intrathecal injections of pSLenti-*Gad67*-TRPC5 or control vectors 1 week before intrathecal morphine injection (10 μg/10 μL, for 7 day). Behavioral testing was performed 30 min after morphine injection each day. Data are expressed as mean ± SD, ****P* < 0.001.


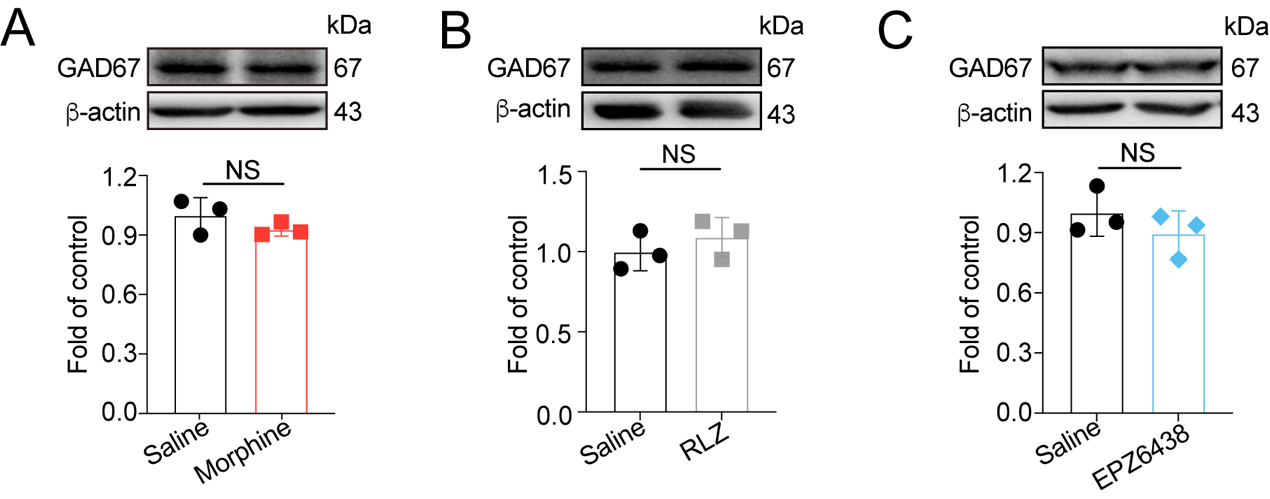


**Figure. S9. GAD67 expression do not change in primary spinal neurons after exposure to morphine, RLZ and EPZ6438 respectively.** **(A)** Primary spinal neurons were treated with morphine (200 μM, 14 h). The samples were collected for immunoblotting and data showed morphine did not affect GAD67 expression (Unpaired Student’s *t* test, *t* (4) = 1.308, *P* = 0.2608, *n* = 3). **(B)** Primary spinal neurons were treated with RLZ (50 μM, 2 h). The immunoblotting data showed RLZ had no effect on GAD67 expression (Unpaired Student’s *t* test, *t* (4) = 0.9048, *P* = 0.4168, *n* = 3). **(C)** Primary spinal neurons were subjected to EPZ6438 (1 μM, 72 h). The immunoblotting data showed that EPZ6438 had no effect on GAD67 expression (Unpaired Student’s *t* test, *t* (4) = 1.116, *P* = 0.3268, *n* = 3). Data are expressed as mean ± SD.


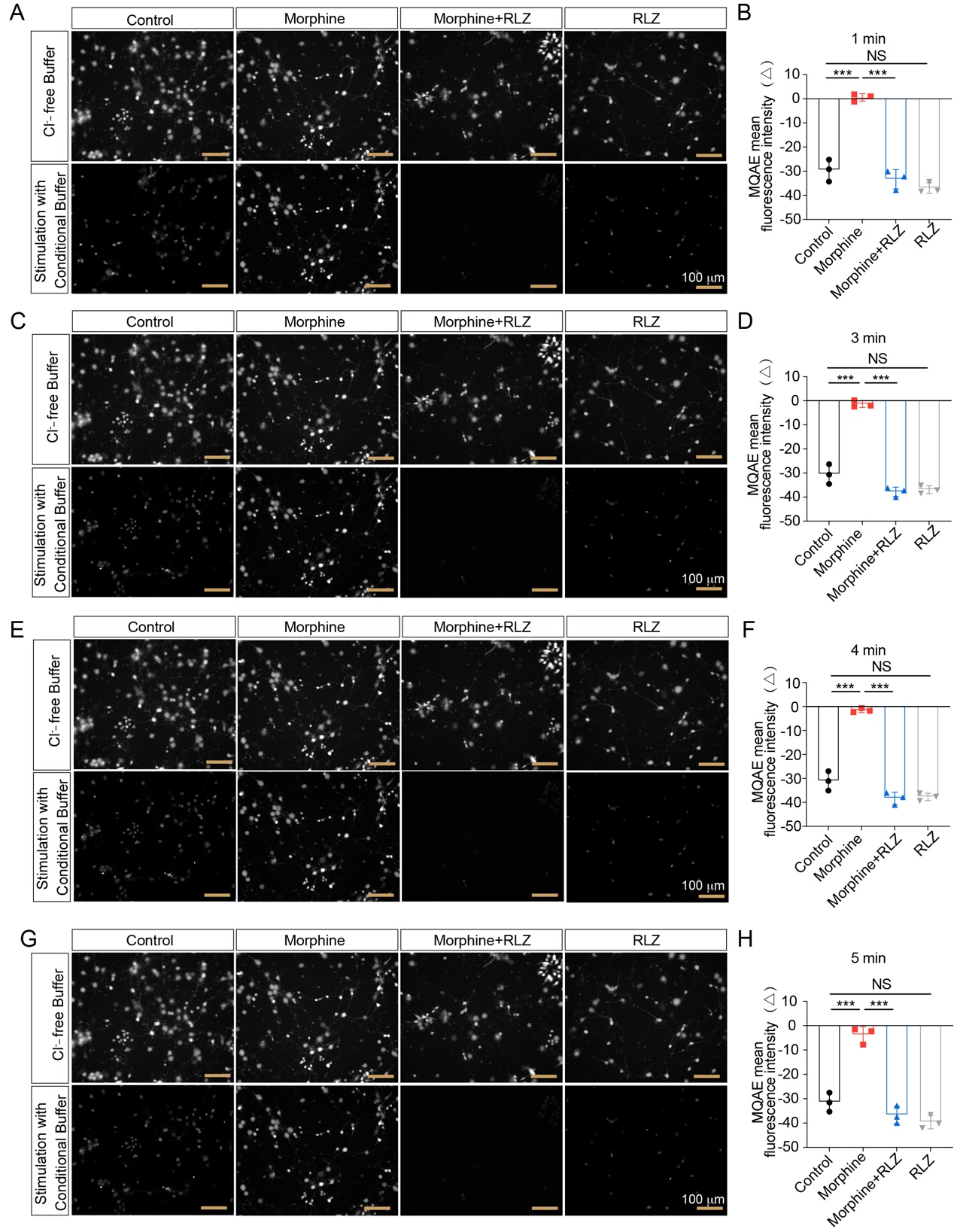


**Figure. S10. Chloride imaging analysis of primary cortex neurons after stimulation with conditional supernatants at 1 min, 3 min, 4 min and 5 min.** Primary spinal neurons were administrated with morphine (200 μM, 14 h) and then treated with RLZ in kreb’s buffer (50 μM, 2 h). The supernatants were collected to stimulate cortex neurons loaded with the Cl^-^-sensitive dye MQAE (5 μM, 2 h). **(A, C, E and G)** Representative images showed the chloride influx after stimulation with supernatants. (**B**) Fluorescence quantitative analysis displayed a lower rising of chloride influx and the inhibition of Cl^-^ influx was abolished by RLZ 1 min after stimulation with supernatants (one-way ANOVA, *F* (3, 8) = 80.19, *P* < 0.0001, *n* = 3). **(D)** Fluorescence quantitative analysis displayed a lower rising of chloride influx and the inhibition of Cl^-^ influx was abolished by RLZ 3 min after stimulation with supernatants (one-way ANOVA, *F* (3, 8) = 138.9, *P* < 0.0001, *n* = 3). **(F)** Fluorescence quantitative analysis displayed a lower rising of chloride influx and the inhibition of Cl^-^ influx was abolished by RLZ 4 min after stimulation with supernatants (one-way ANOVA, *F* (3, 8) =138.0, *P* < 0.0001, *n* = 3). **(H)** Fluorescence quantitative analysis displayed a lower rising of chloride influx and the inhibition of Cl^-^ influx was abolished by RLZ 5 min after stimulation with supernatants (one-way ANOVA, *F* (3, 8) = 67.95, *P* < 0.0001, *n* = 3). Data are expressed as mean ± SD, ****P* < 0.001.


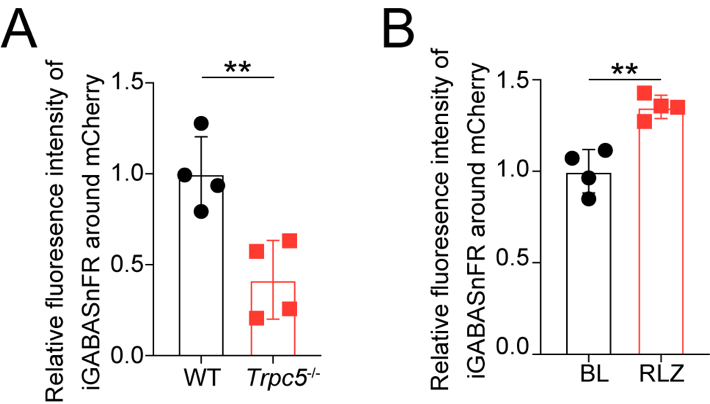


**Figure. S11. Quantitative analysis of GABA release. (A)** Data showed decreased GABA release around GABAergic interneurons (labeled with mCherry) in *Trpc5*^-/-^ mice compared to WT mice (Unpaired Student’s *t* test, *t* (6) = 3.922, *P* = 0.0078, *n* = 4). **(B)** Data showed increased GABA release around GABAergic interneurons (labeled with mCherry) within 1 minute of RLZ treatment compared to baseline conditions (Unpaired Student’s *t* test, *t* (6) = 5.228, *P* = 0.002, *n* = 4). Data are expressed as mean ± SD, ***P* < 0.01.


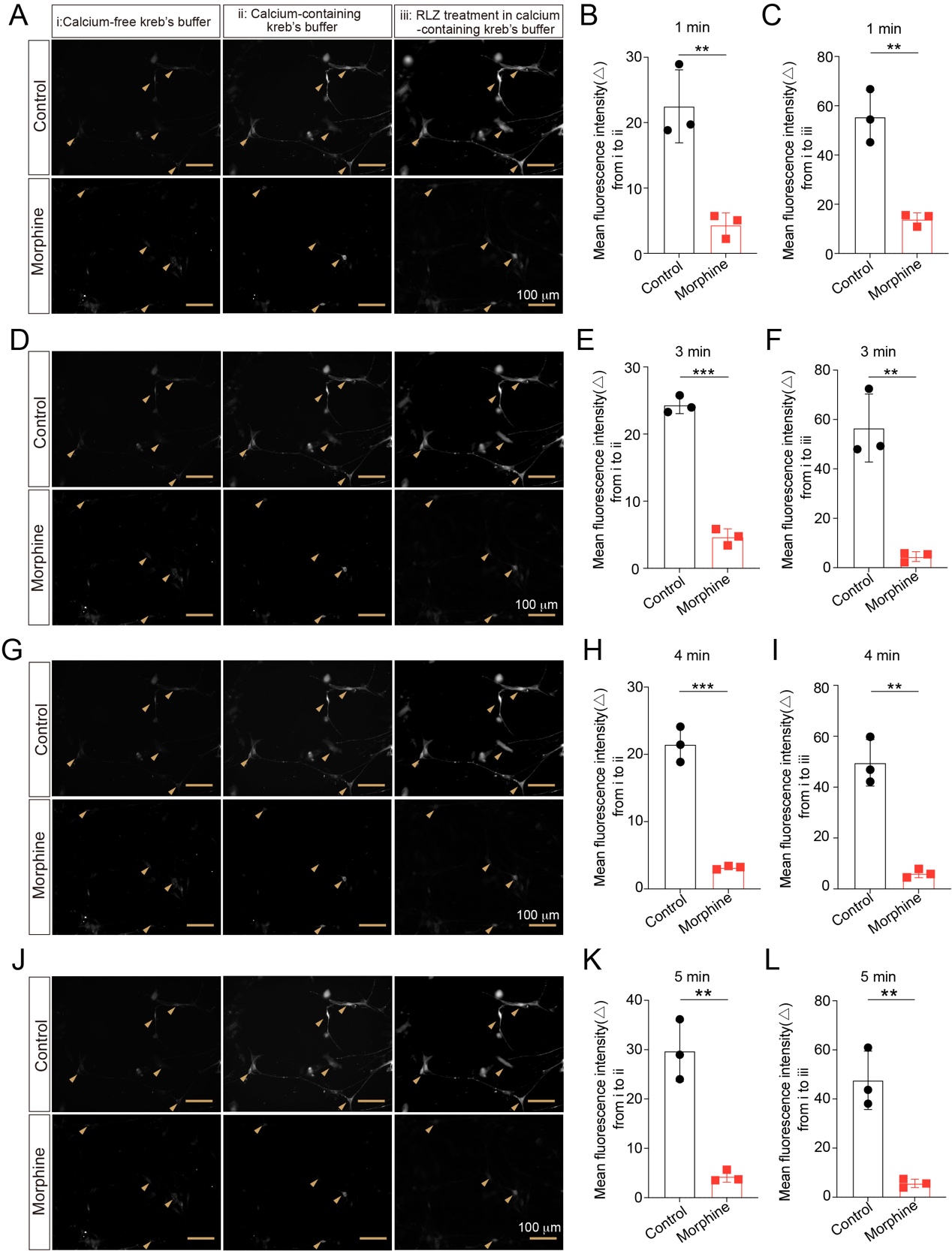


**Figure. S12. Calcium imaging analysis of the effect of RLZ on Ca^2+^ influx in primary spinal neurons after treatment with morphine at 1 min, 3 min, 4 min and 5 min.** Primary spinal neurons were transfected with calcium indicator pSLenti-*Gad67*- jGCaMP7f-Puro-WPRE and then exposed to morphine (200 μM, 14 h). Calcium imaging of spinal primary neurons displayed Ca^2+^ influx in morphine-treated neurons and control neurons. **(A, D, G and J)** Representative images showed that (i) neurons were incubated with calcium-free kreb’s buffer; (ii) and then the neurons were incubated with calcium-containing kreb’s buffer for 5 min. Representative images showed the Ca^2+^ influx at 1, 3, 4, 5 min; (iii) Then RLZ was added to the neurons in calcium-containing kreb’s buffer. Representative images displayed the Ca^2+^ influx at 1, 3, 4, 5 min. **(B and C)** Fluorescence quantitative analysis displayed that decreased Ca^2+^ influx emerged in morphine-treated neurons compared with control group 1 min after incubation with calcium-containing kreb’s buffer (B: Unpaired Student’s *t* test, *t* (4) = 5.343, *P* = 0.0059, *n* = 3; C: Unpaired Student’s *t* test, *t* (4) = 6.477, *P* = 0.0029, *n* = 3). **(E and F)** Fluorescence quantitative analysis displayed that decreased Ca^2+^ influx emerged in morphine-treated neurons compared with control group 3 min after incubation with calcium-containing kreb’s buffer (E: Unpaired Student’s *t* test, *t* (4) = 19.22, *P* < 0.0001, *n* = 3; F: Unpaired Student’s *t* test, *t* (4) = 6.468, *P* = 0.0029, *n* = 3). **(H and I)** Fluorescence quantitative analysis displayed that decreased Ca^2+^ influx emerged in morphine-treated neurons compared with control group 4 min after incubation with calcium-containing kreb’s buffer (H: Unpaired Student’s *t* test, *t_4_* = 12.04, *P* = 0.0003, *n* = 3; I: Unpaired Student’s *t* test, *t* (4) = 8.099, *P* = 0.0013, *n* = 3). **(K and L)** Fluorescence quantitative analysis displayed that decreased Ca^2+^ influx emerged in morphine-treated neurons compared with control group 5 min after incubation with calcium-containing kreb’s buffer (K: Unpaired Student’s *t* test, *t* (4) = 7.062, *P* = 0.0021, *n* = 3; L: Unpaired Student’s t test, *t* (4) = 6.046, *P* = 0.0038, *n* = 3). Data are expressed as mean ± SD, **P* < 0.05, ***P* < 0.01 and ****P* < 0.001.


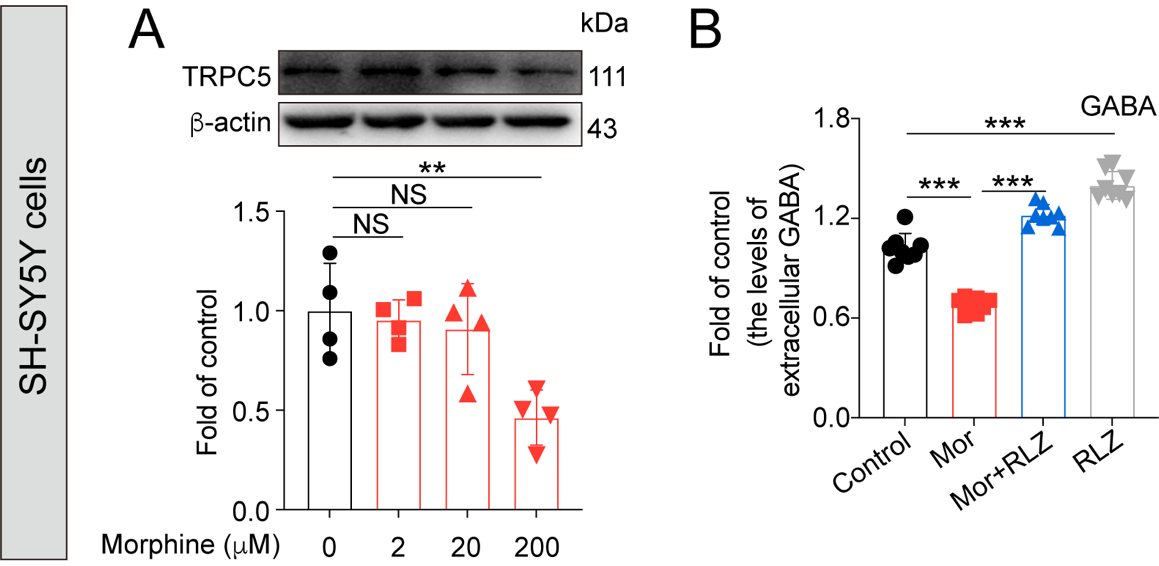


**Figure. S13. The effect of RLZ on morphine induced decreased GABA release in SH-SY5Y cells.** **(A)** SH-SY5Y cells were subjected to morphine (2 μM, 20 μM and 200 μM) for 14 h, respectively. The cells extracts were collected for immunoblotting. Data showed that TRPC5 expression was decreased after 200 μM morphine exposure and 2 μM, 20 μM morphine did not change the levels of TRPC5 (one-way ANOVA, *F* (3, 12) = 7.128, *P* = 0.0053, *n* = 4). **(B)** SH-SY5Y cells were exposed to morphine (200 μM, 14 h) and then treated with RLZ (50 μM, 2 h). The supernatants were collected for GABA detection by ELISA. Data showed that morphine inhibited GABA release and RLZ abolished the suppressive effect of morphine on GABA release (one-way ANOVA, *F* (3, 28) = 149.0, *P* < 0.0001, *n* = 8). Data are expressed as mean ± SD, ***P* < 0.01 and ****P* < 0.001.


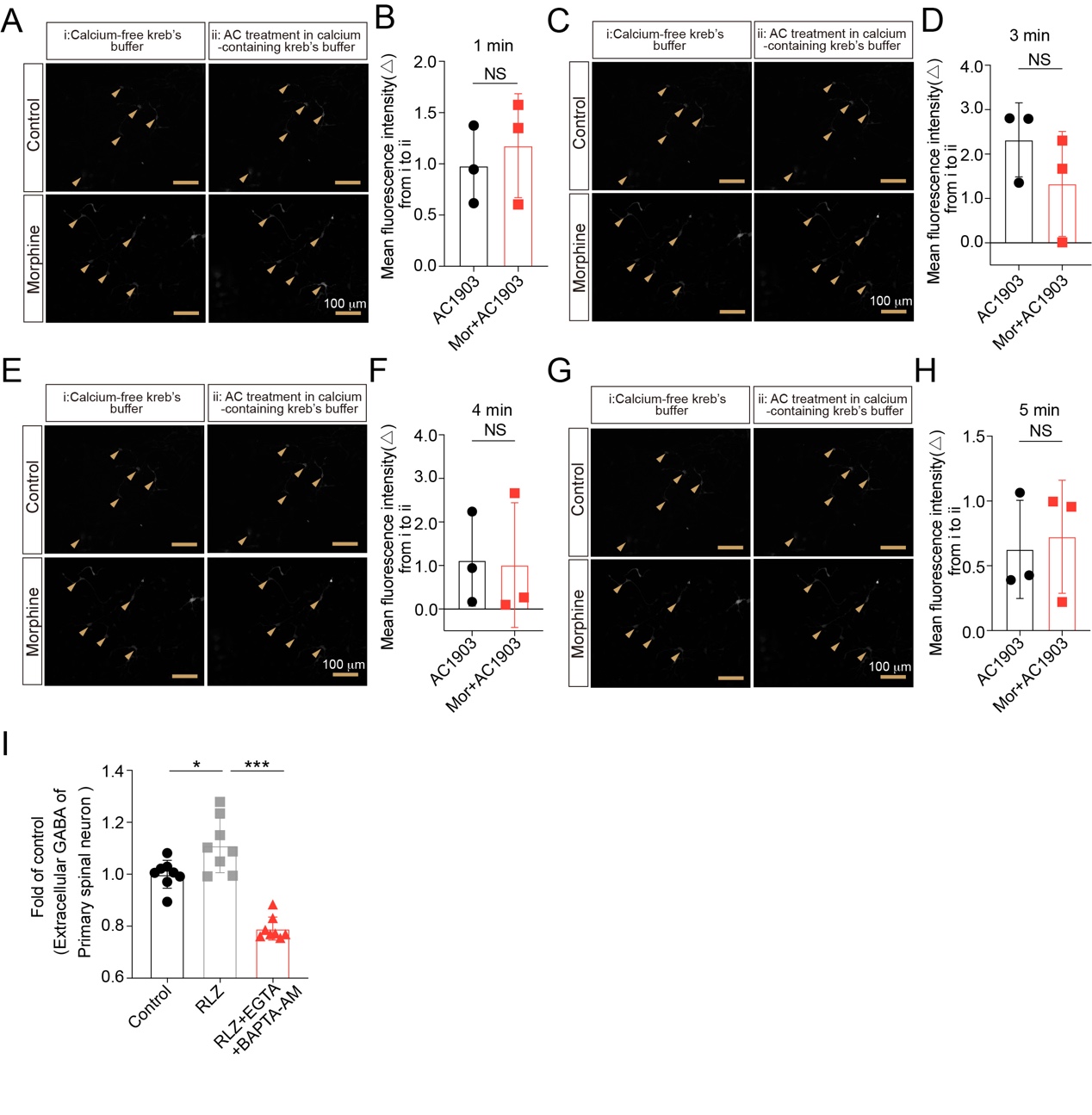


**Figure. S14.** **Calcium imaging analysis of the effect of AC1903 on Ca^2+^ influx in primary spinal neurons after treatment with morphine at 1 min, 3 min, 4 min and 5 min.** **(A, C, E and G)** Primary spinal neurons were transfected with calcium indicator pSLenti-*Gad67*-jGCaMP7f-Puro-WPRE and then subjected to morphine (200 μM, 14 h). Calcium imaging showed Ca^2+^ influx in morphine-treated neurons and control neurons. Representative images showed that (i) neurons were incubated with calcium- free kreb’s buffer; (ii) and then the neurons were treated with AC1903 (TRPC5 antagonist, 50 μM) in calcium-containing kreb’s buffer for 5 min. Representative images showed the Ca^2+^ influx at 1, 3, 4, 5 min. **(B)** Fluorescence quantitative analysis displayed that decreased Ca^2+^ influx emerged in morphine-treated neurons compared with control group 1 min after treatment with AC1903 in calcium-containing kreb’s buffer (1 min: Unpaired Student’s *t* test, *t* (4) = 0.54, *P* = 0.6179, *n* = 3). **(D)** Fluorescence quantitative analysis displayed that decreased Ca^2+^ influx emerged in morphine-treated neurons compared with control group 3 min after treatment with AC1903 in calcium-containing kreb’s buffer (3 min: Unpaired Student’s *t* test, *t* (4) = 1.185, *P* = 0.3016, *n* = 3). **(F)** Fluorescence quantitative analysis displayed that decreased Ca^2+^ influx emerged in morphine-treated neurons compared with control group 4 min after treatment with AC1903 in calcium-containing kreb’s buffer (Unpaired Student’s *t* test, *t* (4) = 0.1037, *P* = 0.9224, *n* = 3). **(H)** Fluorescence quantitative analysis displayed that decreased Ca^2+^ influx emerged in morphine-treated neurons compared with control group 5 min after treatment with AC1903 in calcium-containing kreb’s buffer (5 min: Unpaired Student’s *t* test, *t* (4) = 0.2917, *P* = 0.7850, *n* = 3). **(I)** Primary spinal neurons were exposed to RLZ (50 μM, 2 h) in the presence or absence of EGTA and BAPTA-AM. The levels of extracellular GABA were detected by ELISA (one-way ANOVA, *F* (2, 21) = 40.04, *P* < 0.0001, *n* = 8). Data are expressed as mean ± SD, ****P* < 0.001.


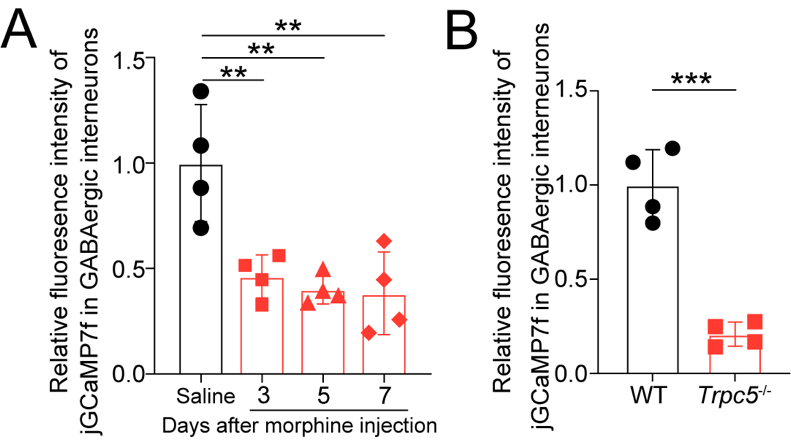


**Figure. S15. Quantitative analysis of calcium levels. (A)** Quantitative analysis revealed a significant reduction in calcium levels within spinal GABAergic inhibitory interneurons on days 3, 5, and 7 following intrathecal morphine administration (one-way ANOVA, *F* (3, 12) = 10.58, *P* = 0.0011, *n* = 4). **(B)** Data showed decreased calcium levels in spinal GABAergic interneurons of *Trpc5*^-/-^ mice compared to WT mice (Unpaired Student’s *t* test, *t* (6) = 7.977, *P* = 0.00027, *n* = 4). Data are expressed as mean ± SD, ***P* < 0.01 and ****P* < 0.001.


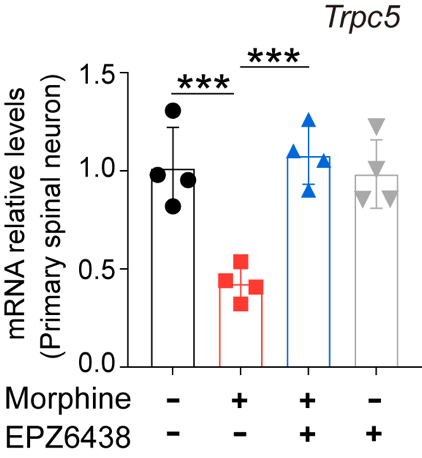


**Figure. 16. The effect of EPZ6438 on *Trpc5* transcription in primary spinal neuron.** Primary spinal neurons were pretreated with EPZ6438 (1 μM, 72 h) and then subjected to morphine (200 μM, 14 h). The samples were collected for qPCR. EPZ6438 abrogated the effect of morphine on the *Trpc5* transcription (one-way ANOVA, *F* (3, 12) = 14.16, *P* = 0.0003, *n* = 4). Data are expressed as mean ± SD, ****P* < 0.001.

**
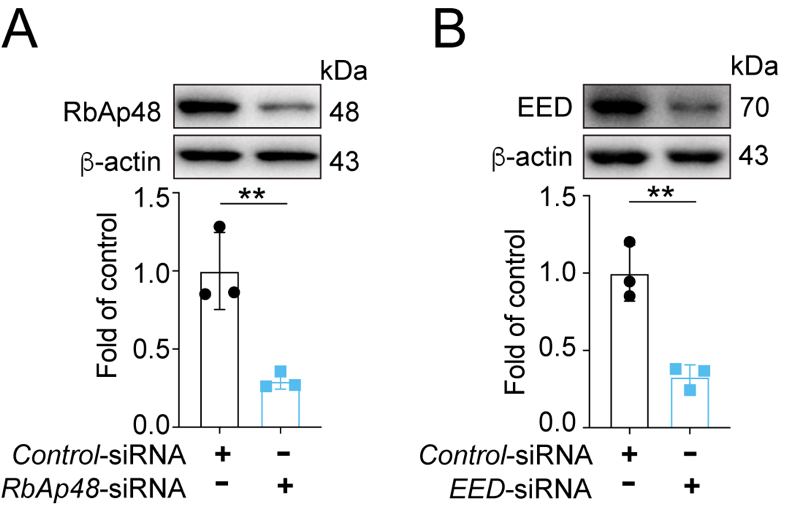
**

**Figure. S17. Western blot analysis for validation of *RbAp48* siRNA and *EED* siRNA in SH-SY5Y cells.** **(A and B)** SH-SY5Y cells were transfected with *RbAp48* siRNA and *EED* siRNA. The efficiency of transfection was verified by immunoblot (RBAP48: Unpaired Student’s *t* test, *t* (4) = 4.830, *P* = 0.0085, *n* = 3; EED: Unpaired Student’s *t* test, *t* (4) = 5.905, *P* = 0.0041, *n* = 3). Data are expressed as mean ± SD, ***P* < 0.01.

**
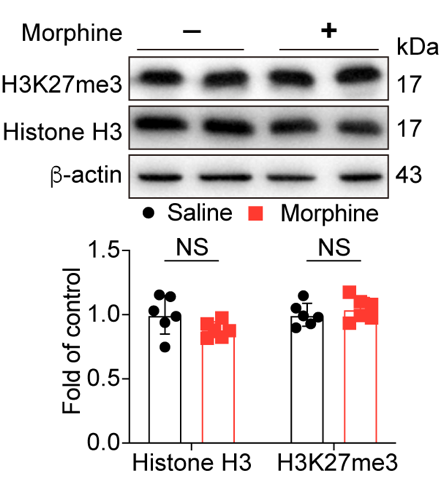
**

**Figure. S18. Intrathecal injection of morphine does not affect the levels of H3K27me3 in spinal cords.** Mice were intrathecally injected with morphine (10 μg/10 μL) for 7 consecutive days to establish morphine tolerance model. The spinal cords L4-L5 were collected for immunoblotting. Chronic morphine exposure did not change the levels of H3K27me3 (two-way ANOVA, drug effect: *F* (1, 20) = 0.7406, *P* = 0.3997; time effect: *F* (1, 20) = 3.622, *P* = 0.0715, drug × time effect: *F* (1, 20) = 3.622, *P* = 0.0715, *n* = 6). Data are expressed as mean ± SD.


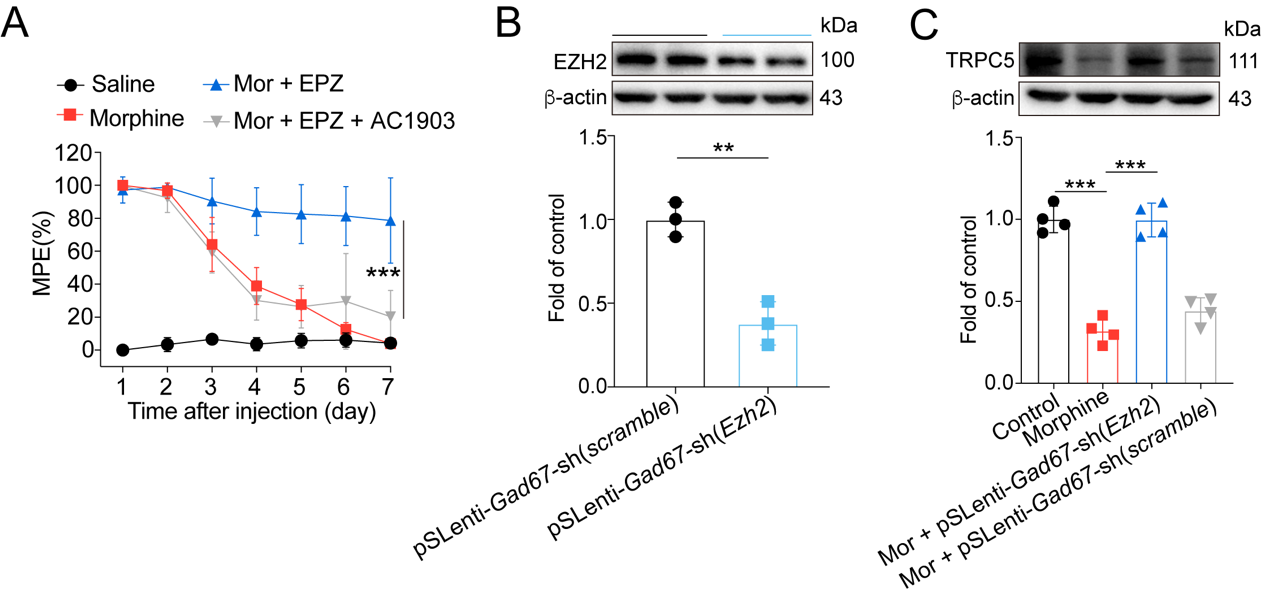


**Figure. S19.** **AC1903 abrogates the effect of EPZ6438 on morphine tolerance, and the knockdown of EZH2 prevents morphine-induced suppression of TRPC5 in primary spinal neurons. (A)** AC1903 (i.t., 2 μg/10 μL) abolished the effect of EPZ6438 on morphine tolerance (two-way ANOVA, drug effect: *F* (3, 196) = 450.4, *P* < 0.0001; time effect: *F* (6, 196) = 83.96, *P* < 0.0001; drug × time effect: *F* (18, 196) = 21.21, *P* < 0.0001, *n* = 8 mice). **(B)** Primary spinal neurons were subjected with pSLenti-shRNA (*Ezh2)* or its control vector for 96 h. Immunoblotting data showed that pSLenti-shRNA (*Ezh2*) suppressed the EZH2 expression (Unpaired Student’s *t* test, *t* (4) = 6.496, *P* = 0.0029, *n* = 3). **(C)** Primary spinal neurons were subjected to pSLenti-shRNA (*Ezh2*) or its control vector for 96 h and then exposed to morphine (200 μM, 14 h). Data showed that the deficiency of EZH2 could abolish morphine-induced suppression of TRPC5 (one-way ANOVA, *F* (3, 12) = 70.09, *P* < 0.0001, *n* = 4). Data are expressed as mean ± SD, ***P* < 0.01 and ****P* < 0.001.


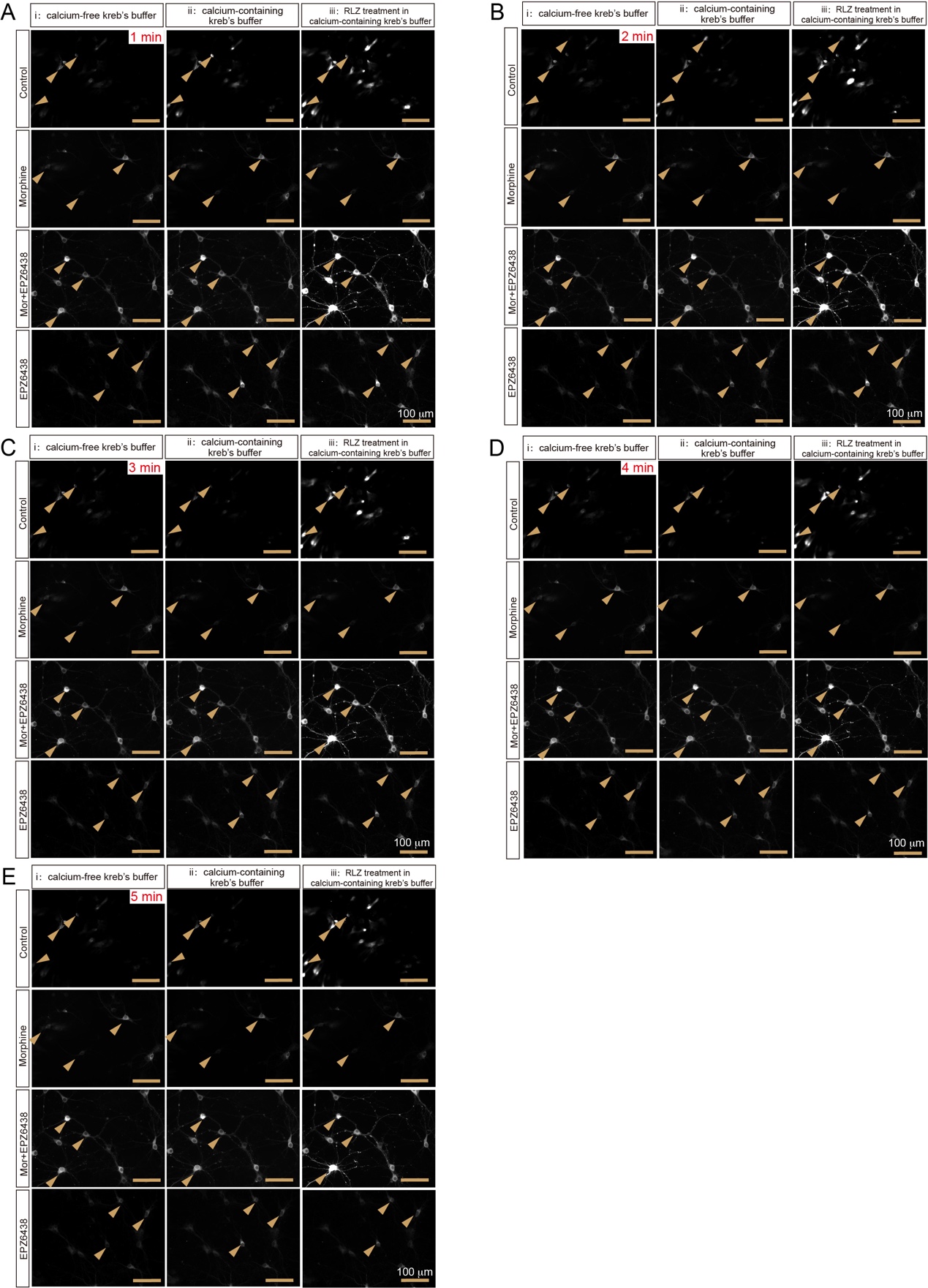


**Figure. S20. Calcium imaging of the effect of EPZ6438 on morphine-induced decreased Ca^2+^ influx in primary spinal neurons from 1 min to 5 min.** Primary spinal neurons were cultured and transduced with pSLenti-Gad67-jGCaMP7f-Puro-WPRE 24 h prior to EPZ6438 treatment (1 μM, 72 h). Neurons were then exposed to morphine (200 μM, 14 h) in the presence of EPZ6438. After morphine removal, the medium was replaced with Krebs’ buffer supplemented with an L-type calcium channel inhibitor (nifedipine, 1 μM) and an N-type calcium channel inhibitor (Cav2.2 blocker 1, 10 μM). (A-E) Representative images show sequential treatments: (i) neurons incubated in calcium-free Krebs’ buffer; (ii) subsequent incubation in calcium-containing Krebs’ buffer for 5 min, with representative images capturing Ca^2+^ influx at 1-5 min, respectively; (iii) addition of RLZ in calcium-containing Krebs’ buffer, with representative images displaying Ca^2+^ influx at the corresponding time points (1-5 min).


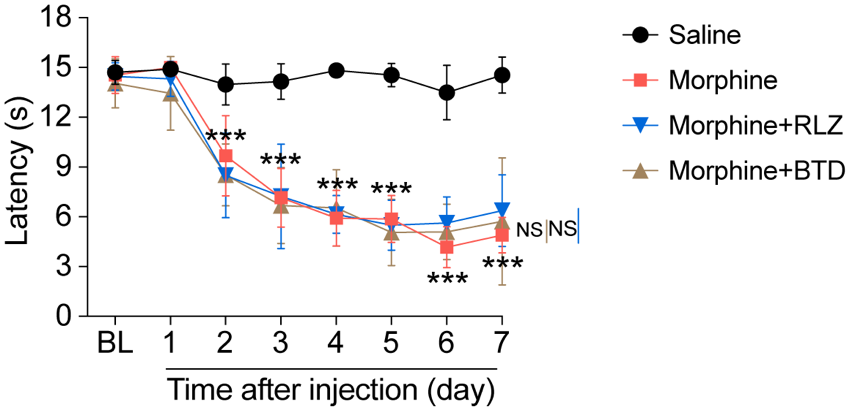


**Figure. S21. Intrathecal injection of TRPC5 activators has no effect on morphine-induced hyperalgesia in mice.** Mice were subcutaneously injected with saline or morphine (10 mg/kg) twice daily for 7 consecutive days. Behavioral testing was conducted every morning by tail-flick assay 30 min after RLZ and BTD injection (i.t., 2 μg/ 10 μL), followed by morphine administration (two-way ANOVA, drug effect: *F* (3, 160) = 144.6, *P* < 0.0001; time effect: *F* (7, 160) = 69.02, *P* < 0.0001, drug × time effect: *F* (21, 160) = 7.037, *P* < 0.0001, *n* = 6). Morphine versus saline, ****P* < 0.001, Data are shown as mean ± SD.

**Supplementary Table 1**

*Patients without morphine-treatment baseline characteristics*

| Characteristic |  | Group  (N=8) |
| --- | --- | --- |
| Age (years) | <=50 | 1 |
|  | 51-60 | 2 |
|  | 61-70 | 3 |
|  | >=71 | 3 |
| Gender | Male | 4 |
|  | Female | 4 |
| Clinical diagnoses | Intracranial hemorrhage | 1 |
|  | Postherpetic neuralgia | 3 |
|  | Disturbance of consciousness | 3 |
|  | Myasthenia | 1 |

**Supplementary Table 2**

*Patients with morphine-treatment baseline characteristics*

| Characteristic |  | Group  (n = 8) |
| --- | --- | --- |
| Age (years) | <=50 | 3 |
|  | 51-60 | 3 |
|  | 61-70 | 2 |
| Gender | Male | 2 |
|  | Female | 6 |
| Clinical diagnoses | Colon cancer | 1 |
|  | Esophagus cancer | 2 |
|  | Glandular cancer | 1 |
|  | Breast cancer | 1 |
|  | Nasopharynx cancer | 1 |
|  | Lung cancer | 1 |
|  | Pain in both lower limbs after limb surgery | 1 |
| Analgesic agents | Ropivacaine combination with morphine | 1 |
|  | Morphine | 4 |
|  | OxyContin | 3 |

**Supplementary Table 3**

*Mouse qPCR primers*

| Target gene | Primer sequence (5’-3’) |
| --- | --- |
| *Trpc5* | GGGCTGAGACTGAGCTGTC |
| *Trpc5*-rev | TTGCGGATGGCGTAGAGTAAT |
| *Adnp* | ACGAAAAATCAGGACTATCGG |
| *Adnp*-rev | GGACATTCCGGAAATGACT |
| *Atp2a3* | CTCTGACTTGCCTGGTGGAGA |
| *Atp2a3*-rev | GGTGAACTCCTTCCGCATCA |
| Adcy9 | GCAAAATGGCTGTCAAGACGAGC |
| Adcy9-rev | CTGGCTGTTAGTGAGCTTCTCC |
| *Htra2a* | TAATGCAATTAGGTGACGACTCG |
| *Htra2a*-rev | GCAGGAGAGGTTGGTTCTGTTT |
| *Prkg1* | ACAACTGTACCCGGACAGCGA |
| *Prkg1*-rev | TCCTCTTGCACCCTGCCTGAT |
| *Hspa1b* | TTGTCCATGTTAAGGTTTTGTGGTATA |
| *Hspa1b*-rev | GTTTTTTTCATTAGTTTGTAGTGATGCAA |
| *Pcdhga8* | CTGGTGCTAGAGCGCACTC |
| *Pcdhga8*-rev | CCGGTTGGTCAAAAACAGGG |
| *Pcdhga1* | GATCCGCATCGTTTCCAGAGG |
| *Pcdhga1*-rev | TCTCTACTTCGATAGGGAGAAGC |
| *Pcdhgb1* | CATTTTCCACGTAACGGTTGC |
| *Pcdhgb1*-rev | CCCTGCTAAGGCTGACTCAC |
| *Lnpep* | GACAAAGACCGAGCCAACCTGA |
| *Lnpep*-rev | GAACAGGGCTTCAGTGATCGGT |
| *Aff2* | ATTATGCTACCGATGCTTGTCAC |
| *Aff2*-rev | CATTGCCTACCCAAGGAGATG |
| *Gabrb* | ATGTCGCTGGTTAAAGAGACG |
| *Gabrb*-rev | CTGCCACTCGGTTGTCCAAA |
| *Plin4* | GACCAGCAGTGAAGATGCCT |
| *Plin4*-rev | TCCTTCGTATTGGTGAGGACA |
| *Erdr1* | CAGTGATGTCACCCACGAAA |
| *Erdr1*-rev | GGCATTTCTGTACGCAGTCA |
| *Rad9b* | AGGTGAGATGACAGGAGATC |
| *Rad9b*-rev | CTTGGGTGGAGAGGCTATTC |
| *Cd72* | GGACCAGAGGTTGACAAGCAC |
| *Cd72*-rev | TTCCGAGGGTATGTGAGATGTAA |
| *Pcsk4* | CTGGGACAGATCTTCCCT |
| *Pcsk4*-rev | GGTTCTCATCGTTGGGTGTGTA |
| *Hif3a* | GTCGGAGAGTATCGTCTGTGTC |
| *Hif3a*-rev | TCTGCGAGAGTGTTGCTCCGTT |
| *β-actin* | CATTGCTGACAGGATGCAGAAGG |
| *β -actin*-rev | TGCTGGAAGGTGGACAGTGAGG |

**Supplementary Table 4**

*Rat qPCR primers*

| Target gene | Primer sequence (5’-3’) |
| --- | --- |
| *Trpc1* | TACGGTTG TCAGTCCGCAGA |
| *Trpc1*-rev | TCGTTTTGGCCGATGATTAAGTA |
| *Trpc4* | GTGTGCTACCTGATAGCTCCC |
| *Trpc4*-rev | GGCAGAGACACGTTCGTTATT |
| *β -actin* | CTAGGAGCCAGGGCAGTAATCT |
| *β -actin*-rev | AAGACCTCTATGCCAACACAGTG |

**Supplementary Table 5**

*Human TRPC5 promoter sequence (5’-3’)*

ACATAGCTACTACCAGCTTCACAGGCTCCGCAATGAAAACACTCGGCCATTTAGGCAAGGGAGATGATTCTGGCATTTTCGGGAGCTTACACTTAGGCTCCCAGGGCGTGCAGAGGGGGTTAAAGAGCAACAGGAAGTACAGTGGAGTTTTCTCAGAGCTAGGGATTGTTCTGAGAATGGGTAGGGAAAGGTTTTCAGGTACCAGCCCGGCGTCCAGAAAGGCAGAGACTCACATGCCCCTTTCCTGAGAGCGCTAGGAAGGGAGATAAAAGACAAAGCCAATATTTGGGGCTGTGCGGCCAGGCCATGAAATGCTGCCAGCTAGTGGACCTATGCGCCTGCAGGTTGCAGGTAGAAGTCTCAGGCTTGTGGTGGGGGTGGGGCTATTTAGCCCCTTCCAGTTGGGCTCAAGAGGGTGGACATTCTGAGCGCCAGGCTATAAGGTGAAAGAAGTGGAGAGGTGCTTCATGTGAAAGGAAGGCGTAAAGATCAAAGAGTGGCCCAAGTGTCCGGTGACACCGCCCCTCTCCCTCCACAACAGATACCACTAAGCAACCTAGCCCCCCGCCCCCATCCTACAATATCACTTTCCCTCCCTGTATCAATCTAGGCTGGATCTTGGGAGATCGACCCTATGGGTGAGAGGGGCACGGATGGGAGAATTAGGTAGCTCCAACTAGATTAATCTAGATCTATCAGGGATCCAAGCCTGCCCCATAGGACCCCAGGTCAGTCCCTGGCGACTCAGCGCTCCGCTGTCTCCCTGTCCTACCAGCCGCCCGCCGCCCTTACTCCCCCAGGTTCAACTCCCCTTCCTCCTGAATGACCTATGGTCGCCGGAGCAGCCTCGCCACCACCGGCCCAGAGCCCAGGCTGACGGGGGTGGGCAAAGGTCACTGCCCAAAGCAGGGGATATTCAGGCCAAGGGGCAAAGAGAGACTCTGCTAGCCATATGCTCCGAGGGACTCCTGTCCCCTCTACCCGCACCCGCGCTTAGCCTCCCACCTCCCCTCCCGTCTCTGCCACCACCCAGGGGAGGGGGAGGTCTGGGGAAGCAAACAAACGAGACAGAAAATTCCGTACCGAGGGGGGAGAGATGAGACGGTGGGACGGGGCAGCGGGGGTTGACTTGGGGCTGCGGCTGCGGCTTCCTTCCACCCCTGGCCGCGGAGCTGCAAAGAAGGCAGGAGCTGCTGCCCCTGCCGCCTGCCCCGTCCTCCCTCTTTCCCTCTCGCCTCCCCGCCTCCCCCGCCCCGCCCCGCCGCCAGGAGATTGAGAGTGGCTTCGGCCCGGGGGCTGGGGGAGGTGAGGGGTGCTTTTGCCTACCACGGTTGTCTCAATCTCTCTCTCTCTCTCTCTCTCTCTTTCTCTCTCTCTCTCTCTCTCTCTCTCTCTCTCTCTCACACACACACACACACACACACACCCCACATCACAACCACGACGGGTTGTCCGCAGCTCGCAGCCACCCCAAAGGGAGGAATAAAGAACAGGAGCAGAGGGAAGCCGGGCGCAGAAAGAAGGGAAAAGGAAGAGCTCAGGGTTGGACATTTTTGGAGACAGGGGTGGGGAGGATAAAGGGAGCGTGCCTCCTTGATTTTCCCCTCCCCACCCCTCTTTGCTTTTCTCTCCCCTTCTCTGTATAGCATATTCCAGAAACGGCAGCAGAAGGGGACAGAAACCTCTAGTGTAGAAAAGGGTTTTGGAGGGGTAGCCAGCGCTCCCGCCAGCCGCTACCTAACAGCTGGAGCTTGAGTGTGCGTGTAAGAGTGTGAATGCGGCTCTCTCTCTTTTTTTCTC

**Supplementary Table 6**

*Human qChIP PCR primers*

| Target gene | Primer sequence (5’-3’) |
| --- | --- |
| *TRPC5* | TGGCATTTTCGGGAGCTTAC |
| *TRPC5*-rev | GCTGGTACCTGAAAACCTTTCC |
